# Maturation and culture affect the metabolomic profile of oocytes and follicular cells in young and old mares

**DOI:** 10.3389/fcell.2023.1280998

**Published:** 2024-01-12

**Authors:** D. R. Bresnahan, G. D. Catandi, S. O. Peters, L. J. Maclellan, C. D. Broeckling, E. M. Carnevale

**Affiliations:** ^1^ Department of Animal Sciences, Berry College, Mount Berry, GA, United States; ^2^ Department of Biomedical Sciences, College of Veterinary Medicine and Biomedical Sciences, Colorado State University, Fort Collins, CO, United States; ^3^ Proteomic and Metabolomics Core Facility, Colorado State University, Fort Collins, CO, United States

**Keywords:** mare, oocyte, cumulus, granulosa, culture, maturation, follicle, metabolome

## Abstract

**Introduction:** Oocytes and follicular somatic cells within the ovarian follicle are altered during maturation and after exposure to culture *in vitro*. In the present study, we used a nontargeted metabolomics approach to assess changes in oocytes, cumulus cells, and granulosa cells from dominant, follicular-phase follicles in young and old mares.

**Methods:** Samples were collected at three stages associated with oocyte maturation: (1) GV, germinal vesicle stage, prior to the induction of follicle/oocyte maturation *in vivo*; (2) MI, metaphase I, maturing, collected 24 h after induction of maturation *in vivo*; and (3) MIIC, metaphase II, mature with collection 24 h after induction of maturation *in vivo* plus 18 h of culture *in vitro*. Samples were analyzed using gas and liquid chromatography coupled to mass spectrometry only when all three stages of a specific cell type were obtained from the same mare.

**Results and Discussion:** Significant differences in metabolite abundance were most often associated with MIIC, with some of the differences appearing to be linked to the final stage of maturation and others to exposure to culture medium. While differences occurred for many metabolite groups, some of the most notable were detected for energy and lipid metabolism and amino acid abundance. The study demonstrated that metabolomics has potential to aid in optimizing culture methods and evaluating cell culture additives to support differences in COCs associated with maternal factors.

## Introduction

Oocyte quality is essential for normal fertility. However, studying the oocyte that is destined for ovulation is difficult in monovular females, such as the mare and woman. The mare provides unique opportunities to investigate the ovarian follicle and oocyte, as the preovulatory follicle is large, approximately 45 mm in diameter, and can be readily tracked using ultrasonography. In addition, follicle cells and oocytes can be collected from the mare’s dominant follicle at specific intervals during follicle growth and oocyte maturation for timed analyses (Campos-Chillon et al., 2015). Similar to the women, the mare has a long follicular phase, and an oocyte maturation interval of approximately 36 h after administration of ovulation inducing compounds (Abbara et al., 2018), and a notable decline in fertility with maternal aging [reviewed in Carnevale (2008)]. Consequently, studies of the equine follicle can provide valuable information for the domestic horse, as well as provide novel insight into factors affecting oocyte and follicular viability in the woman and other species, including endangered equids and rhinoceros (Meuffels-Barkas et al., 2023).

Maturation of the oocyte naturally occurs within the ovarian follicle, with fertility dependent on the coordinated progression of maturation of the follicle and oocyte prior to ovulation. Oocyte maturation can be broadly broken down into nuclear and cytoplasmic maturation. Although critical for acquisition of developmental competence, oocyte cytoplasmic maturation is challenging to assess. Studying the metabolome of the oocyte provides a potential method to determine how maternal or environmental factors affect oocyte cytoplasmic integrity. Nuclear maturation of the oocyte is easier to assess than cytoplasmic maturation. The oocyte nucleus is arrested at the first meiotic prophase in the germinal vesicle (GV) stage with dispersed chromatin; high levels of cAMP maintain the oocyte in this stage prior to the initiation of maturation (Jiang et al., 2023). After stimulation by endogenous luteinizing hormone (LH) or exogenous ovulation inducing compounds, gap junctions between follicular cells and the oocyte are disrupted, resulting in reduced cAMP within the oocyte and GV breakdown with gradual chromatin condensation, nuclear membrane disintegration, and progression to metaphase I (Tukur et al., 2020). In the mare during midestrus, the dominant follicle typically reaches approximately 35 mm in diameter, with the oocyte remaining at the GV stage, although the oocyte/follicle can be induced to mature using exogenous human chorionic gonadotropin or a GnRH analog (Carnevale, 2016). Using maturation induction prior to the natural initiation of maturation, timing of oocyte maturation to meiosis I (MI) and meiosis II (MII) can be predicted allowing for study of oocyte maturation or appropriately timed *in vitro* fertilization procedures.

With the initiation of maturation, oocyte nuclear maturation progress to MI, with microtubules migration in proximity to the diploid chromosomes; the chromosomes align and form the metaphase plate (Conti and Franciosi, 2018). Nuclear maturation will ultimately progress to MII, with half the homologous chromosomes extruded as the first polar body. In the mare and women, ovulation occurs with the oocyte at MII (He et al., 2021). The oocyte reassembles the chromosome spindle, arrests at MII, and prepares to segregate sister chromatids into the second polar body and resume the second meiosis upon fertilization (Conti and Franciosi, 2018). The MII oocytes are considered mature and ready for fertilization.

Oocyte developmental potential and quality is dependent on follicular and maternal factors. In the follicle, the oocyte is dependent on somatic follicular cells, especially granulosa and cumulus cells. Granulosa cells line the follicular wall, participate in steroidogenesis along with theca cells, and are crucial for transport of components from the systemic circulation into the follicular fluid (Siu and Cheng, 2013). Cumulus cells are a specialized subgroup of granulosa cells that surround, protect, and connect to the oocyte through cellular transzonal projections and gap junctions; therefore, cumulus cells are responsible for directly transferring energy substrates and signaling molecules to the oocyte (Martinez et al., 2023). Oocytes and cumulus cells form a metabolically co-dependent structure, the cumulus oocyte complex (COC), in which the oocyte communicates its metabolic needs via secretion of growth factors that target cumulus cells to stimulate metabolism and transfer substrates to the oocyte (Richani et al., 2021). Oocytes or cumulus cells are not metabolically competent when cultured independently, emphasizing their metabolic co-dependence (Richani et al., 2021). Therefore, granulosa and cumulus cell metabolomes could provide important information as to follicle function and the associated viability of the oocyte.

Maternal status can also affect oocyte quality, potentially through factors associated with the follicular cells and their metabolic capacity. One of the most pronounced maternal factors affecting oocyte quality is aging, which can impact the ovarian follicular environment, communication between the oocyte and follicular cells, and ultimately oocyte quality (Campos-Chillon et al., 2015; El-Hayek et al., 2018; Krisher, 2019; Alberico and Woods, 2022). Maternal aging is well documented to reduce fertility in women specifically due to reduced oocyte quality (Krisher, 2019). In mares, similar findings have been observed and have been directly associated with oocyte quality (Carnevale et al., 1993; Frank et al., 2019; Carnevale et al., 2020). The extent that maternal age affects the metabolome during maturation is not well documented.

For assisted fertilization technologies, cumulus oocyte complexes are typically exposed to culture media and conditions for a variable amount of time. However, not enough is known about how exposure to culture or holding medium affects follicle cells and oocyte metabolism. Mare preovulatory follicular fluid composition differs from commonly used culture media for equine cumulus oocyte complexes (Fernández-Hernández et al., 2020), and cumulus cell metabolome and proteome differ after maturation *in vivo* or *in vitro* in mares (Walter et al., 2019). However, most studies assessing culture media use immature oocytes, which can be variable in status, and *in vitro* maturation with different hormone regimes to promote nuclear maturation; all factors that can affect cytoplasmic maturation. For the mare, we can induce oocyte/follicle maturation *in vivo*, collect the maturing oocyte and culture for the completion of oocyte maturation in a manner that has proven to provide similar early pregnancy rates as ovulated oocytes (Carnevale et al., 2004) and is successful for *in vivo* or *in vitro* embryo production, establishment of pregnancies, and production of live foals (Carnevale et al., 1993; Carnevale et al., 2005; Carnevale et al., 2010; Frank et al., 2019). The extent that culture or holding of cumulus oocyte complexes in media effects the metabolome is not well established.

In the present study, we investigated the effects of maturation and culture on the metabolomic profiles of equine oocytes and follicular cells from young and old mares. To minimize the effect of individual follicles, only oocytes from dominant, follicular-phase follicles (anticipated ovulatory follicles) were used, and samples of a specific cell type (oocytes, cumulus cells or granulosa cells) were obtained for analyses from the same individual mares during three times associated with oocyte maturation stages (GV, MI and MII). For the final stage (MII), oocytes were allowed to mature to MI *in vivo*, before collection and culture to MII *in vitro*. Therefore, exogenous hormonal stimulation was not required to complete oocyte maturation, and we could more appropriately assess the cellular effects of exposure to a culture medium for a limited time interval. Our first aim was to determine changes in the metabolomic profiles of oocytes, cumulus cells, and granulosa cells during maturation and after exposure to culture medium, and our second aim was to determine if oocytes and follicular cells from young and old mares respond differently over three stages of maturation and/or after culture.

## Materials and methods

### Study design

Oocytes, cumulus cells, and granulosa cells were collected from individual, follicular-phase, dominant follicles and compared over three stages, consistent with oocyte/follicle maturation, for young mares and old mares. Maturation stages were collected at: 1) germinal vesicle (GV), at the anticipated germinal vesicle stage of oocyte development with no induction of follicle maturation, 2) meiosis I (MI), at approximately 24 h after follicle maturation induction and at the anticipated metaphase I stage, and 3) meiosis II following cell culture (MIIC), collection at 24 h after follicle maturation induction with an additional 18 h of culture to assure maturation to metaphase II. For the study, samples were only used when all stages (GV, MI and MIIC) for a specific cell type (oocyte, cumulus cells, or granulosa cells) were obtained from the same mare.

### Animals and study design

All animal procedures were approved by the Colorado State University’s Institutional Animal Care and Use Committee. Follicular cell samples were collected from young mares (Young, 7–11 years, mean age of 10 ± 0.6 years, n = 8) and old mares (Old, 19–28 years, mean age of 23 ± 0.8 years, n = 12) during the natural equine breeding season. Mares were housed by age in adjacent dry lots with access to covered shelters. Mares were fed a mixture of grass and alfalfa hay at approximately 2% body weight daily, with mineral salt and water *ad libitum*. After all samples were collected, only sample types (oocyte, cumulus cells, or granulosa cells) from individual mares that had a sample collected during each stage (GV, MI, MIIC) were used for statistical comparisons. However, metabolic profiles from all samples for each specific time collection were compared for mare age in a separate publication (Catandi et al., 2023).

### Sample collections from follicles

To assess follicular growth and stage of the estrous cycle, mares’ reproductive tracts were examined by transrectal ultrasonography. Transvaginal, ultrasound-guided, follicular aspirations of dominant follicles were performed as previously described (Carnevale, 2016) to collect follicular contents. Oocytes and follicular cells were only collected from the large, dominant follicle during the follicular phase. Because of the long estrous interval in mares, the dominant follicle that is destined for ovulation can be identified prior to the initiation of oocyte maturation, providing for consistent follicle development status and timing of maturation. Germinal vesicle stage oocytes (GV) were collected from follicles ≥35 mm in diameter when endometrial edema indicative of estrus was observed and without induction of follicular maturation. The cumulus oocyte complex and granulosa cells were assessed to assure compact cells, consistent with an immature follicle; and no perivitelline space or polar body was confirmed for oocytes. For MI and MII samples, follicular maturation was induced by administration of human chorionic gonadotropin (2000 IU, intravenous; Chorulon, Merck Animal Health, Madison, NJ) and deslorelin acetate in an aqueous base (0.75 mg, intramuscular; Precision Pharmacy, Bakersfield, CA) when follicles and endometrial edema were consistent with GV collections. Oocytes and follicle cells were collected from follicles at 24 ± 2 h after induction, and the samples were assessed for expanding cumulus and granulosa cells, consistent with a maturing follicle. Attached granulosa cells were trimmed from the cumulus oocyte complex. For MIIC samples, cumulus oocyte complexes and sheets of granulosa cells were incubated separately in tissue culture media 199 with Earle’s salts (GibcoTM, Thermo Fisher, Waltham, MA) with added 10% fetal calf serum, 25 μg/mL of gentamicin, and 0.2 mM pyruvate at 38.2°C in an atmosphere of 5% CO_2_ and air for 18 ± 2 h to allow completion of maturation. This maturation method and timing consistently results in the completion of maturation to metaphase II and good developmental potential of oocytes (Frank et al., 2019). For each stage, oocytes were denuded of cumulus cells using sequential pipetting in a MOPS-buffered medium (G-MOPS™, Vitrolife, Englewood, CO) containing 0.04% bovine serum albumin (BSA; Sigma-Aldrich, St Louis, MO) and hyaluronidase (200 IU/mL; Sigma-Aldrich). The denuded oocytes were evaluated under a stereoscope to confirm complete removal of cumulus cells and expected morphology for stage, including extrusion of the first polar body for oocytes at MII. The oocytes, cumulus cells, and granulosa cells obtained from individual follicles were washed and placed into 20 μL (granulosa cells and cumulus cells) or 10 μL (oocytes) of 50% methanol solution. Samples were stored in glass vials at −80°C until analyses.

### Oocyte, cumulus and granulosa cell metabolite extraction and detection using liquid and gas chromatography coupled to mass spectrometry

Samples were thawed to 4°C three times before adding 250 μL of 100% methanol, sonicated (QSonica ultrasonic processor) at 65% amplitude for 10 min, before being vortexed for 2 h at 4°C. After centrifugation (3,000 × g at 4°C), two aliquots of 120 μL of extract were transferred into 2-mL glass vials and dried under nitrogen gas for mass spectrometry analyses by liquid chromatography (LC-MS) and gas chromatography (GC-MS). Because granulosa and cumulus cells samples had various cell numbers, the remaining sample pellet was used for quantification of protein by reconstitution with urea and measurement of absorbance at 280 nm using a NanoDrop™ spectrophotometer (Thermo Fisher). Two distinct drops were measured in triplicate for each sample; highest and lowest readings were not used; the remaining values were averaged.

For LC-MS analysis, extracts of granulosa or cumulus cells were resuspended in volumes proportional to their protein content (5 μL of 100% methanol was used per 5 μg/μL of protein content) and with a minimum volume of 25 μL. Oocyte samples had a protein concentration less than 5 μg/μL; therefore, oocyte samples were resuspended in 25 μL of 100% methanol. Samples of the suspensions (2 µL) were injected onto a ACQUITY UPLC system (Waters, Milford, MA) using a randomized order and with a pooled quality control injection after every six sample injections; they were separated using a ACUITY UPLC CSH Phenyl Hexyl column (1.7 μM, 1.0 × 100 mm) (Waters) with a gradient from solvent A (A) (2 mM ammonium hydroxide, 0.1% formic acid) to solvent B (B) (99.9% acetonitrile, 0.1% formic acid). Injections were made in 100% A, held at 100% A for 1 min, ramped to 98% B over 12 min, held at 98% B for 3 min, and then returned to start conditions over 0.05 min and allowed to re-equilibrate for 3.95 min, with a 200 μL/min constant flow rate. The column and samples were held at 65°C and 6°C, respectively. The column eluent was infused into a Xevo G2 Q-TOF-MS (Waters) with an electrospray source in positive mode, with scanning at 50–2,000 m/z at 0.2 s per scan and alternating between MS (6 V collision energy) and MS^E^ mode (15–30 V ramp). Sodium iodide with 1 ppm mass accuracy was used for calibration. Capillary voltage was maintained at 2,200 V, with the source temperature at 150°C and nitrogen desolvation temperature at 350°C, with a flow rate of 800 L/h.

For GC-MS analysis, the extract was resuspended in pyridine containing 25 mg/mL of methoxyamine hydrochloride, with 5 μL per 5 μg/μL of protein for samples from granulosa and cumulus cells and 25 μL for oocytes, prior to incubation at 60°C for 60 min, vigorous vortexing for 30 s, sonicating for 10 min, and incubation for another 60 min at 60°C. The same volume of N-methyl-N-trimethylsilyltrifluoroacetamide with 1% trimethylchlorosilane (MSTFA +1% TMCS, Thermo Fisher) was added, and samples were vigorously vortexed for 30 s before incubation at 60°C for 30 min. Metabolite detection was done using a TRACE 1310 GC coupled to a ISQ™ mass spectrometer (ThermoFisher). Samples (1 μL) were injected at a 10:1 split ratio to a 30 m TG-5MS column (Thermo Fisher, 0.25 mm i.d., 0.25 μm film thickness) with a 1.2 mL/min flow rate of helium gas. The GC inlet was maintained at 285°C. The oven program started at 80°C for 30 s, followed by a ramp of 15°C/min to 330°C, and an 8 min hold. Masses between 50 and 650 m/z were scanned at 5 scans/s under electron impact ionization. Transfer line and ion source were maintained at 300°C and 260°C, respectively. Pooled quality control samples were injected following every 10 actual samples.

### Data acquisition and analysis

Raw data files for each sample were converted to a computable document format (.cdf); matrix of molecular features, defined by retention time and mass (m/z), was generated using XCMS in R software (BMC Bioinformatics) for feature detection and alignment (Su et al., 2008). Peak areas were subsequently quantile normalized in R. Outlier injections were determined based on total signal and PC1 of principle component analysis. Features were grouped using RAMClustR (Broeckling et al., 2014), grouping features into spectra based coelution and covariance across the full dataset; whereby, spectra are used to determine the identity of observed compounds. Peak areas for each feature in a spectrum were condensed using the weighted mean of all features in a spectrum into a single value for each compound. Compounds were annotated using spectral matching to in-house, NISTv14, Golm, HMDB and LipidMaps 1-SToP libraries (Broeckling et al., 2016), and Metlin metabolite databases. Data graphs and principal component analysis (PCA) plots were generated in GraphPad Prism 10.0. First, GC-MS and LC-MS datasets were concatenated together; unit variance scaling was applied to ensure signal intensities were in the same scale from the two platforms for plot generation.

### Statistical analyses

Samples (oocytes, cumulus cells or granulosa cells) for analyses were only included when GV, MI and MII samples from an individual mare were available for a given cell type. Traits within each cumulus, granulosa and oocyte cell type were analyzed by two-way mixed design analysis of variance (ANOVA) with the factor of age group (Young and Old) as the between-subjects factor and the factor of maturation stage group (GV, MI and MIIC) as the within-subject (repeated) factor to determine the main effects of age and stage and the effect of age-by-stage interaction (Mori et al., 2020). The descriptive statistics of traits within each cumulus, granulosa and oocyte cell type are presented as $\bar{y}\pm S_{\bar{y}}$ (mean ± standard error of the mean) for age, stage, and age-by-stage combination. All statistical analyses were carried out separately for the data from the cumulus, granulosa and oocyte cell types using the R function *anova_test*() *of rstatix* package in R-project software (R Project, 2023). Differences were considered significant at *p* < 0.05.

## Results

### Effects of maturation stage and cell culture on the global metabolome

Samples were only used when a specific cell type was collected for all stages (GV, MI and MIIC) from an individual mare. Oocytes were used from young mares (n = 8, mean age of 10 ± 0.6 years) and old mares (n = 9, mean age of 22.2 ± 0.7 years); cumulus cells were used from young mares (n = 8, mean age of 10 ± 0.6 years) and old mares (n = 6, mean age of 22.2 ± 1.1 years); granulosa cells were used from young mares (n = 5, mean age of 10.2 ± 0.9 years) and old mares (n = 9, mean age of 22.4 ± 0.9 years) for GV, MI and MIIC. However, for LC-MS results, only n = 8 samples were analyzed for oocytes and granulosa cells.

In oocytes, 48 annotated metabolites (Supplementary Table S1) and 86 unannotated metabolites differed (*p* < 0.05) for an effect of stage and/or interaction of stage-by-age and there was no difference in an additional 319 metabolites. For cumulus cells, 146 annotated (Supplementary Table S2) and 206 unannotated metabolites differed (*p* < 0.05) for the main effect of stage and/or interaction of stage-by-age with no difference in an additional 101 metabolites. In granulosa cells, 126 annotated metabolites (Supplementary Table S3) and 163 unannotated metabolites differed (*p* < 0.05) for stage and/or interaction of stage-by-age, and there was no difference in 164 metabolites. Main effects of mare age are noted in Supplementary Tables S1–S3.

**FIGURE 1:**
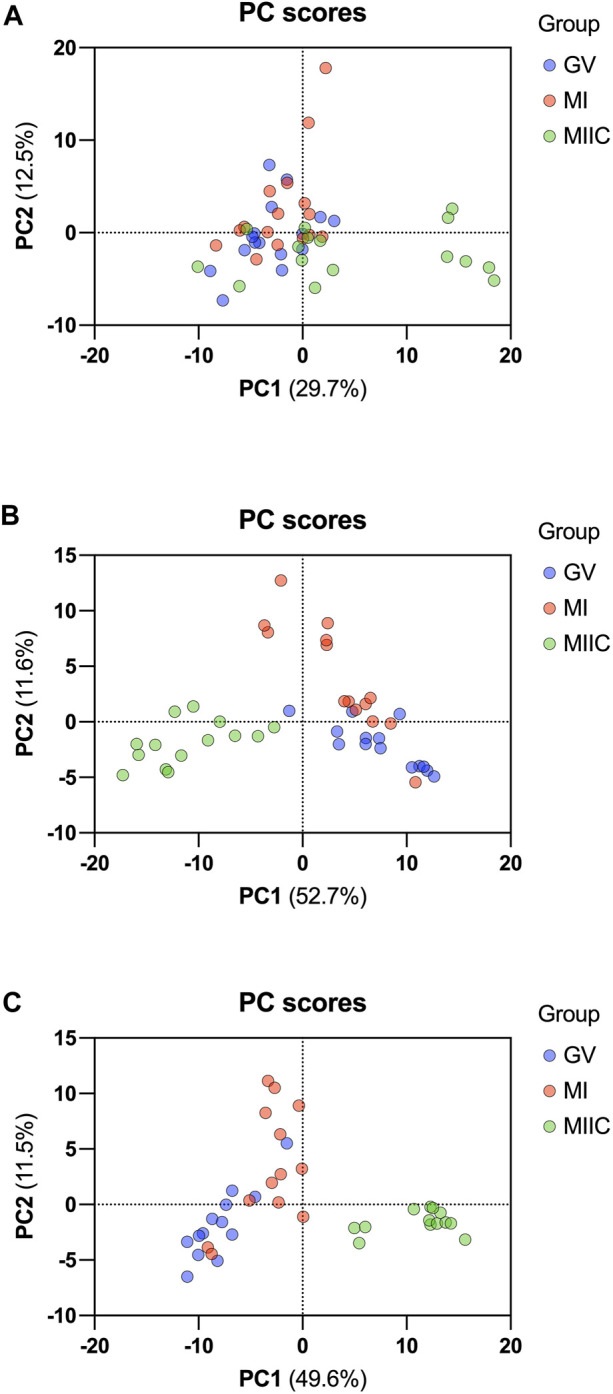
PCA score plots depicting the trend of separation of metabolites from Young and Old mares in **(A)** oocytes (n = 17), **(B)** cumulus cells (n = 14), and **(C)** granulosa cells (n = 14) corresponding to maturation stages *in vivo* (germinal vesicle, GV, blue and metaphase I, MI, red) and maturation with cell culture (metaphase II, maturation to MI *in vivo* plus 18 h of culture *in vitro*, MIIC, green).

**FIGURE 2:**
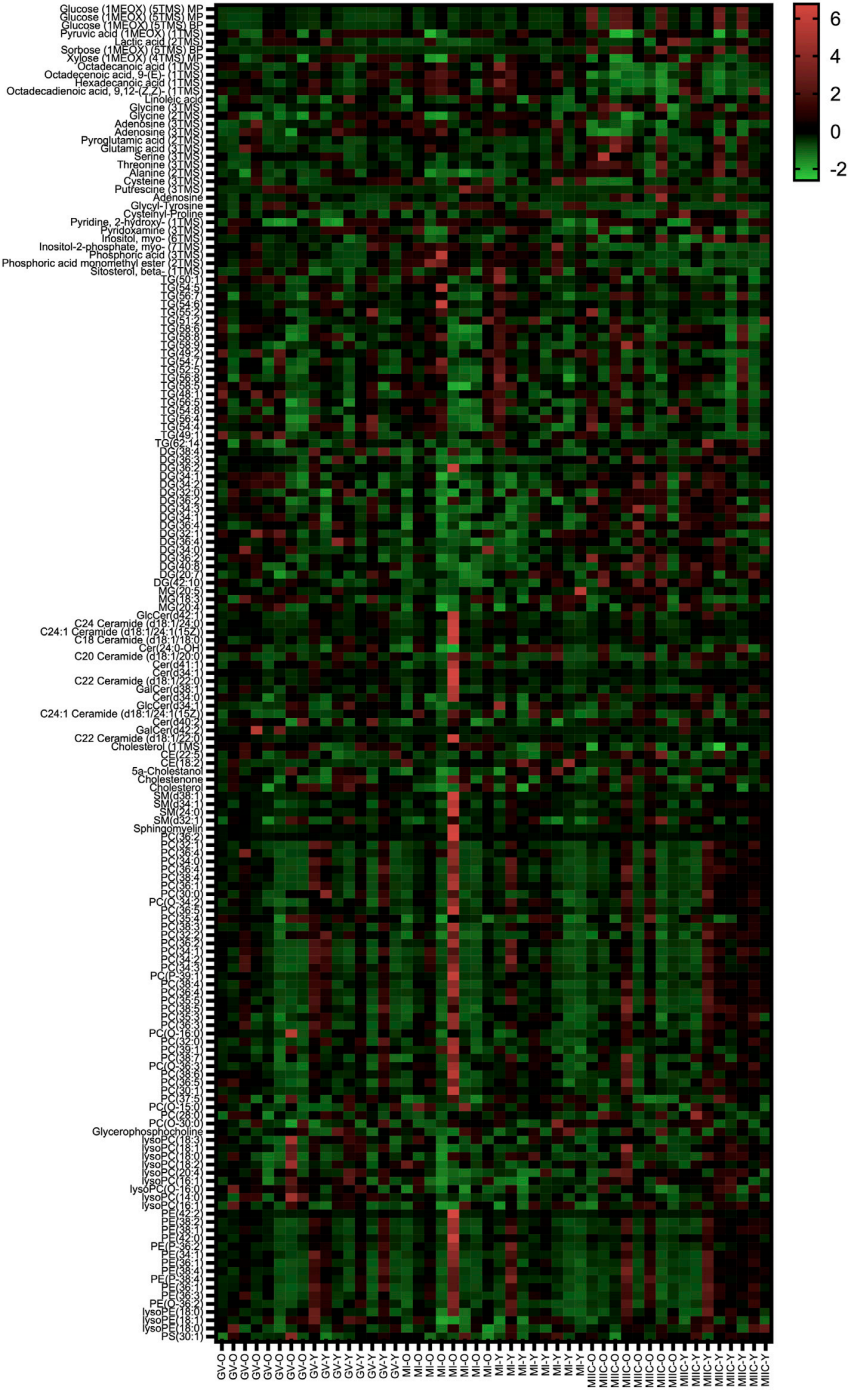
Heat map depicting variability in oocyte metabolite abundance at 0 h (GV, germinal vesicle stage) or 24 h (MI, metaphase I stage) after maturation induction, with additional oocytes collected as 24 h and cultured *in vitro* for 18 h (MIIC, metaphase II stage after culture). Green to red indicates greater relative abundance of metabolites. Y and O indicate samples from young or old mares, respectively.

**FIGURE 3:**
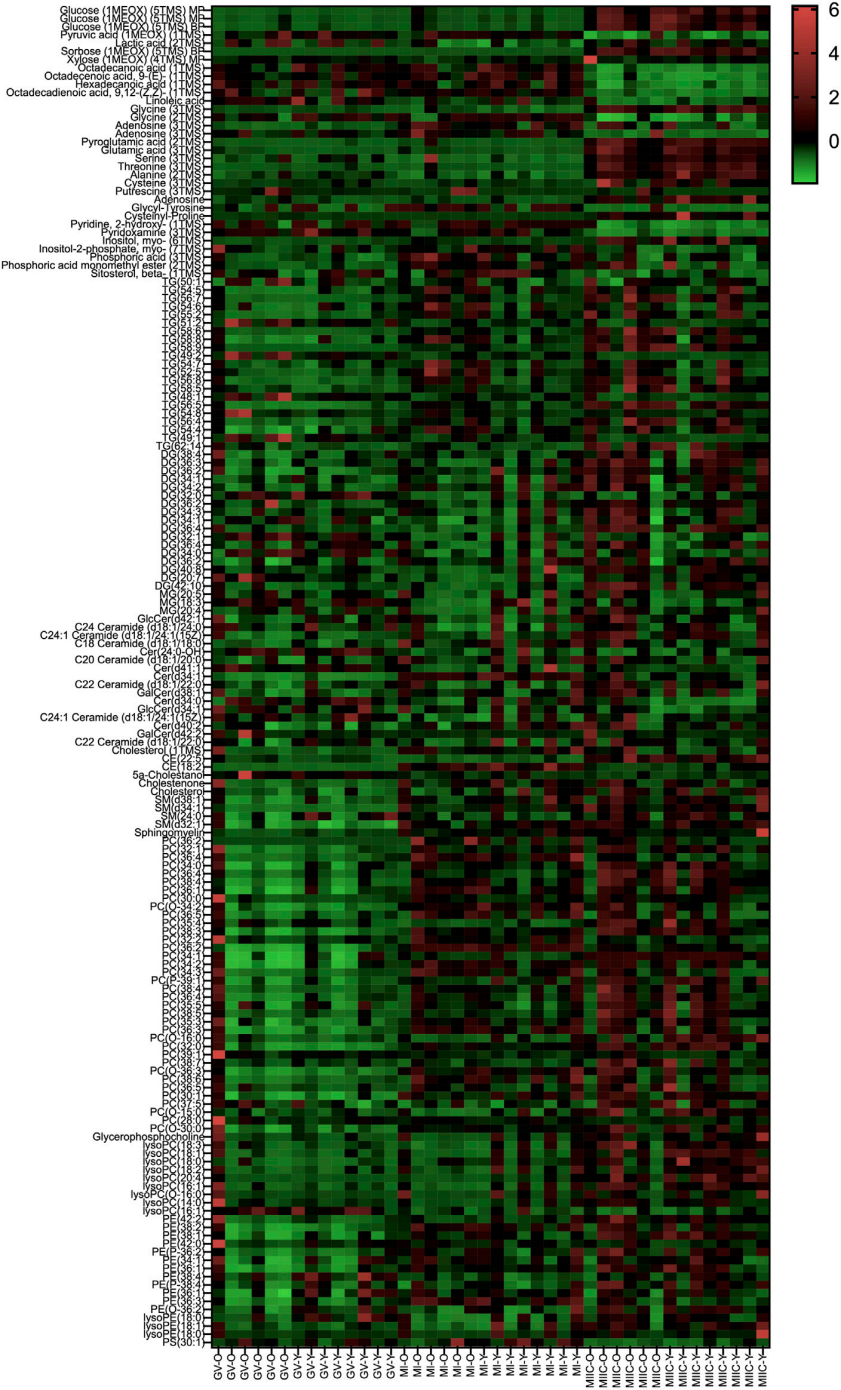
Heat map depicting variability in cumulus cell metabolite abundance at 0 h (GV, germinal vesicle stage) or 24 h (MI, metaphase I stage) after maturation induction, with additional oocytes collected as 24 h and cultured *in vitro* for 18 h (MIIC, metaphase II stage after culture). Green to red indicates greater relative abundance of metabolites. Y and O indicate samples from young or old mares, respectively.

**FIGURE 4:**
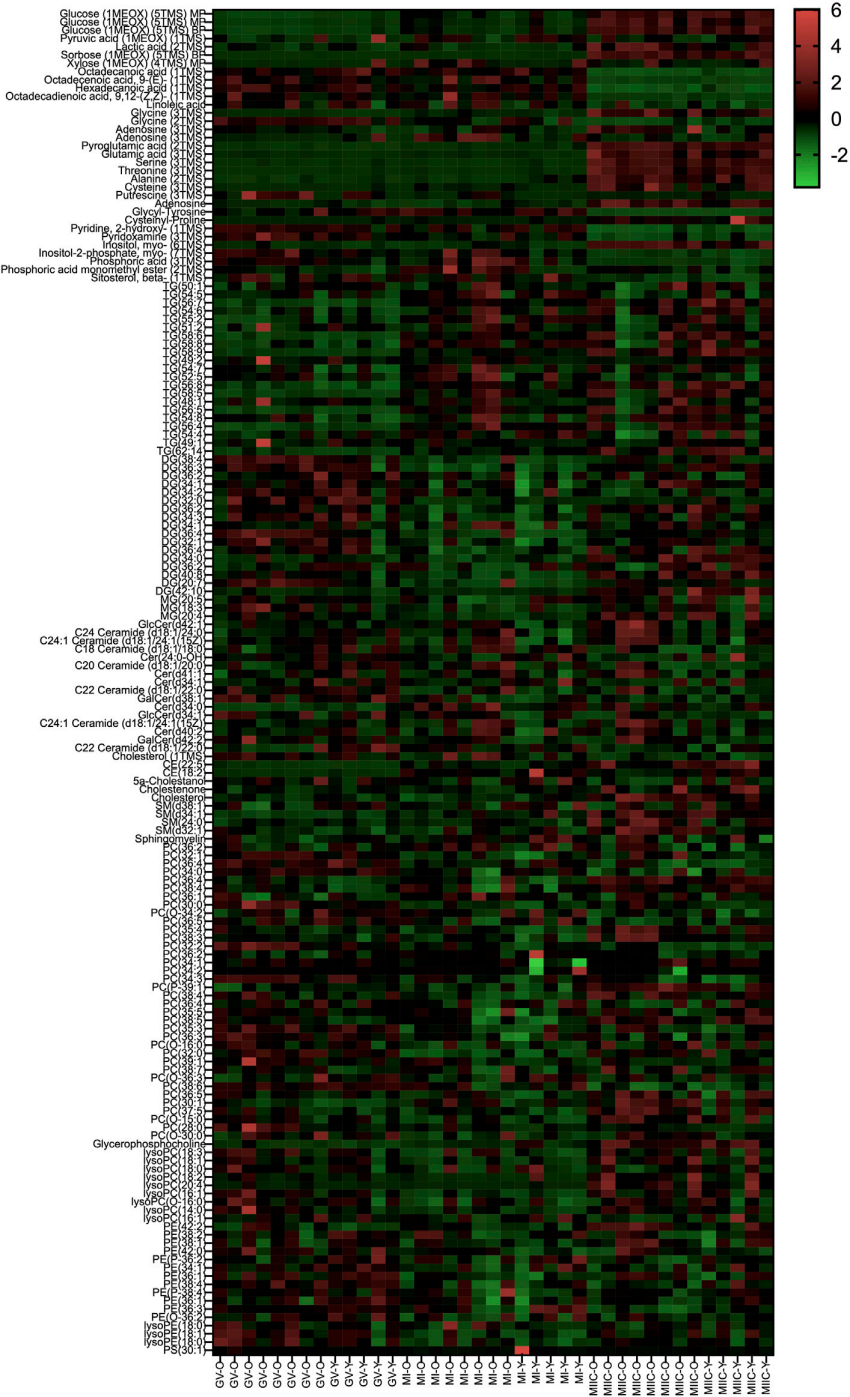
Heat map depicting variability in granulosa cell metabolite abundance at 0 h (GV, germinal vesicle stage) or 24 h (MI, metaphase I stage) after maturation induction, with additional oocytes collected as 24 h and cultured *in vitro* for 18 h (MIIC, metaphase II stage after culture). Green to red indicates greater relative abundance of metabolites. Y and O indicate samples from young or old mares, respectively.

In general, while differences were observed associated with mare age and between maturation stages *in vivo* (GV and MI), the most pronounced differences were observed after exposure to culture medium during the approximately 18 h for completion of maturation (MIIC). PCA plots for oocytes show overlapping of many GV and MI samples, with most separation noted for MIIC (Figure 1A). PCA plots for cumulus cells (Figure 1B) and granulosa cells (Figure 1C) demonstrate some variability with maturation stage *in vitro*; however, the most notable separation is between maturation stages *in vivo* (GV and MI) and after culture (MIIC), with scores for MIIC differentially clustered on the *X*-axis (PC1) for cumulus and granulosa cells. The PCA plots suggest a pronounced effect of *in vitro* culture on metabolite composition of follicular cells, with potential variation between follicle cell types. Heatmaps of metabolite abundance demonstrate variability among samples from individual mares and stages (GV, MI and MIIC) for oocytes (Figure 2), cumulus cells (Figure 3), and granulosa cells (Figure 4).

Some metabolites (glucose, cholesterol, glycine, DG (36:2), lysoPC(16:1), PC(36:2), PC(36:4), C24:1 Ceramide (d18:1/24:1 (15Z)), 2,2,4,4,6,6-Hexamethyl-1,3,5-trithiane, 9-(4-Hydroxybutyl)-N2-Phenylguanine, and adenosine) were detected at multiple molecular peaks, and the peaks were included in Supplementary Tables. Graphical representations of four metabolites of interest (glucose, cholesterol, glycine, and adenosine) are presented using the peak with the highest normalized abundance.

### Effect of maturation stage and cell culture on carbohydrates and derivatives

The abundance of seven carbohydrates and/or their derivatives varied by stage in oocytes, cumulus, or granulosa cells; however, not all metabolites were different for both age groups and in all cell types (Supplementary Tables S1–S3). Abundance of glucose differed by the main effect of stage, but not by mare age or stage-by-age interaction. Glucose abundance in cumulus and granulosa cells was higher after completion of maturation and culture (MIIC) when compared to GV and MI (*p* < 0.001, Figure 2A). Three glucose peaks were documented; the abundance of glucose appeared to have a general, but not significant, increase in oocytes from young mares with maturation and culture. However, for all peaks, glucose abundance in oocytes from old mares tended or had a significant increase from GV to MIIC (Supplementary Table S1). For cumulus and granulosa cells, similar trends in glucose abundance occurred after maturation and culture, with glucose abundance higher at the MIIC stage; however, in general, no differences were observed in glucose abundance between the follicle cells of young and old mares (Supplementary Tables S2, S3).

**FIGURE 5:**
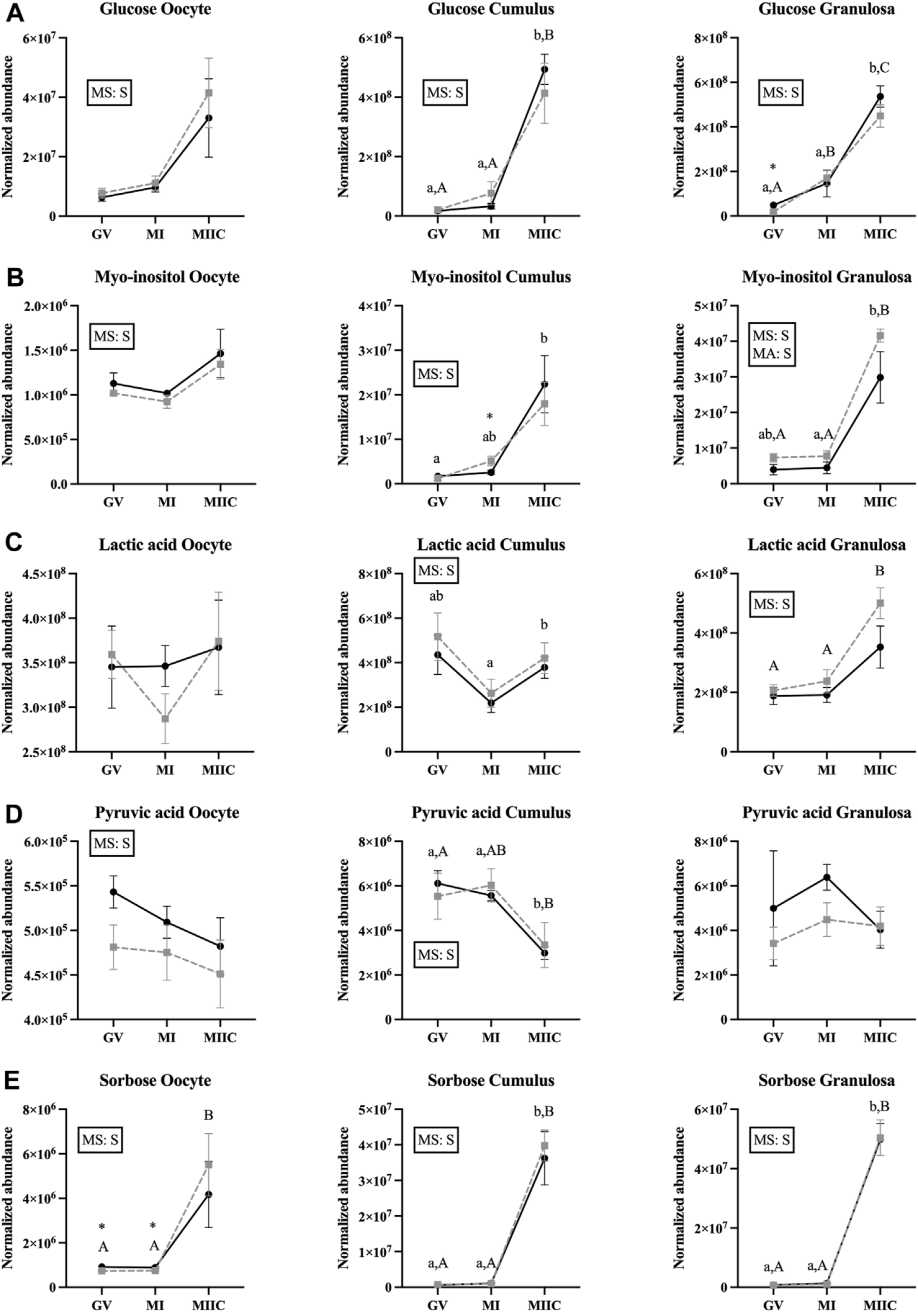
Normalized abundance of selected carbohydrates and their derivatives in oocytes (first column; Young, n = 8 and Old, n = 9), cumulus cells (second column; Young, n = 8 and Old, n = 6), and granulosa cells (third column; Young, n = 5 and Old, n = 9) collected from young mares (solid lines) and old mares (dotted lines) at 0 h (GV, germinal vesicle stage) or 24 h (MI, metaphase I stage) after maturation induction, with additional oocytes collected at 24 h and cultured *in vitro* for 18 h (MIIC, metaphase II stage after culture). Results are presented as mean ± SEM for: **(A)** glucose, **(B)** myo-inositol, **(C)** lactic acid, **(D)** pyruvic acid, **(E)** sorbose. Superscripts indicate differences (*p* < 0.05) among stages (GC, MI and MIIC) for Young (a,b,c) or Old (A,B,C), and between age groups at a specific stage (*). Significant (S, *p* < 0.05) main effects of stage (MS) or mare age (MA) are depicted in each graph. Interactions of stage-by-age were not significant (*p* > 0.1).

The main effect of stage was significant (*p* < 0.05) for abundance of myo-inositol for all cell types, with a significant main effect of age also observed for granulosa cells (Figure 5B). In cumulus cells, myo-inositol abundance was elevated at MIIC when compared to GV for Young (*p* < 0.001, Figure 5B) and tended to be greater in MIIC *versus* GV in Old (Supplementary Table S2). Cumulus cells at MI from Old had a greater abundance of myo-inositol than Young (*p* < 0.05, Figure 5B). Granulosa cell abundance of myo-inositol was elevated at MIIC when compared to GV and MI for Old and in MIIC when compared to MI for Young (*p* < 0.001, Figure 5B); myo-inositol tended (*p* = 0.06) to be elevated in Old *versus* Young at MIIC (Supplementary Table S3).

A significant main effect of stage was observed for the abundance of lactic acid for cumulus and granulosa cells. Lactic acid was greater at MIIC when compared to MI in cumulus cells from Young (*p* < 0.001, Figure 5C), but not Old. Whereas, in granulosa cells, lactic acid was greater (*p* < 0.001) at MIIC when compared to GV and MI from Old, but not Young (Figure 5C).

While not a carbohydrate, pyruvic acid is a key intermediate in carbohydrate metabolism and an important energy source for the oocytes. Abundance of pyruvic acid differed (*p* < 0.05) for the main effect of stage for oocytes and cumulus cells (Figure 5D). Pyruvic acid tended to be greater in oocytes from Young than Old at GV (*p* < 0.1, Supplementary Table S1), although no differences were significant for oocyte stage. However, in cumulus cells, pyruvic acid was less abundant at MIIC when compared to GV and MI for Young and was less in MIIC when compared to GV for Old (*p* < 0.001, Figure 5D).

A significant main effect of stage was observed for the abundance of sorbose for all cell types (Figure 5E). Sorbose was greater in oocytes at MIIC when compared to GV and MI for Old (*p* < 0.001, Figure 5E). Cumulus and granulosa cell abundance of sorbose was elevated in MIIC when compared to GV and MI in both age groups (*p* < 0.001, Figure 5E) suggesting an effect of culture environment on sorbose abundance. Prior to culture at the GV and MI stages, sorbose abundance was significantly greater in oocytes from young than old mares (Figure 5E, Supplementary Table S1).

### Effect of maturation stage and cell culture on lipids and fatty acids

Maturation and/or cell culture resulted in differences in normalized abundance of 24 lipids and fatty acids in oocytes, 97 in cumulus cells, and 83 in granulosa cells; however, not all metabolites were significantly different for both age groups and in all cell types (Supplementary Tables S1–S3).

**FIGURE 6:**
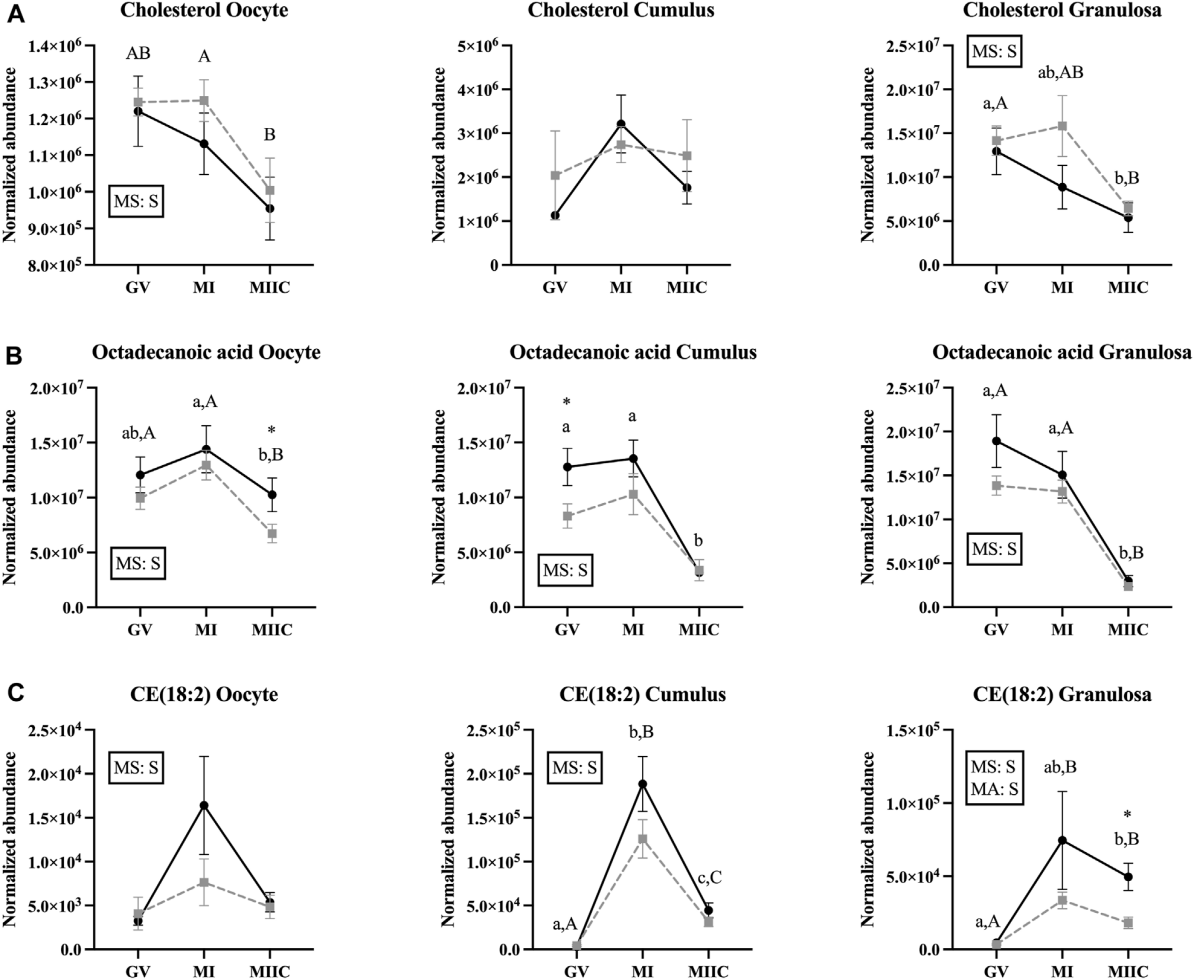
Normalized abundance of selected lipids and fatty acids in oocytes (first column; Young, n = 8 and Old, n = 9), cumulus cells (second column; Young, n = 8 and Old, n = 6), and granulosa cells (third column; Young, n = 5 and Old, n = 9) collected from young mares (solid lines) and old mares (dotted lines) at 0 h (GV, germinal vesicle stage) or 24 h (MI, metaphase I stage) after maturation induction, with additional oocytes collected at 24 h and cultured *in vitro* for 18 h (MIIC, metaphase II stage after culture). Results are presented as mean ± SEM for: **(A)** cholesterol, **(B)** octadecanoic acid, **(C)** CE(18:2). Superscripts indicate differences (*p* < 0.05) among stages (GC, MI and MIIC) for Young (a,b,c) or Old (A,B,C), and between age groups at a specific stage (*). Significant (S, *p* < 0.05) main effects of stage (MS) or mare age (MA) are depicted in each graph. Interactions of stage-by-age were not significant (*p* > 0.1).

A significant main effect of stage was observed for the abundance of cholesterol in oocytes and granulosa cells (Figure 6A), with an apparent decline in cholesterol abundance with advancing stages in oocytes and granulosa cells. In oocyte from Old, abundance of cholesterol was less in MIIC than MI (*p* < 0.001, Figure 6A) and tended to be less than GV (*p* < 0.1, Supplementary Table S1). Cholesterol also tended to be higher in MI *versus* GV for cumulus cells from Young (*p* < 0.1, Supplementary Table S2) and was less in granulosa cells at MIIC when compared to GV for both young and old mares (*p* < 0.001, Figure 6A).

For octadecanoic acid, a common fatty acid that is also known as stearic acid, the main effect of stage differed (*p* < 0.05) for all cell types (Figure 6B). Oocyte abundance of octadecanoic acid was less at MIIC when compared to MI in both age groups (*p* < 0.001, Figure 6B). Cumulus cells from Young had decreased octadecanoic acid at MIIC when compared to GV and MI (*p* < 0.001, Figure 6B); while in Old, the metabolite tended be to less in MIIC than GV (*p* < 0.1, Supplementary Table S2). In granulosa cells from both age groups, octadecanoic acid was less at MIIC when compared to GV and MI (*p* < 0.001, Figure 6B). A second peak of octadecanoic acid was observed, with a similar decline in abundance at MIIC than earlier stages (Supplementary Table S2).

The main effect of stage was significant in all cell types for cholesteryl linoleate, CE (18:2), a cholesterol ester, with a significant effect of mare age for granulosa cells (Figure 6C). In general, the metabolite appeared to increase with maturation *in vivo* and then decline after culture (MIIC). In cumulus cells, CE(18:2) increased from GV to MI for young and old mares, then declined from MI to MIIC in both age groups (*p* < 0.001, Figure 6C). In granulosa cells, CE(18:2) increased from GV to MIIC in Young and from GV to both MI and MIIC in Old; the abundance of CE(18:2) was greater in granulosa cells from Young than Old at MIIC (Figure 6D). The main effect of stage differed (*p* < 0.001) for another cholesteryl ester, CE(22:5), which increased in abundance from GV to MIIC in the cumulus and granulosa cells of both age groups (Supplementary Tables S2, S3).

Eight diacylglycerol (DG) compounds were altered in abundance in oocytes, with the main effect of stage significant (*p* ≤ 0.02) in all but one DG (*p* = 0.09) with most differences noted as a greater abundance after culture at MIIC *versus* earlier stages (Supplementary Table S1). In cumulus cells, the main effect of stage was significant (*p* ≤ 0.03) for 13 DGs and the interaction of stage-by-age for DG(20:7) at *p* = 0.01 (Supplementary Table S2). The abundance of specific significant DGs varied in young and old mares and in increasing or decreasing with stage, and the main effect of age did not differ. However, DG(36:4) increased from GV and MI to MIIC in both age groups. Similar to cumulus cells, 13 DGs differed (*p* ≤ 0.02) by the main effect of stage in granulosa cells (Supplementary Table S3). However, four DGs in granulosa cells differed (*p* < 0.05) in the main effect of age (Supplementary Table S3). Similar variation was observed in normalized abundance patterns and, like cumulus cells, DG(36:4) increased from MI to MIIC in both age groups; in addition, the interaction of stage-by-age differed (*p* = 0.05) for this DG (Supplementary Table S3).

Monoacylglycerides (MG) did not differ in oocytes for stage or mare age. Only MG (18:3) differed in cumulus cells with a main effect of stage (*p* = 0.01) and tendency for interaction of stage-by-age (*p* = 0.10); MG(18:3) decreased in cumulus cells from GV to MIIC in Young and GV to MI in Old (Supplementary Table S2). In granulosa cells, a main effect of stage was noted for 2 MGs (Supplementary Table S3).

Numerous phospholipids differed with stage, including phosphatidylcholines (PC), lysophosphatidylcholines (LysoPC), phosphatidylethanolamine (PE), lysophosphatidylethanolamine (LysoPE), phosphatidylserines (PS), and sphingomyelin (SM) (Supplementary Tables S1–S3). The main effect of stage was similar for all PCs in oocytes but differed (*p* ≤ 0.04) for 27 PCs in cumulus cells. No differences were observed for the main effect of age, and a significant stage-by-age interaction was only observed for PC(O-16:0) (Supplementary Table S2). Of the 27 PCs, 24 increased or tended to increase from GV to MI or across all three stages. This was observed more consistently for young mares. In granulosa cells, the main effect of stage differed (*p* ≤ 0.04) for 26 PCs, with a significant (*p* = 0.02) interaction of stage-by-age for PC(34:1). The main effect of age was significant (*p* < 0.05) for six PCs (Supplementary Table S3). In contrast to cumulus cells, stage differences were observed more often for Old than Young. Of the 26 PCs, 13 decreased or tended to decrease, while eight increased or tended to increase, and four decreased from GV to MI then increased at MIIC.

For lysoPCs in all cell types, lysoPC(16:1) and lysoPC(20:4) differed by stage. In oocytes, four lysoPCs differed (*p* ≤ 0.03) by the main effect of stage, with an interaction (*p* = 0.05) of stage-by-age for lysoPC(O-16:0) (Supplementary Table S1). Seven lysoPCs differed (*p* ≤ 0.3) by the main effect of stage, with an overall trend for most lysoPCs to have a greater abundance in MIIC than earlier stages. Significant differences among stages were only observed for Young. In granulosa cells, only three lysoPCs differed (*p* ≤ 0.01) by a main effect of stage; however, significant differences among stages were observed only for Old. No lysoPCs differed by the main effect of age.

Phosphatidylethanolamines (PEs) are phospholipids typically associated with membranes. In oocytes, like PC, no PEs varied by the main effect of stage. Seven PEs differed by stage in cumulus cells, with significant differences between GV and MI or MIIC mostly observed for Young. No main effect of age was observed for cumulus cell PEs. Five PEs in granulosa cells differed (*p* ≤ 0.03) by the main effect of stage, with PE (36:3) differing by age (*p* < 0.03) and PE (36:1) demonstrating a stage-by-age interaction (*p* = 0.05, Supplementary Table S3).

Differences for the main effect of stage were only observed in cumulus and granulosa cells for lysoPE. In cumulus and granulosa cells, lysoPE (18:1) differed with stage; in granulosa cells, the compound was greater in Old at GV than MI (Supplementary Table S3). An additional lysoPE(18:0) also differed by stage in granulosa cells. No main effects of age or interactions were observed.

Only PS(30:1) differed by stage in cumulus cells; no other differences were noted for phosphatidylserines. Sphingomyelin (SM) abundance did not differ in oocytes. In cumulus cells, three SMs differed (*p* ≤ 0.01) by stage, with two SMs, SM(d32:1) and SM(d34:1), significantly increasing in abundance from GV to MI or MIIC in Young and Old (Supplementary Table S2). In granulosa cells, three SM differed by a main effect of stage, including SM(d32:1) and SM(d34:1), with SM(d32:1) also differing (*p* < 0.05) with a main effect of age (Supplementary Table S3). Interactions of stage-by-age tended to differ for SM(24:0) and SM(d32:1) at *p* = 0.08 and *p* = 0.05, respectively. Abundance of all three SMs in granulosa cells was significantly greater for MI or MIIC than GV, with only SM(d34:1) demonstrating significant differences among stages (Supplementary Table S3).

Triacylglycerols (TG) demonstrated differences associated with stage in all cycle types, with more differences observed in the somatic cells. In oocytes, only TG(49:1) differed (*p* = 0.05) by stage, with the TG tending (*p* < 0.10) to decline for Young and Old from MI to MIIC. In cumulus cells, 18 TGs differed by stage, and 5 TGs also differed (*p* < 0.05) in the main effect of age (Supplementary Table S2). Three tended to decrease across the stages and were significant more often for young mares while 12 increased or tended to increase across maturation stages and often in both age groups. The remaining three had an overall effect of stage but no difference within age groups between stages (Supplementary Table S2). In granulosa cells, 13 TGs differed significantly by stage, and TG(54:7) also demonstrated a main effect of age (*p* < 0.05). However, in contrast to cumulus cells, all TGs increased or tended to increase from GV to MI and, in general, remained elevated in MIIC in Old, while 10 TG increased or tended to increase in Young.

Another category of lipids and fatty acids that had were affected by maturation stage were ceramides and related molecules. No differences in abundance of ceramides were observed in oocytes. However, ten ceramides or related compounds significantly differed in cumulus cells (Table 1), and six differed in granulosa cells (Table 2). In cumulus, six of the ten compounds increased or tended to increase as maturation progressed, with some variation in mare age and stage as to significance. One ceramide, Cer(d34:1), increased significantly from GV to MI then declined at MIIC in both age groups (Table 1). Variation was observed in granulosa cells, as to whether ceramides increased or decreased and as to the effected age groups (Table 2).

**TABLE 1:** Relative abundance of ceramides and related molecules in cumulus cells. Cumulus cells were collected from young mares (Yg, n = 8) and old mares (Old, n = 6) at 0 h (GV, germinal vesicle stage) or 24 h (MI, metaphase I stage) after maturation induction, with additional oocytes collected as 24 h and cultured *in vitro* for an additional 18 h (MIIC, metaphase II stage after culture). Results are presented as mean ± SEM and correction factor of 10x (CF). *p*-values for the main effect of stage (MS) and the interaction (INT) of stage-by-age are included in the table; the main effect of age was not significant (*p* > 0.05) for any of the metabolites. Different superscripts within a row represent differences at ^a,b,c^ *p* < 0.05 or ^d,e,f^ *p* < 0.1. Superscripts within a column for the same metabolite represent differences between Yg and Old at ^+^ *p* < 0.1.

Ceramides and related molecules	Age	GV	MI	MIIC	CF	MS	INT
C18 Ceramide (d18:1/18:0)	Yg	5.47 ± 0.90^d^	11.87 ± 1.83^e^	7.94 ± 1.80^de^	10^3^	0.01	0.46
	Old	4.04 ± 0.51	9.73 ± 2.78	9.79 ± 2.31			
C20 Ceramide (d18:1/20:0)	Yg	3.37 ± 0.62^a^	6.07 ± 1.07^b^	4.65 ± 1.09^ab^	10^3^	0.02	0.41
	Old	3.00 ± 0.90	5.23 ± 1.36	6.10 ± 1.20			
C22 Ceramide (d18:1/22:0)	Yg	5.33 ± 0.82	5.25 ± 1.10	7.87 ± 1.48	10^3^	0.01	0.62
	Old	3.58 ± 0.62^d^	5.53 ± 1.41^de^	7.44 ± 1.16^e^			
C24:1 Ceramide (d18:1/24:1 (15Z))	Yg	4.85 ± 0.80^a^	9.52 ± 2.64^ab^	12.22 ± 1.78^b^	10^3^	<0.01	0.65
	Old	5.17 ± 2.62	8.33 ± 1.90	15.06 ± 3.30			
C24:1 Ceramide (d18:1/24:1 (15Z))	Yg	3.73 ± 0.88	2.34 ± 0.71	2.49 ± 0.19	10^2^	0.04	0.41
	Old	3.61 ± 0.60^d^	1.31 ± 0.33^e^	3.31 ± 1.16^de^			
Cer(24:0-OH)	Yg	4.55 ± 0.70^d^	2.40 ± 0.98^de^	1.73 ± 0.34^e^	10^5^	<0.01	0.81
	Old	3.78 ± 0.78	1.38 ± 0.29	1.58 ± 0.60			
Cer(d34:1)	Yg	5.69 ± 1.26^a^	36.07 ± 3.83^b^	16.48 ± 2.36^c^	10^3^	<0.001	0.29
	Old	4.51 ± 1.33^a^	30.68 ± 1.61^b^	21.43 ± 6.30^ab^			
Cer(d34:0)	Yg	2.78 ± 0.32^a^	2.74 ± 0.38^a^	1.52 ± 0.11^b,+^	10^3^	0.01	0.12
	Old	2.67 ± 0.40	1.92 ± 0.24	1.98 ± 0.26^+^			
Cer(d40:2)	Yg	1.55 ± 0.23^a^	2.84 ± 0.34^b^	2.37 ± 0.20^b^	10^3^	0.01	0.08
	Old	1.77 ± 0.31	2.09 ± 0.30	3.10 ± 0.66			
GlcCer(d34:1)	Yg	8.15 ± 1.73	6.40 ± 1.14	4.69 ± 0.64	10^3^	0.02	1.00
	Old	9.22 ± 2.00	7.59 ± 1.60	5.66 ± 0.96			

Cer, ceramide; GlcCer, glucosylceramide.

**TABLE 2:** Relative abundance of ceramides and related molecules in granulosa cells. Granulosa cells were collected from young mares (Yg, n = 5) and old mares (Old, n = 9) at 0 h (GV, germinal vesicle stage) or 24 h (MI, metaphase I stage) after maturation induction, with additional oocytes collected as 24 h and cultured *in vitro* for an additional 18 h (MIIC, metaphase II stage after culture). Results are presented as mean ± SEM and correction factor of 10x (CF). *p*-values for the main effect of stage (MS) and the interaction (INT) of stage-by-age are included in the table; the main effect of age was not significant (*p* > 0.05) for any of the metabolites. Different superscripts within a row represent differences at ^a,b,c^ *p* < 0.05 or ^d,e,f^ *p* < 0.1. Superscripts within a column for the same metabolite represent differences between Yg and Old at ^*^ *p* < 0.05 or ^+^ *p* < 0.1.

Ceramides and related molecules	Age	GV	MI	MIIC	CF	MS	INT
C18 Ceramide (d18:1/18:0)	Yg	10.81 ± 1.63	9.68 ± 1.59	6.47 ± 0.56	10^3^	0.04	0.32
	Old	8.23 ± 1.10	10.61 ± 1.26	7.21 ± 1.31			
Cer(d34:0)	Yg	1.34 ± 0.27	2.96 ± 0.48	2.61 ± 0.59	10^3^	<0.001	0.40
	Old	1.19 ± 0.11^a^	3.99 ± 0.70^b^	2.99 ± 0.50^c^			
Cer(d41:1)	Yg	2.58 ± 0.48^ab^	1.54 ± 0.25^a,+^	2.11 ± 0.35^b^	10^3^	0.29	0.90
	Old	2.02 ± 0.23	2.33 ± 0.29^+^	2.78 ± 0.40			
GalCer(d38:1)	Yg	15.44 ± 2.16^a,d^	8.80 ± 1.75^ab,e^	8.16 ± 1.09^b^	10^3^	<0.001	0.73
	Old	14.05 ± 1.68^a^	9.83 ± 1.57	7.67 ± 0.85^b^			
GalCer(d42:2)	Yg	11.24 ± 1.95^a^	5.06 ± 1.10^b,*^	5.59 ± 0.82^ab^	10^3^	0.03	0.43
	Old	13.89 ± 3.05	10.51 ± 1.54^*^	12.85 ± 2.67			
GlcCer(d34:1)	Yg	9.78 ± 2.12^a^	6.04 ± 1.83^b^	4.76 ± 0.60^ab^	10^3^	<0.001	0.66
	Old	9.88 ± 1.70^d^	8.01 ± 0.99^de^	6.21 ± 1.22^e^			

Cer, ceramide; GalCer, galactosylceramide; GlcCer, glucosylceramide.

### Effect of maturation stage and cell culture on amino acids and derivatives

Stage and/or culture resulted in differences in normalized abundance of amino acid or derivatives in oocytes (n = 9), cumulus cells (n = 12), and granulosa cells (n = 12) (Tables 3–5). In oocytes, most significant changes with time occurred at MIIC, with all five amino acids that differed (*p* < 0.05) in abundance by stage occurring in oocytes from old mares. Significant main effects of age were observed for glutamic acid, with abundance at all stages tending (*p* < 0.1) to be greater in Old than Young, and for glycine (3TMS), with abundance greater in old than young mares. Interactions of stage-by-age tended to be significant for threonine (*p* = 0.1) and glycine (3TMS) (*p* = 0.12), with greater (*p* < 0.05) abundance of glycine in Old than Young at MI (Table 3).

**TABLE 3:** Relative abundance of amino acids and derivatives in oocytes. Oocytes were collected from young mares (Yg, n = 8) and old mares (Old, n = 9) at 0 h (GV, germinal vesicle stage) or 24 h (MI, metaphase I stage) after maturation induction, with additional oocytes collected as 24 h and cultured *in vitro* for an additional 18 h (MIIC, metaphase II stage after culture). Results are presented as mean ± SEM and correction factor of 10^x^ (CF). *p*-values for the main effect of stage (MS) and the interaction (INT) of stage-by-age are included in the table; the main effect of age was significant (*p* < 0.05) for metabolites with bolded Yg/Old within a grey box in the second column. Different superscripts across time represent differences at ^a,b,c^ *p* < 0.05 or ^d,e^ *p* < 0.1. Superscripts within a column for the same metabolite represent differences between Yg and Old at ^*^ *p* < 0.05 or ^+^ *p* < 0.1.

Amino acids and derivatives	Age	GV	MI	MIIC	CF	MS	INT
α-ketobutyrate	Yg	2.14 ± 0.38^d^	3.52 ± 0.27^e,*^	3.87 ± 0.81^f^	10^3^	0.02	0.41
	Old	2.00 ± 0.26	2.23 ± 0.27^*^	3.64 ± 0.73			
Alanine	Yg	11.70 ± 0.79^+^	11.16 ± 0.69^*^	13.54 ± 1.16	10^6^	<0.001	0.17
	Old	9.81 ± 0.68^ab,+^	8.13 ± 0.53b^a,*^	12.82 ± 0.98^b^			
Cysteine	Yg	2.87 ± 0.35	3.02 ± 0.69	1.85 ± 0.21	10^5^	<0.001	0.64
	Old	2.18 ± 0.31^a^	2.98 ± 0.48^a^	1.44 ± 0.26^b^			
ɛ-polylysine	Yg	0.66 ± 0.42	3.65 ± 2.21	0.08 ± 0.03	10^4^	0.03	0.64
	Old	3.76 ± 2.36	5.08 ± 2.82	0.52 ± 0.44			
Glutamic acid	**Yg**	6.84 ± 0.61^+^	7.66 ± 1.30^+^	9.96 ± 2.36^+^	10^5^	0.02	0.27
	**Old**	14.01 ± 3.48^+^	13.25 ± 2.23^+^	20.37 ± 4.44^+^			
Glycine (3TMS)	**Yg**	28.81 ± 0.54	27.09 ± 0.67^*^	30.16 ± 1.66	10^6^	0.01	0.12
	**Old**	28.53 ± 0.76^d^	29.40 ± 0.57^de,*^	35.48 ± 2.49^e^			
Glycyl-tyrosine	Yg	31.62 ± 19.04	63.65 ± 25.08	1.42 ± 0.70	10^4^	<0.001	0.88
	Old	57.37 ± 20.46^ab^	92.38 ± 23.49^a^	14.25 ± 12.85^b^			
Pyroglutamic acid	Yg	5.10 ± 0.58	5.95 ± 1.20	9.56 ± 3.09	10^6^	<0.001	0.16
	Old	6.06 ± 1.09^a^	7.42 ± 0.70^a^	15.80 ± 3.03^b^			
Threonine	Yg	5.51 ± 0.47	6.51 ± 1.28	8.53 ± 2.28	10^5^	<0.001	0.10
	Old	4.66 ± 0.69^ab,d^	4.97 ± 0.51^a^	12.03 ± 2.17^b,e^			

**TABLE 4:** Relative abundance of amino acids and derivatives in cumulus cells. Cumulus cells were collected from young mares (Yg, n = 8) and old mares (Old, n = 6) at 0 h (GV, germinal vesicle stage) or 24 h (MI, metaphase I stage) after maturation induction, with additional oocytes collected as 24 h and cultured *in vitro* for an additional 18 h (MIIC, metaphase II stage after culture). Results are presented as mean ± SEM and correction factor of 10x (CF). *p*-values for the main effect of stage (MS) and the interaction (INT) of stage-by-age are included in the table; the main effect of age was not significant (*p* > 0.05) for any of the metabolites. Different superscripts within a row represent differences at ^a,b,c^ *p* < 0.05 or ^d,e,f^ *p* < 0.1. Superscripts within a column for the same metabolite represent differences between Yg and Old at ^*^ *p* < 0.05 or ^+^ *p* < 0.1.

Amino acids and derivatives	Age	GV	MI	MIIC	CF	MS	INT
α-ketobutyrate	Yg	1.57 ± 0.18^a^	22.20 ± 3.37^b^	34.26 ± 6.20^b^	10^3^	<0.001	0.24
	Old	1.95 ± 0.23^a^	18.60 ± 3.39^b^	21.17 ± 3.10^b^			
Alanine	Yg	12.11 ± 0.93^a^	8.09 ± 1.36^a^	31.54 ± 3.52^b,+^	10^6^	<0.001	0.04
	Old	11.59 ± 2.27^ab^	8.33 ± 1.50^a^	21.69 ± 2.27^b,+^			
Cysteine	Yg	1.75 ± 0.27^ab,d^	1.43 ± 0.16^a^	5.04 ± 1.03^b,e^	10^5^	0.01	0.45
	Old	1.29 ± 0.11	1.55 ± 0.38	7.36 ± 3.35			
Cysteinyl-Proline	Yg	5.38 ± 2.38	2.65 ± 1.24	140.09 ± 55.80	10^2^	0.01	0.31
	Old	6.78 ± 2.74^a^	3.42 ± 1.15^a^	70.54 ± 18.28^b^			
ɛ-polylysine	Yg	54.52 ± 26.91^de^	103.39 ± 29.28^d^	9.41 ± 4.83^e^	10^3^	<0.01	0.86
	Old	38.11 ± 23.96	89.00 ± 21.74	15.32 ± 13.03			
Glutamic acid	Yg	5.22 ± 0.44^a,*^	7.36 ± 0.93^a,*^	85.24 ± 9.21^b^	10^5^	<0.001	0.76
	Old	11.36 ± 3.08^a,*^	15.20 ± 3.27^b,d,*^	85.80 ± 21.33^b,e^			
Glycine (3TMS)	Yg	2.84 ± 0.12^a^	2.35 ± 0.16^a,+^	7.71 ± 0.54^b^	10^7^	<0.001	0.07
	Old	2.81 ± 0.10^a^	3.12 ± 0.42^a,+^	6.39 ± 0.79^b^			
Glycine (2TMS)	Yg	6.76 ± 0.54^a,*^	6.69 ± 0.32^a^	2.70 ± 0.42^b^	10^7^	<0.001	0.20
	Old	4.97 ± 0.62^ab,d,*^	5.25 ± 0.84^a^	2.61 ± 0.76^b,e^			
Glycyl-tyrosine	Yg	71.47 ± 26.08^ab^	98.31 ± 20.53^a^	4.44 ± 1.90^b^	10^4^	<0.001	0.47
	Old	45.70 ± 21.6^a^	116.89 ± 15.65^b^	25.70 ± 23.36^ab^			
Pyroglutamic acid	Yg	5.32 ± 1.67^a^	5.49 ± 0.70^a^	91.45 ± 4.37^b^	10^6^	<0.001	0.18
	Old	5.49 ± 0.72^a^	18.53 ± 9.52^a^	81.82 ± 16.20^b^			
Serine	Yg	2.92 ± 0.86^a^	1.09 ± 0.37^a^	7.90 ± 0.53^b^	10^6^	0.01	0.32
	Old	3.02 ± 1.31	4.16 ± 3.30	6.93 ± 1.21			
Threonine	Yg	4.82 ± 1.39^a^	3.05 ± 0.35^a^	50.94 ± 4.69^b^	10^5^	<0.001	0.17
	Old	3.60 ± 0.65^a^	7.80 ± 4.07^a^	40.51 ± 8.34^b^			

**TABLE 5:** Relative abundance of amino acids and derivatives in granulosa cells. Granulosa cells were collected from young mares (Yg, n = 5) and old mares (Old, n = 9) at 0 h (GV, germinal vesicle stage) or 24 h (MI, metaphase I stage) after maturation induction, with additional oocytes collected as 24 h and cultured *in vitro* for an additional 18 h (MIIC, metaphase II stage after culture). Results are presented as mean ± SEM and correction factor of 10x (CF). *p*-values for the main effect of stage (MS) and the interaction (INT) of stage-by-age are included in the table; the main effect of age was significant (*p* < 0.05) for metabolite with bolded Yg/Old within a grey box in the second column. Different superscripts within a row represent differences at ^a,b,c^ *p* < 0.05 or ^d,e,f^ *p* < 0.1. Superscripts within a column for the same metabolite represent differences between Yg and Old at ^*^ *p* < 0.05 or ^+^ *p* < 0.1.

Amino acids and derivatives	Age	GV	MI	MIIC	CF	MS	INT
α-ketobutyrate	Yg	1.55 ± 0.45^a^	13.46 ± 5.83^ab^	13.51 ± 2.67^b^	10^3^	<0.001	0.27
	Old	2.17 ± 0.58^a,d^	6.50 ± 1.31^b^	8.92 ± 2.29^ab,e^			
Alanine	Yg	5.77 ± 0.94^a^	13.78 ± 3.71^a,*^	39.61 ± 7.14^b^	10^6^	<0.001	0.22
	Old	4.76 ± 0.62^a^	5.04 ± 0.48^a,*^	41.94 ± 3.80^b^			
Cysteine	Yg	3.20 ± 2.13	2.49 ± 1.32^+^	45.18 ± 17.81	10^5^	<0.001	0.27
	Old	1.90 ± 0.38^a^	8.59 ± 2.28^b,+^	74.95 ± 13.23^c^			
Cysteinyl-proline	Yg	2.04 ± 1.47	4.01 ± 1.48	52.69 ± 27.04	10^2^	<0.001	0.29
	Old	0.33 ± 0.09^a,d^	2.98 ± 0.80^a,e^	28.21 ± 2.83^b^			
ɛ-polylysine	Yg	31.74 ± 6.12^a^	49.89 ± 8.37^a^	3.43 ± 0.59^b^	10^3^	<0.001	0.87
	Old	26.21 ± 9.35^ab^	53.72 ± 12.02^a^	4.84 ± 0.83^b^			
Glutamic acid	Yg	0.59 ± 0.17^d,*^	0.80 ± 0.41^d,+^	14.79 ± 2.03^e^	10^6^	<0.001	0.21
	Old	1.50 ± 0.21^a,*^	1.56 ± 0.23^a,+^	23.04 ± 3.52^b^			
Glycine (3TMS)	**Yg**	2.00 ± 0.14^a,*^	2.76 ± 0.45^a,+^	10.87 ± 1.60^b^	10^7^	<0.001	0.19
	**Old**	2.84 ± 0.10^a,d,*^	3.97 ± 0.44^a,e,+^	15.89 ± 1.97^b^			
Glycine (2TMS)	Yg	5.96 ± 0.77^a^	3.60 ± 1.14^ab^	1.87 ± 0.26^b^	10^7^	<0.001	0.98
	Old	5.65 ± 0.28^a^	3.22 ± 0.53^b,d^	1.58 ± 0.23^b,e^			
Glycyl-tyrosine	Yg	42.50 ± 5.53^a^	52.41 ± 5.37^b^	2.76 ± 0.45^c^	10^4^	<0.001	0.51
	Old	30.35 ± 7.77^a^	41.99 ± 5.86^a^	3.03 ± 4.63^b^			
Pyroglutamic acid	Yg	0.56 ± 0.15^a,d^	1.67 ± 0.38^a,e^	10.72 ± 0.53^b^	10^7^	<0.001	0.10
	Old	0.95 ± 0.14^a^	1.20 ± 0.11^a^	12.57 ± 0.77^b^			
Serine	Yg	0.74 ± 0.21^a^	0.73 ± 0.24^a^	9.93 ± 0.77^b^	10^6^	<0.001	0.22
	Old	0.63 ± 0.10^a^	0.43 ± 0.07^a^	11.34 ± 0.79^b^			
Threonine	Yg	3.44 ± 1.17^a^	3.92 ± 1.75^a^	68.39 ± 6.89^b^	10^5^	<0.001	0.52
	Old	1.97 ± 0.17^a^	2.87 ± 0.56^a^	74.87 ± 7.20^b^			

**FIGURE 7:**
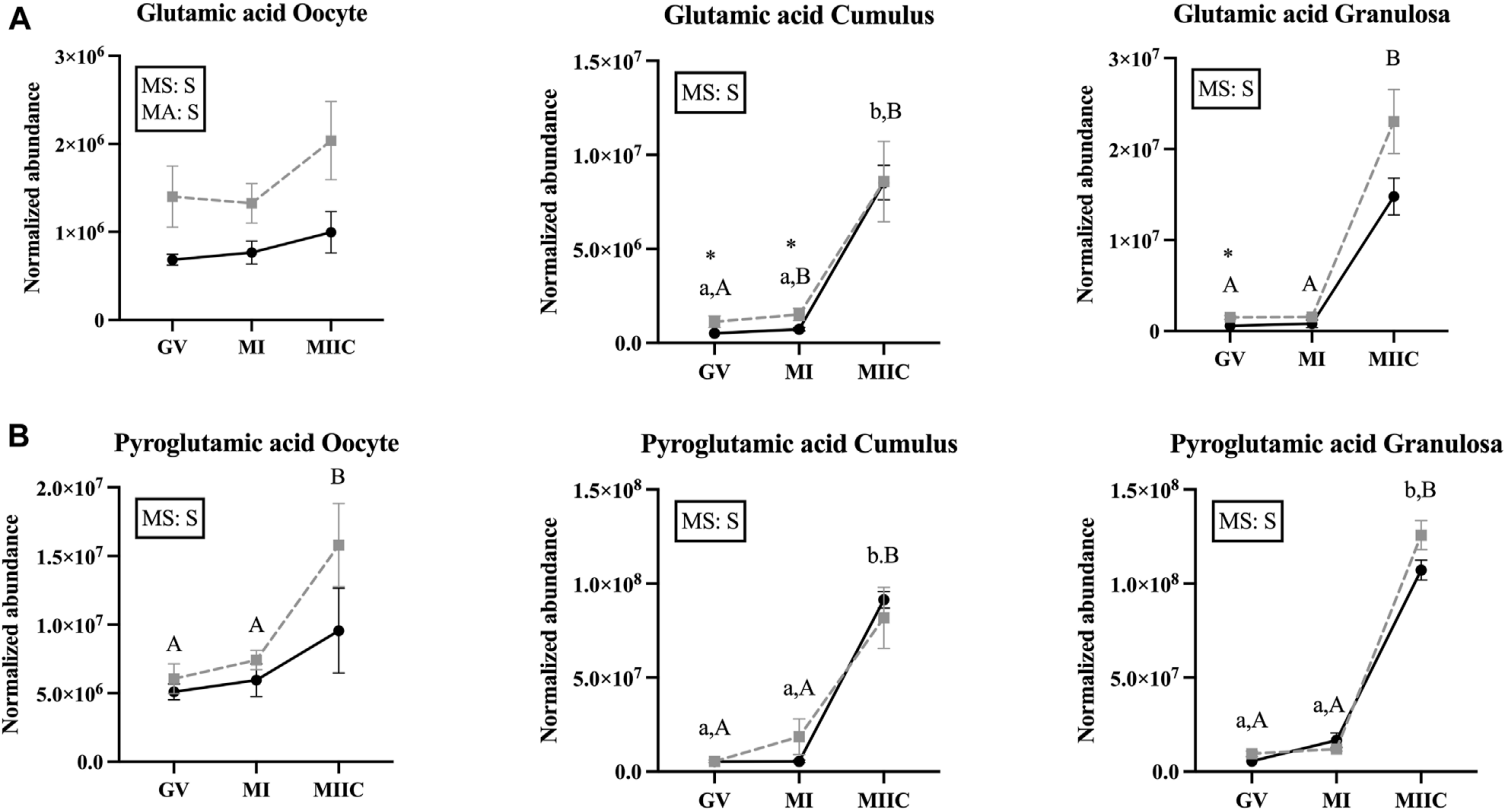
Normalized abundance of selected amino acids in oocytes (first column; Young, n = 8 and Old, n = 9), cumulus cells (second column; Young, n = 8 and Old, n = 6), and granulosa cells (third column; Young, n = 5 and Old, n = 9) collected from young mares (solid lines) and old mares (dotted lines) at 0 h (GV, germinal vesicle stage) or 24 h (MI, metaphase I stage) after maturation induction, with additional oocytes collected at 24 h and cultured *in vitro* for 18 h (MIIC, metaphase II stage after culture). Results are presented as mean ± SEM for: **(A)** glutamic acid, **(B)** pyroglutamic acid. Superscripts indicate differences (*p* < 0.05) among stages (GC, MI and MIIC) for Young (a,b,c) or Old (A,B,C), and between age groups at a specific stage (*). Significant (S, *p* < 0.05) main effects of stage (MS) or mare age (MA) are depicted in each graph. Interactions of stage-by-age were not significant (*p* > 0.1).

In cumulus cells, 12 amino acids or derivatives were altered by stage, but no main effects of age were observed (Table 4). Abundance of alanine had a stage-by-age interaction (*p* = 0.04) with significant increases from MI to MIIC for both age groups, although a significant difference was only observed for Young between GV and MIIC (Table 4). Abundance of alanine tended to be higher (*p* < 0.1) at MIIC. Glycine (3TMS) tended (*p* < 0.07) to have a stage-by-age interaction, with abundance greater (*p* < 0.05) in MIIC than in GV or MI for both age groups (Table 4). Of the other amino acids that increased or tended to increase with maturation stage, α-ketobutyrate increased in both age groups from GV and MI to MIIC (*p* < 0.001, Table 4). Cysteine increased from MI to MIIC in Young, while cysteinyl-proline increased from GV and MI to MIIC in Old (Table 4). Glutamic acid was higher in Old than Young at GV and MI stages but did not have an overall main effect of age (Figure 7A). Additionally, glutamic acid increased from GV and MI to MIIC in Young and from GV to MI and MIIC in Old. Pyroglutamic acid (Figure 7B) and threonine increased in MIIC when compared to GV and MI in both age groups (Table 4), while serine increased from GV and MI to MIIC only in young mares. Glycine was interesting in that one derivative (3TMS) increased from GV and MI to MIIC in both age groups, but the other derivative (2TMS) decreased from GV and MI to MIIC in Young and MI to MIIC in Old (Table 4). The only other amino acid to decrease with stage was glycly-tyrosine which decreased from MI to MIIC in Young and both GV and MI to MIIC in Old (Table 4).

In granulosa cells, 12 amino acids or derivatives were significantly altered by stage. A main effect of age was observed for glycine (3TMS) (*p* = 0.05), with greater abundance in Old and abundance at GV and MI higher (*p* < 0.05 and *p* < 0.1, respectively) for Old than Young. Many of the amino acids significantly altered with stage were greater at MIIC, although some amino acids varied from GV to MI or decreased at MIIC (Table 5). Of the amino acids that increased with stage, α-ketobutyrate increased in young mares from GV to MIIC and from GV to MI in old mares but the difference was not maintained at MIIC (*p* < 0.001, Table 5). Alanine was greater (*p* < 0.05) in MIIC when compared to GV and MI (Table 5). Cysteine increased from GV to MI to MIIC, while cysteinyl-proline increased from GV and MI to MIIC only in old mares (Table 5). Glutamic acid was higher (*p* < 0.05) at GV and tended (*p* < 0.1) to be higher at MI for Old than Young, although no main effect of age was observed (Figure 7A). Additionally, glutamic acid was greater (*p* < 0.05) from GV and MI to MIIC in Old and only tended (*p* < 0.1) to be greater in Young (Table 5). Pyroglutamic acid (Figure 7B), serine, and threonine increased in abundance at MIIC when compared to GV and MI in both age groups (Table 5). Similar to observed in cumulus cells, one derivative of glycine (3TMS) increased from GV and MI to MIIC in both age groups, but the other derivative (2TMS) decreased from GV to MIIC in Young and GV to MI and MIIC in Old (Table 5). Glycyl-tyrosine and, ɛ-polylysine were less (*p* < 0.05) abundant in MIIC relative to MI (Table 5).

### Effect of maturation stage and cell culture on other miscellaneous metabolites

**FIGURE 8:**
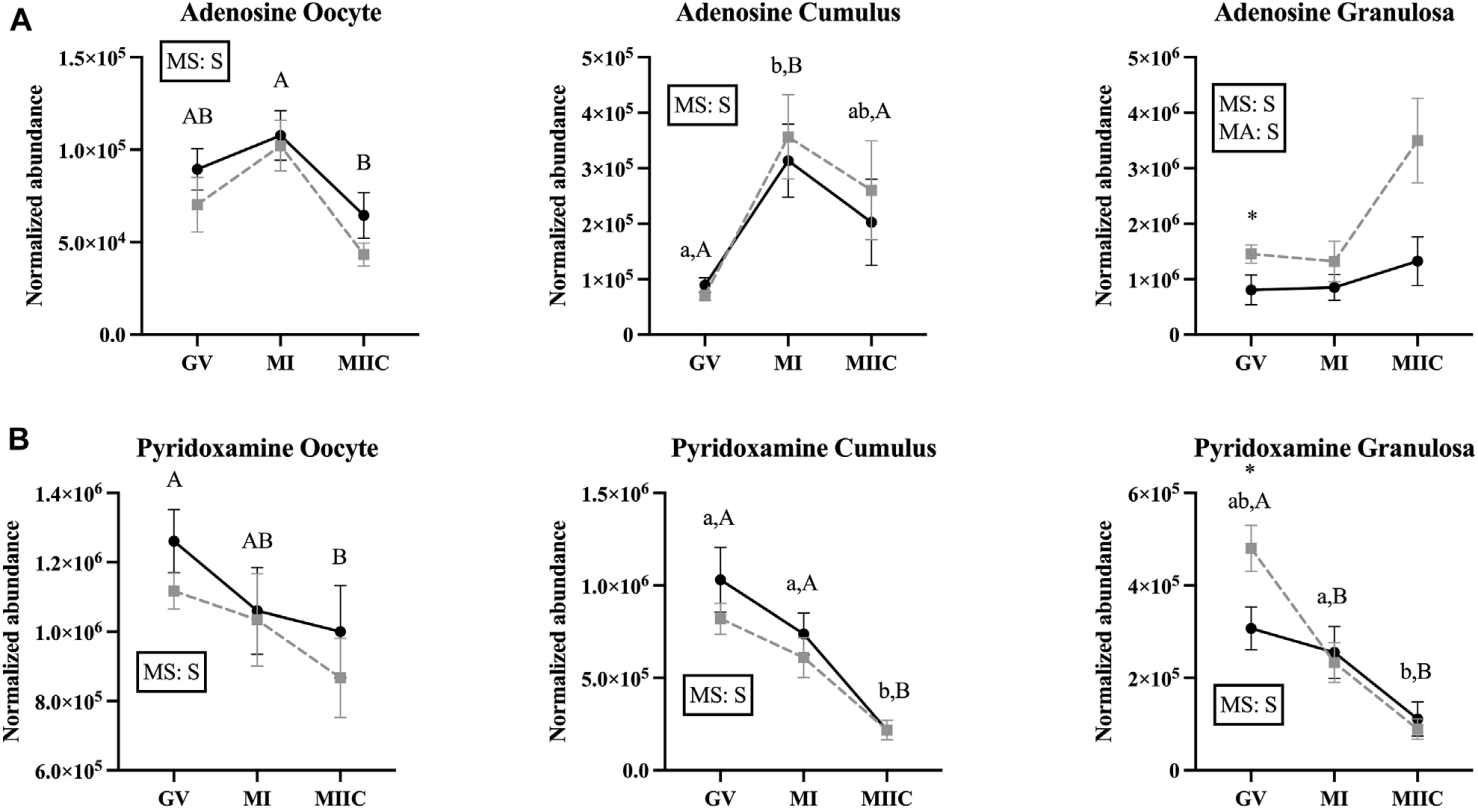
Normalized abundance of selected miscellaneous (uncategorized) compounds in oocytes (first column; Young, n = 8 and Old, n = 9), cumulus cells (second column; Young, n = 8 and Old, n = 6), and granulosa cells (third column; Young, n = 5 and Old, n = 9) collected from young mares (solid lines) and old mares (dotted lines) at 0 h (GV, germinal vesicle stage) or 24 h (MI, metaphase I stage) after maturation induction, with additional oocytes collected at 24 h and cultured *in vitro* for 18 h (MIIC, metaphase II stage after culture). Results are presented as mean ± SEM for: **(A)** adenosine, **(B)** pyridoxamine. Superscripts indicate differences (*p* < 0.05) among stages (GC, MI and MIIC) for Young (a,b,c) or Old (A,B,C), and between age groups at a specific stage (*). Significant (S, *p* < 0.05) main effects of stage (MS) or mare age (MA) are depicted in each graph. Interactions of stage-by-age were not significant (*p* > 0.1).

Main effect of stage differed (*p* < 0.05) in abundance of miscellaneous metabolites in oocytes (n = 8), cumulus cells (n = 23), and granulosa cells (n = 18); however, not all metabolites differed in all cell types (Supplementary Tables S1–S3). Adenosine differed by stage for all cell types and by a main effect of age (*p* = 0.03) in granulosa cells (Figure 8A). In oocytes, adenosine abundance was less in MIIC than MI in Old (*p* < 0.001, Figure 8A). However, in cumulus cells, adenosine abundance increased from GV to MI for Young and Old and then declined significantly to MIIC in Old (*p* < 0.001, Figure 8A). In cumulus and granulosa cells, but not oocytes, 2-hydroxy-pyridine differed by stage. In cumulus cells for Young and Old and in granulosa cells for Old, 2-hydroxy-pyridine was greater in MI than MIIC (Figure 8B, Supplementary Tables S2, S3). Pyridoxamine differed by stage for all cell types with lower abundance in MIIC than GV in oocytes from Old (*p* < 0.001, Figure 8B). Pyridoxamine was less in cumulus cells at MIIC when compared to GV and MI (*p* < 0.001, Figure 8B). In granulosa cells, pyridoxamine was greater in GV when compared to MI and MIIC in Old and in GV than MIIC when compared to MI in Young (*p* < 0.001, Figure 8B). Granulosa cells from Old had greater abundance of pyridoxamine than Young at GV (*p* < 0.05, Figure 8B).

## Discussion

Clinical assisted reproductive programs for equids, humans, and other species have been developed to obtain offspring from subfertile females or animals of high genetic value. The success of producing offspring using clinical assisted reproductive procedures is dependent on the viability and developmental potential of the oocyte, which can be affected by maternal factors, such as aging (Brinsko et al., 1995; Heffner, 2004; Altermatt et al., 2009). In addition, assisted reproductive procedures, e.g., standard *in vitro* fertilization or intracytoplasmic sperm injection (ICSI), require maturing or holding oocytes *in vitro*. Although *in vitro* systems have been constructed to maintain oocyte viability, they do not provide an equal environment to natural conditions. In most cases, this is to the detriment of gametes; however, the potential also exists to improve oocyte developmental potential with removal from a suboptimal maternal environment. The extent that limited intervals *in vitro* can affect the oocyte and associated follicular cells is not well documented. The present study was conducted to evaluate changes in the metabolite profile in oocytes, as well as cumulus and granulosa cells, as both are important to the follicular microenvironment which directly relates to oocyte quality (Gao et al., 2022).

The large oocyte consumes more energy than most somatic cells (Warzych and Lipinska, 2020) to perform essential functions, such as resumption of meiosis and cytoplasmic maturation (Xie et al., 2016). Carbohydrates can provide energy support within the ovarian follicle. Prior to LH initiating the ovulation cascade and resumption of meiosis, the oocyte is connected to the surrounding cumulus cells through transzonal projections, with gap junctions allowing for bi-directional communication (Albertini et al., 2001). One important carbohydrate for the ovarian follicle and oocyte is glucose, with differential use during maturation. Mature bovine COCs consume more glucose than immature COCs (Sutton et al., 2003), and an ovulatory stimulus transiently increases intrafollicular glucose in macaques (Brogan et al., 2011). Glucose concentrations can affect the oocyte’s meiotic and cytoplasmic maturation and cumulus cell function; glucose concentration that are too high or low can result in precocious resumption of nuclear maturation [reviewed in (Sutton-McDowall et al. (2010)]. Oocytes have a low capacity for glucose uptake and a low glycolytic rate; therefore, oocytes rely on cumulus cells to metabolize glucose and transport pyruvate or lactate through gap junctions (Sutton-McDowall et al., 2010; Herrick, 2019). However, within the oocyte, glycolysis likely has a role in maintenance of the redox potential; in addition, glucose in the oocyte can enter the pentose phosphate pathway, which can provide ribose sugars for nucleic acid and purine synthesis (Krisher, 2019). In general, we observed a significantly greater abundance of glucose in cumulus and granulosa cells at MIIC. This observation is somewhat consistent with *in vitro* matured porcine oocytes in which glucose increased over time during maturation at 0, 22 and 48 h of culture (Gao et al., 2023). The media used in the present study contains 5.6 mM glucose which is similar to the physiological concentrations of glucose reported in equine follicular fluid, ranging from 4.7 mM in small, immature follicles and 3.3 mM in follicles induced to mature using a crude equine pituitary gonadotropin (Collins et al., 1997). A concentration of 4.3 mM glucose was reported in follicular fluid collected from equine follicles >35 mm in diameter and the presence of uterine edema characteristic of estrus (Fernández-Hernández et al., 2020); however, these samples were collected *postmortem* and thus precise estrous cycle or follicle maturation stages are unknown. However, this glucose measurement more closely corresponds to previous samples in our laboratory of follicular fluid collected from live mares at 24 h after maturation induction; glucose concentrations were 4.5–4.8 mM (Carnevale, unpublished data). In the present study, oocytes and cumulus cells were cultured as COC from MI to MIIC. During *in vitro* maturation, equine COC take up more glucose than cumulus cells alone, indicating some glucose may be transferred directly to the oocyte; however, most glucose taken up by the COC is used in anaerobic glycolysis, resulting in the production of lactate (Lewis et al., 2020). Our findings are consistent with the previous study, as cumulus cells from young mares and granulosa cells from old mares had greater lactic acid abundance at MIIC, when glucose abundance was also greatest.

Although not a carbohydrate, pyruvic acid is derived mainly from glycolysis and is a major metabolic substrate for mitochondrial energy production in oocytes (Bradley and Swann, 2019). Oocyte growth and resumption of meiosis requires pyruvate as an energy source (Eppig, 1976). Additionally, pyruvate is important for oocyte metabolism (Gardner and Leese, 1990) and scavenging of reactive oxygen species (ROS) (O’Donnell-Tormey et al., 1987). The oocyte’s ability to metabolize glucose to produce pyruvate via glycolysis is limited by low activity of phosphofructokinase, the rate-limiting glycolysis enzyme, making the oocyte dependent on the transport of pyruvate from cumulus cells (Richani et al., 2021). Oocytes are proposed to secrete paracrine factors that promote the production of pyruvate in cumulus and granulosa cells to maintain adequate concentrations within the oocyte (Sugiura et al., 2005). In the present study, pyruvic acid was not affected by stage for granulosa cells, although differences were noted for oocytes and cumulus cells, suggesting a synchrony in the COC. The base medium used, TCM199, provides no pyruvate; however, the medium was supplemented with 0.2 mM pyruvate which is more than the 0.03–0.13 mM observed in equine follicular fluid (Gerard et al., 2000). The most notable changes with stage were observed for pyruvate abundance in cumulus cells, with a significant decline in MIIC than previous stages, suggesting an increase in metabolic needs.

Myo-inositol can be produced either as a derivative of glucose or through inositol phosphates (Chatree et al., 2020). In women with polycystic ovary syndrome, myo-inositol has been documented to improve systemic metabolic parameters such as lowering plasma triglyceride and low-density lipoprotein concentrations, while increasing high-density lipoprotein, improving insulin sensitivity (Minozzi et al., 2013), and decreasing hemoglobin A1C (Pintaudi et al., 2016). In addition, myo-inositol supplementation has been used to improve oocyte quality in women with polycystic ovarian syndrome (Merviel et al., 2021). In humans, follicular fluid with a higher myo-inositol concentration is associated with better oocyte quality (Chiu et al., 2002; Gupta et al., 2020). Metabolic profiling of bovine COCs in conditioned medium demonstrated that myo-inositol consumption increased in the last 15 h of a 23-h maturation interval; however, the cause or effect was not evaluated (Uhde et al., 2018). In the present study, myo-inositol was affected by stage in all cell types, with a general increase noted at MIIC. In young mares, myo-inositol increased in cumulus cells from GV to MIIC; in granulosa cells, myo-inositol was greater at the MIIC than MI for young and old mares. Therefore, the distinction between maturation or culture effects cannot be differentiated. While equine follicular fluid has not been evaluated for myo-inositol concentrations, human follicular fluid containing high quality oocytes has approximately 3.56 × 10^−2^ mM and is highly correlated to follicular fluid estradiol concentrations (Chiu et al., 2002). Potentially, the increase in myo-inositol was related to greater glucose uptake or synthesis from i-inositol (2.78 × 10^−4^ mM) in the culture medium (TCM199). The function of myo-inositol in cumulus and granulosa cells requires further study.

Sorbose is a monosaccharide ketose sugar (Wen et al., 2015), that limits glycolysis in some cell types, such as canine erythrocyte and murine cancer cells (Kistler and Keller, 1978; Xu et al., 2023). Additionally, in murine neoplastic cells, sorbose stimulates ROS production and contributes to apoptosis (Xu et al., 2023). In the present study, sorbose generally was limited at GV and MI, but was significantly greater in abundance at MIIC in all cell types, suggesting a possible association with culture. Sorbose has not been evaluated in mammalian follicle or follicular cells; however, sorbitol, which includes sorbose with two additional hydrogen atoms, has been studied in porcine and murine follicular cells. Supplementation of porcine COC with culture media containing 50 nM or more of sorbitol was associated with decreased nuclear maturation and cumulus expansion and with increased ROS production (Lin et al., 2015). Oocytes from aged mice have increased intracellular sorbitol; treating the oocytes with sorbinil to inhibit sorbitol accumulation resulted in reduced ROS and more oocytes extruding the first polar body (Zhang et al., 2021). Because sorbose can limit glycolysis, it would be worth investigating if increased sorbose could lead to the greater abundance of glucose and lower pyruvic acid as observed in the present study. In addition, evaluation into possible associations with sorbose and increased ROS production related to maturation to MII or short-term cell culture should be studied.

Lipids have diverse and essential biological roles, including energy storage, cell signaling, and formation of cellular membranes (Dunning et al., 2014). Lipid composition of oocytes varies greatly among species (Sturmey et al., 2009). Lipid composition of oocytes is important to developmental potential; for example, in canids, scarcity of lipids is evidence of degenerate oocytes (Durrant et al., 1998). In the peri-ovulatory phase of livestock reproduction, the role and content of lipids can be modulated *in vivo* by endocrine and nutritional factors (Sturmey et al., 2009). *In vitro*, changes to the composition of maturation culture media can alter the amount of lipids in oocytes and embryos (Abe et al., 1999; Rizos et al., 2002). In equids, a species with high lipid content, oocytes from obese mares have altered lipid profiles when compared to mares with a leaner body composition (Sessions-Bresnahan et al., 2016). Additionally, when equine COCs are matured *in vitro*, analysis of the “cumulome” indicates that fatty acid synthesis is upregulated (Walter et al., 2019). Therefore, various factors can affect the diverse population of lipids within the ovarian follicle.

In the present study, many lipids and fatty acids differed with stage, suggesting effects of maturation state or culture medium. Cholesterol is an essential component of animal cells; too much or too little cholesterol is potentially detrimental to cellular function (Arias et al., 2022). Cholesterol is a precursor of steroid hormones, bile acids, and vitamins. Cholesterol has two forms. The first form is free or unesterified cholesterol, which is an active form found in cell membranes (Luo et al., 2020). The second form is cholesteryl ester (CE) or esterified cholesterol, an inactive form resulting from esterification of unesterified cholesterol with fatty acids for storage in lipid droplets or as lipoproteins (Arias et al., 2022). While high-density lipoproteins (HDL) are the most abundant form of cholesterol in follicular fluid of mares (Le Goff, 1994), they are a poor substrate for steroidogenesis (Parinaud et al., 1987). However, certain molecular components from follicular fluid HDL relate to preimplantation embryo quality (Browne et al., 2009). Cholesterol depletion from mouse oocytes reduces oocyte activation and *in vitro* fertilization rates, likely due to changes in plasma membrane microdomains (Buschiazzo et al., 2008); but cholesterol repletion restores fertility (Buschiazzo et al., 2013). Oocytes cannot synthesize or take up cholesterol, so they rely on its transport through gap junctions with cumulus cells (Anderson and Albertini, 1976). However, oocytes can signal to cumulus cells to produce cholesterol through paracrine growth factors (Su et al., 2008). In the present study, cholesterol did not differ by stage for cumulus cells. However, cholesterol abundance was less in MIIC than MI in oocytes from old mares; while in granulosa cells, cholesterol declined from GV to MIIC in both age groups. Two cholesteryl esters, CE(18:2) and CE(22:5), were also affected by developmental stage. The first cholesteryl linoleate, CE(18:2), increased with maturation to MI in the somatic cells; however, continued maturation and/or cell culture led to a decline in cumulus cells. The second cholesteryl ester, CE(22:5) increased with maturation from GV to MIIC in cumulus and granulosa cells, regardless of mare age. The regulation of cholesterol homeostasis by esterification to CE and storage in lipid droplets is undetermined. However, hydrolysis of CE and esterification of free cholesterol may be used by bovine oocytes to regulate membrane cholesterol content (Buschiazzo et al., 2017). In the present study, free cholesterol was potentially esterified into CE(22:5) as maturation progressed or the cholesterol content supplied in TCM199 (5.17 × 10^−4^ mM) is deficient when compared to equine follicular fluid concentrations of 5.33 mM cholesterol (Collins et al., 1997). Alternatively, the cholesterol supplied in the TCM199 is not the HDL form predominant to equine follicular fluid (Sessions-Bresnahan et al., 2016). The causes and implications of the alteration in cholesterol in oocytes and granulosa in this study is an avenue for further investigation.

Octadecanoic acid, also known as stearic acid, was reduced in oocytes and granulosa cells at the MIIC stage and in cumulus cells from young mares. Stearic acid was also more abundant in oocytes from young mares at MIIC and cumulus cells at GV. Stearic acid is a predominant saturated free fatty acid in both human and bovine follicular fluid and has been associated with poor oocyte quality and decreased fertility in both species (Leroy et al., 2005; Jungheim et al., 2011; Van Hoeck et al., 2011). The decrease in stearic acid at the MIIC stage could have been an effect of maturation or the *in vitro* environment; any detrimental or beneficial effects on follicular cells is unknown. Metabolic profiling of *in vitro* matured porcine oocytes, another species with high oocyte lipid abundance, demonstrated that fatty acids declined with resumption of meiosis (Gao et al., 2023). In mice increased fatty acid oxidation is required for hormone induced meiotic maturation which could explain reduced stearic acid with maturation in this study.

In the present study, other lipid compounds that significantly differed with stage included diacylglycerides (DGs) and triacylglycerides (TGs). Diacylglycerols are made up of two fatty acids bound to a glycerol backbone with three possible locations on the backbone for fatty acids to bind. They function as components of cellular membranes, building blocks for glycero (phospho)lipids, and lipid second messengers (Eichmann and Lass, 2015). Because DGs are important for many different metabolic processes, they are tightly regulated; their function is highly related to which fatty acids are bound and where the fatty acids are located on the glycerol backbone (Eichmann and Lass, 2015). Intracellularly, DGs are located within different subcellular compartments, including the endoplasmic reticulum, Golgi apparatus, lipid droplets, and the plasma membrane (Eichmann and Lass, 2015). Diacylglycerides can be generated by the removal of a fatty acid from TGs; alternatively, DGs can be an intermediate during *de novo* synthesis of TGs (Eichmann and Lass, 2015). In this study, two DG isoforms were increased in oocytes at the MIIC stage in both age groups while an additional three were increased in oocytes from young mares. This is an intriguing finding based on the diversity of functions of DGs. One well-documented function that would occur in the MIIC oocyte is calcium oscillations, which are required at meiosis II after fertilization for activation of the oocyte (Swann and Yu, 2008; Gonzalez-Garcia et al., 2013). In mice, DGs were increased when exposed to peroxide-induced oxidative damage, but they were not increased in oocytes of aged animals (Mok et al., 2016). Further follow up on age differences in DGs in MIIC oocytes might provide some insight into age-related declines in oocyte quality in mares (Catandi et al., 2021), especially as associated with oocyte organelle function. Another intriguing study would be if a diet high in antioxidants, which was shown to improve oocyte development potential (Catandi et al., 2022), or culture medium supplementation with antioxidants could ameliorate differences associated with maternal aging.

Several TGs were different in cumulus and granulosa cells, but only 1 TG differed with stage in oocytes. The data provided no clear trend for increasing or decreasing TGs with maturation stage. The equine COC produces about 87% of its energy through oxidative phosphorylation; because equine oocytes appear to have abundant lipid vesicles, fatty acid oxidation is likely a major producer of ATP for equine oocytes (Grøndahl et al., 1995; Lewis et al., 2020). Triaclyglycerols are a major source of energy reserves and are present in the form of lipid droplets (Liu et al., 2022). While lipid droplet content varies amongst different species and developmental stages, generally oocytes are lipid rich to supply energy for meiosis (Genicot et al., 2005). Maternal factors, such as obesity, can alter the lipid fingerprint of oocytes, including the abundance of TGs within the equine oocyte (Sessions-Bresnahan et al., 2016). Further research into the modification of TGs during oocyte maturation would be worth pursuing, especially as associated with species differences in oocyte dependance on fatty acid oxidation.

Many additional lipids, which were primarily phospholipids such as phosphatidylcholines (PC), lysophosphatidylcholines (lysoPC), phosphatidylethanolamine (PE), lysophosphatidylethanolamine (lysoPE), phosphatidylserines (PS), and sphingomyelin, differed with stage and/or culture. It is beyond the scope of this paper to discuss all of them. However, it is worth noting their functional significance in cells. Phospholipids, including PC, PE, and PS are an integral part of the lipid bilayer between cells and their environments and between different subcellular compartments (Dai et al., 2021). In addition to forming barriers, the lipid bilayer may have crucial functions in the regulation of multiple cellular processes associated with the immune system and inflammation (Shimizu, 2009; Wu et al., 2016). Lysophosphatidylcholine have a host of functions associated with diseases (Law et al., 2019). In mouse granulosa cells lysoPC has been documented to increase apoptosis and inhibit cell viability (Yang et al., 2022). Physiological functions of lysoPE are not well defined, but evidence suggests that it has a role in modifying lipid metabolism and is a molecule of interest for research into non-alcoholic fatty liver disease (Yamamoto et al., 2022). Sphingomyelins (SM) are a dominant sphingolipid in mammalian cell membranes, particularly located in the plasma membrane, endocytic recycling compartment, and the trans Golgi network (Slotte, 2013); they are molecules of interest in the study of ovarian cancer (Jing et al., 2021). Targeted analysis of these molecules and how they may be involved in oocyte and follicle maturation is worthwhile, including the potential effects of cell culture on phospholipid composition.

The last lipid class to have significant effect on maturation or culture were ceramides and ceramide-related compounds. Ceramides are important for cellular function, having roles in proliferation, apoptosis, and signaling; ceramides are also components of cellular and mitochondrial membranes (Gomez-Larrauri et al., 2020). Like many other compounds, ceramides are transported to oocytes through gap junctions (Perez et al., 2005); however, their specific roles have not yet been elucidated. In our study, ceramides were not different in oocytes based on mare age or stage of maturation. This contrasts with previous work in mice that found ceramides were more abundant in oocytes from younger animals (Perez et al., 2005). However, while oocytes did not demonstrate differences, cumulus and granulosa cells did have changes in ceramide abundance, but there was variation in affected stage and age groups. Ceramide has pro-apoptotic activities in COCs and is an initiator of ROS generation during lipotoxic responses (Lolicato et al., 2015) and heat stress (Lee et al., 2021). The effect of ceramide changes in somatic cells on follicular function and oocyte maturation requires additional studies.

Amino acids and their derivatives were another class of metabolites that significantly differed with maturation stage and mare age. Amino acids are utilized in many different metabolic processes, including as substrates for the synthesis of proteins, nucleotides, glutathione, hyaluronic acid, signaling molecules, and energy; amino acids are essential for proper oocyte development and maturation (Collado-Fernandez et al., 2012). During follicular development in mice, fully grown oocytes can regulate amino acid uptake by cumulus and granulosa cells via gap junctions and paracrine signaling (Eppig et al., 2005). Also in mice, three amino acid transport systems are active during oocyte growth or maturation, while an additional three transport systems are only active during meiotic maturation, with each system transporting groups of related amino acids (Pelland et al., 2009). Similar research has not been conducted for the mare.

In the present study, glutamic acid and pyroglutamic acid were impacted by stage and mare age. In oocytes, normalized abundance of glutamic acid was elevated in old mares at all three maturation stages, additionally, pyroglutamic acid was elevated at the MIIC. Glutamic acid was elevated in the cumulus and granulosa of old mares at GV and MI and increased in abundance at the MIIC stage regardless of mare age. Pyroglutamic acid demonstrated similar abundance to glutamic acid, in that it was elevated at MIIC when compared to GV and MI. Glutamine and glutamate are derivatives of glutamic acid and can be used as substrates for synthesis of protein, energy, and glutathione, and can interact with cellular receptors as signaling molecules (Yoo et al., 2020; Špirková et al., 2022). Analysis of glutamine and glutamic acid by LC-MS have indicated that glutamine is crystalized into pyroglutamic acid at rates of 33% to almost 100% as an artifact of the analysis (Purwaha et al., 2014). In our analysis, pyroglutamic acid was detected via GC-MS; however, no studies could be found to determine if a similar phenomenon is occurring in GC-MS analysis. If this is possibly the case, the higher normalized abundance of pyroglutamic acid could be an indicator of glutamine abundance as well as or instead of endogenous pyroglutamic acid. Amino acid analysis of spent culture media from bovine *in vitro* maturation to MII reveals that glutamine is depleted from the media at high rates (Hemmings et al., 2012), which could explain the increased abundance of glutamic and pyroglutamic acid in the present study. Bovine LH increases glutamine oxidative metabolism by oocytes and COCs (Zuelke and Brackett, 1993), likely increasing uptake from media. Glutamine can also be metabolized into α-ketobutyrate (Cooper and Kuhara, 2014); therefore, this could account for the increased abundance of α-ketobutyrate observed in the present study after stimulation *in vivo*. In porcine oocytes, glutamic acid increases at 22 h after onset of maturation but decreases at 48 h (Gao et al., 2022). However, the porcine COCs were matured completely *in vitro*, as opposed to the oocytes and follicular cells of the present study which were stimulated *in vivo* and, therefore, exposed to follicular fluid for a longer time interval. Follicular fluid concentrations of glutamine have not been determined in mare follicular fluid; however, donkey glutamine follicular fluid concentrations range from 0.25 to 0.74 mM, with a mean concentration of 0.47 mM (Catalán et al., 2022). The concentration is similar to the 0.68 mM L-glutamine and 0.51 mM L-glutamic acid in the culture medium used in the present study. Like porcine oocytes, *in vivo* matured mouse oocytes’ glutamic acid and pyroglutamic acid increase at MI but decrease at MII (Li et al., 2020); however, neither study evaluated cumulus or granulosa cells, so no comparison can be made between studies for follicular cells. However, in human granulosa cells, exposure to follicular fluid with greater glutamine concentrations had greater expression of genes associated with antioxidant capacity, cellular proliferation, and steroidogenesis, as well as elevated concentrations of estradiol and estriol in follicular fluid (Wang et al., 2022).

Additional amino acids that were increased by maturation and/or cell culture in oocytes in the present study were alanine and threonine, while cysteine and glycyl-tyrosine were decreased. Glycine was detected at two separate molecular peaks with one derivative increasing while the other decreased at the MIIC stage. However, these were only significant in old mares and generally only impacted the MIIC stage. This suggests that further investigation into modifications of amino acid concentrations in culture media for oocytes from individuals of advance maternal age could be warranted. Our findings were somewhat in contrast to work in bovine *in vitro* maturation that found alanine and glycine were released into the media by cells (Hemmings et al., 2012). During *in vitro* maturation of bovine oocytes, transport of glycine, cystine and taurine peaks at MI and MII oocytes (Pelland et al., 2009). This increased transport may be reflective of the demand for glycine and cystine for glutathione synthesis occurring during oocyte maturation (Luberda, 2005; Pelland et al., 2009). Amino acid profiles in bovine maturation iv vitro indicate oocyte developmental potential, as oocytes with higher potential *versus* poorer quality present different amino acid profiles (Hemmings et al., 2012). Bovine oocytes that fail to cleave after *in vitro* fertilization deplete more glutamine from media and release more alanine into media, than oocytes that cleave and begin embryo development (Hemmings et al., 2012). Currently, similar studies have not been conducted in horses and other species.

In the present study, cumulus and granulosa cells had more alterations in amino acid profiles than oocytes. As in oocytes, two derivatives of glycine were detected with one greater and the other less in abundance at MIIC as compared to either GV or MI in both cell types. In cumulus cells, serine and cysteine were increased at MIIC for young mares; whereas, alanine and threonine were increased at MIIC for both age groups. Another derivative of amino acids, α-ketobutyrate was increased from GV to MI and remained elevated in MIIC, indicating a potential effect of follicle maturation and/or culture. Glycyl-tyrosine decreased at MIIC. Excess amino acid in cell culture media did not seem to be the cause of their increased abundance, as concentrations of amino acids in TCM199 were often lower than documented in follicular fluid. When comparing TCM199 medium to equine preovulatory follicular fluid, respectively, glycine concentrations were 0.67 mM *versus* 3.2 mM, alanine concentrations were 0.28 mM *versus* 1.1 mM, and threonine concentrations were 0.25 mM *versus* 0.35 mM (Fernández-Hernández et al., 2020). In granulosa cells, differences in amino acids among stages were more consistent between age groups when compared to cumulus cells; but alterations still occurred at MIIC. Alanine, α-ketobutyrate, serine, and threonine increased at MIIC in both age groups while cysteine and cysteinyl-proline were only different in cells from old mares. Glycyl-tyrosine and ɛ-polylysine were lower at MIIC. What, if any, impact this has on cumulus and granulosa cell function remains to be elucidated. It is important to consider that cumulus and granulosa cells at MIIC have different fates, cumulus cells aid in oocyte pick up by the oviduct and fertilization (Jones and Lopez, 2014), while granulosa will remain within the ovary to become large luteal cells, primarily responsible for hormone synthesis (Channing, 1966). Therefore, amino acid requirements for cumulus and granulosa cells could be very different.

In the present study, adenosine and pyridoxamine were also affected by stage or culture. Adenosine is a purine nucleoside that is important for maintaining oocyte meiotic arrest (Miller and Behrman, 1986; Salustri et al., 1988). Oocytes use adenosine to produce the ATP necessary for several cellular functions–including intracellular signaling, DNA and RNA synthesis, and purinergic signaling (Dunn and Grider, 2023). Additionally, adenosine is used for accumulation of cAMP in COCs, and inhibits oocyte maturation in a dose-dependent manner (Miller and Behrman, 1986). However, after ovulation, oocytes can no longer take up adenosine from the follicular fluid; therefore, they rely on adenosine acquired prior to ovulation (Wen et al., 2010). Adenosine decreases in follicular fluid with increasing follicular size in human and mouse follicles (Eppig et al., 1985; Wen et al., 2010). After induction of follicular maturation in mice, ATP consumption increases significantly in oocytes during the MI and MII stages, when compared to those arrested at the GV stage (Dalton et al., 2014). Consumption of ATP is enhanced when gap junctions between oocytes and cumulus cells are maintained (Dalton et al., 2014), possibly accounting for some of the loss of adenosine from follicular fluid with increasing follicle size (Eppig et al., 1985; Wen et al., 2010). However, cAMP is reduced following the LH surge, leading to activation of maturation promoting factors and allowing meiosis I to proceed (Strączyńska et al., 2022). In the present study, adenosine declined from MI to MIIC in oocytes from old mares. In cumulus adenosine increased from GV to MI; however, in old mares, adenosine abundance declined at MIIC. The TCM199 was supplemented with adenosine 5′-phosphate (5.67 × 10^−4^ mM) and adenosine 5′-triphosphate (1.65 × 10^−3^ mM). Equine follicular fluid concentrations of adenosine have not been determined; however, follicular fluid from mice prior to germinal vesicle breakdown contain 0.35–0.68 mM and after germinal vesicle breakdown contain 0.26–0.53 mM of adenosine (Eppig et al., 1985); these concentrations are higher than in the cell culture media. Potentially, the decrease in adenosine could be due to a decline in cAMP levels after ovulation initiation. Because of the age-associated differences, additional adenosine in culture medium from older females could be of interest.

Pyridoxamine is one of a few natural forms of vitamin B_6_ that is a critical intermediate in the transfer of amino groups by vitamin B_6_-dependent enzymes (Voziyan and Hudson, 2005). Pyridoxamine is also a metabolite of interest in preventing the formation of advanced glycation end products (AGEs) (Booth et al., 1996). Advanced glycation end products are modifications of proteins or lipids that contact aldose sugars and become nonenzymatically glycated, oxidized, and produce ROS leading to inflammation (Schmidt et al., 1994). The formation of AGEs is associated with complications of diabetes, atherosclerosis, neurodegenerative diseases, and aging (Thorpe and Baynes, 1996), and AGEs may contribute to age-associated declines in follicle and oocyte quality (Tatone and Amicarelli, 2013). However, the role of pyridoxine in the ovarian follicle is largely unknown. In this study, all 3 cell types exhibited decreasing abundance of pyridoxamine from the GV to MIIC in old mares. Young mares also demonstrated declining abundance in cumulus and granulosa cells. The decline began from GV to MI; however, it did not become significant until MIIC, making it hard to determine if this is maturation and/or culture related. The TCM199 medium was supplemented with pyridoxine hydrochloride, another form of vitamin B_6_, in a low concentration (1.12 × 10^–4^ mM, manufacturer’s specifications), and pyridoxine hydrochloride is not as efficient at preventing the formation of AGEs as pyridoxamine (Booth et al., 1996). The observation of less pyridoxamine with greater glucose at MIIC warrants further evaluation into any increase in AGEs associated with maternal aging or cell culture; supplementation of pyridoxamine in culture media could be warranted.

In the present study, a nontargeted metabolomics approach was used to investigate the metabolic profile of oocytes, cumulus cells, and granulosa cells during the GV, MI and MII stages using repeated samples obtained from the same mares and only using cells from dominant follicles that were considered destined for ovulation. Although the cellular metabolome provides information regarding a single moment in time, repeated sampling from the same mares helps us to further understand changes in the follicle and oocyte during maturation. The GV and MI oocytes were obtained *in vivo*, while additional MI oocytes were cultured for the completion of maturation to MII (MIIC), making it difficult to definitively determine if some alterations in metabolism were associated with culture or maturation. Overall, many of the significant differences in metabolites were observed for MIIC when compared to earlier stages of maturation. Differences were most obvious for energy and lipid metabolites and amino acids. The most notable differences were observed in cumulus cells. Within the ovarian follicle, the cumulus cells will uptake, convert and transport compounds to the oocyte, and our findings emphasize the importance of cumulus cells in modulating follicular components to support the oocyte.

For the completion of maturation (MIIC), we used aa limited culture interval an environment that has been proven to be successful at producing viable embryos and offspring; however, our findings demonstrate that limited exposure to *in vitro* conditions affect the cell metabolome. Results of the present study also support the use of metabolomics to assess media and further optimize culture methods, including for COCs from different maternal statuses. Additionally, our results suggest that evaluating cell culture additives to provide extra support for the COCs from older females could be beneficial and provides an opportunity to gather novel information and potential areas for future study.

## Data Availability

The original contributions presented in the study are deposited to the repository and are available here: https://doi.org/10.6084/m9.figshare.24052818.v2.
