# Supplementary material for: Maturation and culture affect the metabolomic profile of oocytes and follicular cells in young and old mares
Source: Front Cell Dev Biol. 2024 Jan 12;11:1280998. doi: 10.3389/fcell.2023.1280998 (PMC10811030; doi:10.3389/fcell.2023.1280998)
Supplement: Supplementary file 1 [file Table1.DOCX]

Supplementary Table 1: Relative abundance of annotated metabolites in oocytes. Oocytes were collected from young mares (Yg, n=8) and old mares (Old, n=9) at 0h (GV) or 24h (MI) after maturation induction, with some oocytes collected at 24h and cultured for an additional 18h (MIIC). Results are presented as mean ± SEM, correction factor of 10^x^ (CF), P-values for overall effects of maturation stage (MS) and the interaction (INT) of age with maturation stage are included in the table. Different superscripts within a row represent differences at ^a,b,c^P < 0.05 or ^d,e,f^P < 0.1. Superscripts within a column for the same metabolite represent differences between Yg and Old at ^*^P < 0.05 or ^+^P < 0.1. The main effect of age was significant (P<0.05) for compounds with Yg and Old highlighted in grey. Abbreviated compound names are in the table, numerical superscripts indicate full name is at the bottom of the table.

| **Class and Metabolites** | **Age** | **GV** | **MI** | **MIIC** | **CF** | **MS** | **INT** |
| --- | --- | --- | --- | --- | --- | --- | --- |
| **Carbohydrates and derivatives** | | | | | | | |
| Glucose (1MEOX) (5TMS) MP | Yg | 4.62 ± 1.11 | 6.59 ± 1.08 | 19.19 ± 6.68 | 10^5^ | <0.001 | 0.50 |
|  | Old | 5.42 ± 1.21^ab,d^ | 7.40 ± 1.57^a,de^ | 24.16 ± 6.40^b,e^ |  |  |  |
| Glucose (1MEOX) (5TMS) MP | Yg | 6.27 ± 1.24 | 9.65 ± 1.55 | 33.04 ± 13.20 | 10^6^ | <0.001 | 0.50 |
|  | Old | 7.72 ± 1.66^d^ | 11.11 ± 2.37^d^ | 41.47 ± 11.69^e^ |  |  |  |
| Glucose (1MEOX) (5TMS) BP | Yg | 1.65 ± 0.11 | 1.91 ± 0.14 | 4.09 ± 1.08 | 10^6^ | <0.001 | 0.40 |
|  | Old | 1.78 ± 0.21^a^ | 2.09 ± 0.20^a^ | 5.11 ± 1.08^b^ |  |  |  |
| Inositol, myo- (6TMS) | Yg | 11.27 ± 1.18 | 10.17 ± 0.34 | 14.63 ± 2.73 | 10^5^ | 0.03 | 0.99 |
|  | Old | 10.17 ± 0.30 | 9.22 ± 0.73 | 13.42 ± 1.66 |  |  |  |
| Inositol-2-phosphate, myo- (7TMS) | Yg | 2.87 ± 0.72^de^ | 4.18 ± 0.89^d^ | 1.55 ± 0.35^e^ | 10^5^ | <0.001 | 0.57 |
|  | Old | 3.02 ± 0.37^a,d^ | 4.69 ± 0.51^a,e^ | 1.30 ± 0.15^b,de^ |  |  |  |
| Pyruvic acid (1MEOX) (1TMS) | Yg | 5.43 ± 0.18^+^ | 5.09 ± 0.18 | 4.82 ± 0.32 | 10^5^ | 0.03 | 0.75 |
|  | Old | 4.81 ± 0.25^+^ | 4.75 ± 0.31 | 4.51 ± 0.38 |  |  |  |
| Sorbose (1MEOX) (5TMS) BP | Yg | 9.15 ± 0.72^*^ | 8.88 ± 0.44^*^ | 41.72 ± 14.85 | 10^5^ | <0.001 | 0.34 |
|  | Old | 7.37 ± 0.37^a,*^ | 7.53 ± 0.38^a,*^ | 55.05 ± 13.97^b^ |  |  |  |
| Xylose (1MEOX) (4TMS) MP | Yg | 9.08 ± 0.54^d,*^ | 7.86 ± 0.41^e^ | 8.00 ± 0.67^de^ | 10^5^ | 0.12 | 0.25 |
|  | Old | 7.50 ± 0.46^*^ | 7.33 ± 0.65 | 7.24 ± 0.69 |  |  |  |
| **Lipids and Fatty Acids** | |  |  |  |  |  |  |
| 3beta-Cholest-xxx-icosatetraenoate^1^ | Yg | 9.56 ± 1.83 | 6.85 ± 0.80 | 25.37 ± 9.23 | 10^2^ | 0.02 | 0.74 |
|  | Old | 7.29 ± 1.46 | 6.23 ± 0.72 | 29.35 ± 1.20 |  |  |  |
| 4-Hydroxy-3-xxx-pyran-2-one^2^ | Yg | 6.31 ± 3.90 | 50.19 ± 24.37 | 0.57 ± 0.14 | 10^3^ | 0.01 | 0.75 |
|  | Old | 26.15 ± 16.93 | 71.16 ± 25.37 | 4.35 ± 3.96 |  |  |  |
| Cholesterol (1TMS) | Yg | 12.2 ± 0.96^d^ | 11.31 ± 0.84^de^ | 9.54 ± 0.86^e^ | 10^5^ | <0.001 | 0.59 |
|  | Old | 12.45 ± 0.38^ab,d^ | 12.49 ± 0.57^a,de^ | 10.04 ± 0.88^b,e^ |  |  |  |
| Hexadecanoic acid (1TMS) | Yg | 1.76 ± 0.17^ab,d^ | 2.13 ± 0.20^a,de^ | 1.46 ± 0.14^b,e,*^ | 10^7^ | <0.001 | 0.31 |
|  | Old | 1.62 ± 0.10^a^ | 2.04 ± 0.15^b^ | 1.13 ± 0.07^c,*^ |  |  |  |
| Lipoyl-GMP | Yg | 2.26 ± 1.19 | 13.74 ± 6.77 | 0.43 ± 0.09 | 10^3^ | <0.001 | 0.84 |
|  | Old | 8.07 ± 3.85 | 15.90 ± 4.83 | 2.45 ± 1.92 |  |  |  |
|  |  |  |  |  |  |  |  |
|  |  |  |  |  |  |  |  |
| N-(xxx)stearamid^3^ | Yg | 11.82 ± 1.18 | 11.49 ± 1.05^*^ | 12.73 ± 0.61 | 10^4^ | 0.04 | 0.16 |
|  | Old | 11.20 ± 0.92^de^ | 8.45 ± 1.17^d,*^ | 11.97 ± 1.04^e^ |  |  |  |
| Octadecanoic acid (1TMS) | Yg | 12.06 ± 1.64^ab^ | 14.40 ± 2.14^a^ | 10.26 ± 1.53^b,*^ | 10^6^ | <0.001 | 0.23 |
|  | Old | 9.94 ± 1.01^a,d^ | 12.96 ± 1.35^a,e^ | 6.73 ± 0.84^b,de,*^ |  |  |  |
| Octadecenoic acid, 9-(E)- (1TMS) | Yg | 1.90 ± 0.18^ab,d^ | 2.22 ± 0.22^a,de^ | 1.33 ± 0.14^b,e,*^ | 10^6^ | <0.001 | 0.29 |
|  | Old | 1.71 ± 0.12^a^ | 2.21 ± 0.18^b^ | 0.96 ± 0.09^c,*^ |  |  |  |
| Octadecadienoic acid, 9,12-(Z,Z)- (1TMS) | Yg | 15.67 ± 2.12 | 19.20 ± 2.51 | 18.31 ± 5.08^+^ | 10^4^ | 0.05 | 0.03 |
|  | Old | 18.41 ± 1.59^a^ | 19.74 ± 2.79^a^ | 8.19 ± 1.17^b,+^ |  |  |  |
| CE(18:2) | Yg | 3.25 ± 0.50 | 16.40 ± 5.59 | 5.37 ± 1.09 | 10^3^ | 0.034 | 0.22 |
|  | Old | 4.08 ± 1.86 | 7.64 ± 2.66 | 4.85 ± 1.33 |  |  |  |
| DG(32:0) | Yg | 6.97 ± 1.01^de^ | 6.49 ± 0.76^d^ | 8.55 ± 0.38^e^ | 10^4^ | 0.09 | 0.78 |
|  | Old | 6.82 ± 0.65 | 5.46 ± 0.72 | 7.59 ± 0.73 |  |  |  |
| DG(34:1) | Yg | 2.28 ± 0.20^d^ | 1.93 ± 0.14^de^ | 2.97 ± 0.35^e^ | 10^4^ | <0.001 | 0.41 |
|  | Old | 2.77 ± 0.32 | 1.99 ± 0.25 | 2.90 ± 0.29 |  |  |  |
| DG(34:3) | Yg | 8.81 ± 1.07 | 8.06 ± 0.88 | 10.82 ± 1.10 | 10^3^ | 0.02 | 0.45 |
|  | Old | 8.87 ± 0.67 | 6.56 ± 0.73 | 12.16 ± 2.32 |  |  |  |
| DG(36:2) | Yg | 10.63 ± 1.03^ab^ | 8.35 ± 0.89^a^ | 11.89 ± 1.25^b^ | 10^3^ | 0.01 | 0.66 |
|  | Old | 10.23 ± 1.10 | 9.33 ± 1.34 | 13.74 ± 1.80 |  |  |  |
| DG(36:2) | Yg | 2.85 ± 0.27 | 2.54 ± 0.17 | 3.38 ± 0.35 | 10^4^ | 0.01 | 0.25 |
|  | Old | 3.30 ± 0.40 | 2.45 ± 0.39 | 4.68 ± 0.75 |  |  |  |
| DG(36:3) | Yg | 1.80 ± 0.24 | 1.45 ± 0.10^+^ | 2.07 ± 0.19 | 10^4^ | <0.001 | 0.80 |
|  | Old | 1.61 ± 0.09^a^ | 1.14 ± 0.13^ab,+^ | 2.05 ± 0.30^b^ |  |  |  |
| DG(36:4) | Yg | 7.15 ± 0.64^ab^ | 5.32 ± 0.24^a^ | 8.08 ± 0.41^b^ | 10^3^ | <0.001 | 0.64 |
|  | Old | 5.98 ± 0.22^ab^ | 4.77 ± 0.50^a^ | 7.95 ± 0.88^b^ |  |  |  |
| DG(40:8) | Yg | 4.08 ± 0.21 | 3.33 ± 0.34^*^ | 4.69 ± 0.43 | 10^3^ | 0.01 | 0.56 |
|  | Old | 3.38 ± 0.35^d^ | 2.44 ± 0.23^e,*^ | 4.68 ± 0.80^de^ |  |  |  |
| lysoPC(14:0) | Yg | 10.22 ± 1.57 | 5.90 ± 1.19 | 6.02 ± 1.66 | 10^2^ | 0.03 | 0.41 |
|  | Old | 15.95 ± 0.55 | 5.39 ± 0.13 | 5.64 ± 0.15 |  |  |  |
| lysoPC(O-16:0) | Yg | 1.69 ± 0.23 | 1.22 ± 0.18 | 1.88 ± 0.29 | 10^3^ | <0.001 | 0.05 |
|  | Old | 2.45 ± 0.29 | 1.09 ± 0.27 | 1.32 ± 0.30 |  |  |  |
| lysoPC(16:1) | Yg | 1.88 ± 0.33 | 1.01 ± 0.18 | 1.82 ± 0.24 | 10^3^ | 0.02 | 0.65 |
|  | Old | 1.66 ± 0.34 | 1.25 ± 0.31 | 2.01 ± 0.30 |  |  |  |
| lysoPC(20:4) | Yg | 1.73 ± 0.26 | 1.01 ± 0.24 | 1.46 ± 0.22 | 10^3^ | 0.02 | 0.37 |
|  | Old | 1.32 ± 0.18 | 0.85 ± 0.16 | 1.73 ± 0.34 |  |  |  |
| TG(49:1) | Yg | 1.79 ± 0.47^de^ | 2.57 ± 0.53^d^ | 0.96 ± 0.17^e^ | 10^4^ | 0.05 | 0.24 |
|  | Old | 3.53 ± 1.00^de^ | 2.46 ± 0.40^d^ | 1.49 ± 0.34^e^ |  |  |  |
|  | | |  |  |  |  |  |
|  | | |  |  |  |  |  |
| **Amino Acids and Derivatives** | | |  |  |  |  |  |
| α‐ketobutyrate | Yg | 2.14 ± 0.38^d^ | 3.52 ± 0.27^e,*^ | 3.87 ± 0.81^e^ | 10^3^ | 0.02 | 0.41 |
|  | Old | 2.00 ± 0.26 | 2.23 ± 0.27^*^ | 3.64 ± 0.73 |  |  |  |
| Alanine (2TMS) | Yg | 11.70 ± 0.79^+^ | 11.16 ± 0.69^*^ | 13.54 ± 1.16 | 10^6^ | <0.001 | 0.17 |
|  | Old | 9.81 ± 0.68^ab,+^ | 8.13 ± 0.53b^a*^ | 12.82 ± 0.98^b^ |  |  |  |
| Cysteine (3TMS) | Yg | 2.87 ± 0.35 | 3.02 ± 0.69 | 1.85 ± 0.21 | 10^5^ | <0.001 | 0.64 |
|  | Old | 2.18 ± 0.31^a^ | 2.98 ± 0.48^a^ | 1.44 ± 0.26^b^ |  |  |  |
| ɛ-polylysine | Yg | 0.66 ± 0.42 | 3.65 ± 2.21 | 0.08 ± 0.03 | 10^4^ | 0.03 | 0.64 |
|  | Old | 3.76 ± 2.36 | 5.08 ± 2.82 | 0.52 ± 0.44 |  |  |  |
| Glutamic acid (3TMS) | Yg | 6.84 ± 0.61^+^ | 7.66 ± 1.30^+^ | 9.96 ± 2.36^+^ | 10^5^ | 0.02 | 0.27 |
|  | Old | 14.01 ± 3.48^+^ | 13.25  ± 2.23^+^ | 20.37 ± 4.44^+^ |  |  |  |
| Glycine (3TMS) | Yg | 28.81 ± 0.54 | 27.09 ± 0.67^*^ | 30.16 ± 1.66 | 10^6^ | 0.01 | 0.12 |
|  | Old | 28.53 ± 0.76^d^ | 29.40 ± 0.57^de,*^ | 35.48 ± 2.49^e^ |  |  |  |
| Glycyl-Tyrosine | Yg | 31.62 ± 19.04 | 63.65 ± 25.08 | 1.42 ± 0.70 | 10^4^ | <0.001 | 0.88 |
|  | Old | 57.37 ± 20.46^ab^ | 92.38 ± 23.49^a^ | 14.25 ± 12.85^b^ |  |  |  |
| Pyroglutamic acid (2TMS) | Yg | 5.10 ± 0.58 | 5.95 ± 1.20 | 9.56 ± 3.09 | 10^6^ | <0.001 | 0.16 |
|  | Old | 6.06 ± 1.09^a^ | 7.42 ± 0.70^a^ | 15.80 ± 3.03^b^ |  |  |  |
| Threonine (3TMS) | Yg | 5.51 ± 0.47 | 6.51 ± 1.28 | 8.53 ± 2.28 | 10^5^ | <0.001 | 0.10 |
|  | Old | 4.66 ± 0.69^ab,d^ | 4.97 ± 0.51^a,de^ | 12.03 ± 2.17^b,e^ |  |  |  |
| **Miscellaneous** |  |  |  |  |  |  |  |
| Adenosine (3TMS) (Derivate not found) | Yg | 8.94 ± 1.12 | 10.77 ± 1.34 | 6.45 ± 1.23 | 10^4^ | <0.001 | 0.75 |
|  | Old | 7.03 ± 1.48^ab^ | 10.22 ± 1.37^a^ | 4.34 ± 0.62^b^ |  |  |  |
| Adenosine (3TMS) (Derivate not found) | Yg | 6.14 ± 0.50 | 5.65 ± 0.56 | 4.53 ± 0.46^*^ | 10^4^ | 0.001 | 0.07 |
|  | Old | 4.52 ± 0.75^ab^ | 6.17 ± 0.62^a^ | 3.00 ± 0.39^b,*^ |  |  |  |
| Adenosine | Yg | 1.32 ± 0.31 | 1.24 ± 0.25 | 3.31 ± 1.15 | 10^3^ | 0.03 | 0.83 |
|  | Old | 1.87 ± 0.38 | 1.73 ± 0.32 | 4.29 ± 1.63 |  |  |  |
| Phosphoric acid (3TMS) | Yg | 2.58 ± 0.37^a,+^ | 4.65 ± 0.64^b^ | 2.18 ± 0.30^a^ | 10^7^ | 0.01 | 0.41 |
|  | Old | 3.72 ± 0.41^a,de,+^ | 7.04 ± 1.93^ab,d^ | 2.66 ± 0.34^b,e^ |  |  |  |
| Phosphoric acid monomethyl ester^4^ | Yg | 12.95 ± 2.12^d,+^ | 23.77 ± 2.91^a,e^ | 8.79 ± 1.27^b,de^ | 10^5^ | <0.001 | 0.20 |
|  | Old | 19.10 ± 2.67^a,d,+^ | 33.80 ± 5.63^a,e^ | 9.60 ± 0.82^b,dc^ |  |  |  |
| Putrescine (3TMS) | Yg | 2.82 ± 0.10^d,+^ | 2.43 ± 0.17^e,+^ | 2.41 ± 0.21^de,+^ | 10^6^ | 0.78 | 0.50 |
|  | Old | 13.25 ± 5.09^+^ | 17.66 ± 8.12^+^ | 14.43 ± 6.11^+^ |  |  |  |
| Pyridoxamine (3TMS) | Yg | 12.61 ± 0.91 | 10.60 ± 1.25 | 10.00 ± 1.33 | 10^5^ | <0.001 | 0.62 |
|  | Old | 11.17 ± 0.52^a^ | 10.34 ± 1.33^ab^ | 8.67 ± 1.14^b^ |  |  |  |
| 2R-2(Palmitoyloxy)-xxx- heptadecanoate^5^ | Yg | 4.11 ± 0.60 | 3.18 ± 0.33 | 4.42 ± 0.34 | 10^3^ | 0.01 | 0.92 |
|  | Old | 3.83 ± 0.36^d^ | 2.70 ± 0.24^e^ | 3.80 ± 0.43^de^ |  |  |  |

^1^3beta-Cholest-5-en-3-yl (5Z,8Z,11Z,14Z)-5,8,11,14-icosatetraenoate; ^2^4-Hydroxy-3-[(1E)-5-oxo-1,7-diphenyl-1-hepten-3-yl]-6-[(E)-2-phenylvinyl]-2H-pyran-2-one; ^3^N-(2-stearamidoethyl)stearamide; ^4^Phosphoric acid monomethyl ester (2TMS); ^5^2R-2-(Palmitoyloxy)-3-(phosphonooxy)propyl heptadecanoate
