# Supplementary material for: Maturation and culture affect the metabolomic profile of oocytes and follicular cells in young and old mares
Source: Front Cell Dev Biol. 2024 Jan 12;11:1280998. doi: 10.3389/fcell.2023.1280998 (PMC10811030; doi:10.3389/fcell.2023.1280998)
Supplement: Supplementary file 2 [file Table2.DOCX]

Supplementary Table 2: Relative abundance of annotated metabolites in cumulus cells. Cumulus cells were collected from young mares (Yg, n=8) and old mares (Old, n=6) at 0h (GV) or 24h (MI) after maturation induction, with some cumulus cells collected at 24h and cultured for an additional 18h (MIIC). Results are presented as mean ± SEM, correction factor of 10^x^ (CF), P-values for overall effects of maturation stage (MS) and the interaction (INT) of age with maturation stage are included in the table. Different superscripts within a row represent differences at ^a,b,c^P < 0.05 or ^d,e,f^P < 0.1. Superscripts within a column for the same metabolite represent differences between Yg and Old at ^*^P < 0.05 or ^+^P < 0.1. The main effect of age was significant (P<0.05) for compounds with Yg and Old highlighted in grey. Abbreviated compound names are in the table, numerical superscripts indicate full name is at the bottom of the table.

| Class and Metabolites | Age | GV | MI | MIIC | CF | MS | INT |
| --- | --- | --- | --- | --- | --- | --- | --- |
| Carbohydrates and derivatives | | |  |  |  |  |  |
| Glucose (1MEOX) (5TMS) MP | Yg | 1.15 ± 0.33^a^ | 2.11 ± 0.66^a^ | 19.56 ± 3.48^b^ | 10^6^ | <0.001 | 0.77 |
|  | Old | 1.85 ± 1.04^a^ | 3.38 ± 1.43^a^ | 22.49 ± 5.26^b^ |  |  |  |
| Glucose (1MEOX) (5TMS) MP | Yg | 1.68 ± 0.45^a^ | 3.28 ± 0.92^a^ | 49.37 ± 5.12^b^ | 10^7^ | <0.001 | 0.30 |
|  | Old | 2.04 ± 1.01^a^ | 7.61 ± 3.90^a^ | 41.32 ± 10.12^b^ |  |  |  |
| Glucose (1MEOX) (5TMS) BP | Yg | 3.24 ± 0.84^a^ | 4.16 ± 1.01^a^ | 33.59 ± 4.37^b^ | 10^6^ | <0.001 | 0.78 |
|  | Old | 3.83 ± 1.66^a^ | 6.66 ± 2.47^a^ | 37.54 ± 7.87^b^ |  |  |  |
| Myo-inositol | Yg | 1.68 ± 0.36^a,de^ | 2.56 ± 0.46^ab,d*^ | 22.36 ± 6.42^b,e^ | 10^6^ | <0.001 | 0.49 |
|  | Old | 1.21 ± 0.45^d^ | 5.11 ± 1.07^e*^ | 17.94 ± 4.86^f^ |  |  |  |
| Inositol-2-phosphate, myo-^1^ | Yg | 1.68 ± 0.27^ab,d^ | 2.76 ± 0.36^a,e^ | 1.09 ± 0.16^b,de^ | 10^5^ | 0.03 | 0.62 |
|  | Old | 2.46 ± 1.01 | 2.55 ± 0.66 | 1.38 ± 0.35 |  |  |  |
| Lactic acid (2TMS) | Yg | 4.35 ± 0.88^ab^ | 2.19 ± 0.43^a^ | 3.78 ± 0.49^b^ | 10^8^ | 0.02 | 0.96 |
|  | Old | 5.16 ± 1.07 | 2.63 ± 0.62 | 4.20 ± 0.69 |  |  |  |
| Pyruvic acid (1MEOX) (1TMS) | Yg | 6.11 ± 0.57^a^ | 5.56 ± 0.23^a^ | 2.98 ± 0.29^b^ | 10^6^ | <0.001 | 0.57 |
|  | Old | 5.53 ± 1.03^a^ | 6.02 ± 0.75^ab^ | 3.34 ± 1.01^b^ |  |  |  |
| Sorbose (1MEOX) (5TMS) BP | Yg | 0.64 ± 0.06^a^ | 1.10 ± 0.20^a^ | 36.23 ± 7.49^b^ | 10^6^ | <0.001 | 0.67 |
|  | Old | 0.77 ± 0.06^a^ | 1.04 ± 0.09^a^ | 39.78 ± 4.36^b^ |  |  |  |
| Lipids and Fatty Acids | | | |  |  |  |  |
| 1-xxx-tridecyl palmitate^2^ | Yg | 2.02 ± 0.29^ab,*^ | 1.85 ± 0.19^a^ | 1.00 ± 0.15^b^ | 10^4^ | 0.02 | 0.11 |
|  | Old | 5.79 ± 1.80^*^ | 2.39 ± 0.40 | 1.81 ± 0.61 |  |  |  |
| (3beta)-Cholest-xxx-eicosatetraenoate^3^ | Yg | 3.91 ± 1.35^a^ | 18.85 ± 4.21^b^ | 374.48 ± 84.12^c^ | 10^2^ | <0.001 | 0.21 |
|  | Old | 6.05 ± 2.33^a^ | 12.66 ± 2.54^b^ | 230.03 ± 46.31^c^ |  |  |  |
| 3-(Palmitoyloxy)-xxx-octadecenoate^4^ | Yg | 5.50 ± 0.62 | 6.29 ± 0.58^*^ | 9.20 ± 1.28 | 10^4^ | 0.02 | 0.39 |
|  | Old | 5.38 ± 0.50 | 9.74 ± 1.63^*^ | 10.67 ± 2.12 |  |  |  |
| 4-Hydroxy-xxx-2H-pyran-2-one^5^ | Yg | 67.85 ± 29.42^ab^ | 116.87 ± 34.51^a^ | 0.63 ± 0.29^b^ | 10^3^ | <0.001 | 0.40 |
|  | Old | 22.74 ± 16.50^b^ | 116.54 ± 20.63^a^ | 25.92 ± 25.59^ab^ |  |  |  |
| Cholesterol (1TMS) | Yg | 1.13 ± 0.09^d^ | 3.21 ± 0.66^e^ | 1.76 ± 0.37^de^ | 10^6^ | 0.07 | 0.44 |
|  | Old | 2.04 ± 1.01 | 2.74 ± 0.41 | 2.49 ± 0.82 |  |  |  |
| Cholesterol | Yg | 7.45 ± 1.39^d^ | 14.22 ± 2.67^de^ | 16.81 ± 2.73^e^ | 10^3^ | <0.01 | 0.38 |
|  | Old | 8.21 ± 1.57 | 9.36 ± 1.88 | 16.53 ± 3.12 |  |  |  |
| 5a-Cholestanol | Yg | 18.54 ± 3.45^d^ | 8.33 ± 2.57^de^ | 5.97 ± 0.92^e^ | 10^3^ | 0.04 | 0.40 |
|  | Old | 31.88 ± 17.60 | 6.94 ± 1.60 | 5.17 ± 1.46 |  |  |  |
| ethyl 2-xxx-acetate^6^ | Yg | 5.48 ± 1.43^d^ | 1.22 ± 0.38^e^ | 5.18 ± 1.66^de^ | 10^4^ | 0.02 | 0.19 |
|  | Old | 7.31 ± 1.38^d^ | 2.08 ± 1.12^de^ | 1.96 ± 0.95^e^ |  |  |  |
| Hexadecanoic acid (1TMS) | Yg | 16.89 ± 1.67^a^ | 17.48 ± 2.29^a^ | 5.84 ± 0.70^b^ | 10^6^ | <0.001 | 0.30 |
|  | Old | 15.94 ± 2.71^a^ | 13.05 ± 1.99^ab^ | 6.18 ± 1.30^b^ |  |  |  |
| Linoleic acid | Yg | 14.59 ± 4.14^ab^ | 10.26 ± 2.57^a^ | 5.42 ± 1.22^b^ | 10^2^ | <0.001 | 0.81 |
|  | Old | 14.19 ± 1.08 | 6.58 ± 1.40 | 4.15 ± 2.37 |  |  |  |
| Lipoyl-GMP | Yg | 156.75 ± 63.13^ab^ | 130.54 ± 30.09^a^ | 5.18 ± 1.74^b^ | 10^2^ | 0.01 | 0.20 |
|  | Old | 69.40 ± 38.63^d^ | 179.10 ± 37.47^e^ | 31.32 ± 28.21^de^ |  |  |  |
| Octadecanoic acid (1TMS) | Yg | 12.78 ± 1.70^a,*^ | 13.55 ± 1.67^a^ | 3.15 ± 0.33^b^ | 10^6^ | <0.001 | 0.18 |
|  | Old | 8.31 ± 1.10^d,*^ | 10.29 ± 1.86^de^ | 3.37 ± 0.97^e^ |  |  |  |
| Octadecenoic acid, 9-(E)- (1TMS) | Yg | 13.67 ± 1.33^a^ | 17.70 ± 1.53^a^ | 5.35 ± 0.44^b^ | 10^5^ | <0.001 | 0.59 |
|  | Old | 12.75 ± 1.33^a^ | 16.19 ± 2.16^a^ | 6.67 ± 1.09^b^ |  |  |  |
| Octadecadienoic acid, 9,12-(Z,Z)^7^ | Yg | 20.77 ± 5.26^ab^ | 19.17 ± 1.77^a^ | 3.81 ± 0.46^b^ | 10^4^ | <0.001 | 0.55 |
|  | Old | 16.46 ± 5.06^a^ | 18.46 ± 1.31^a^ | 5.39 ± 0.98^b^ |  |  |  |
| CE(18:2) | Yg | 3.27 ± 0.35^a^ | 188.46 ± 31.29^b^ | 44.48 ± 8.55^c^ | 10^3^ | <0.001 | 0.19 |
|  | Old | 4.38 ± 1.13^a^ | 125.85 ± 21.89^b^ | 31.49 ± 5.33^c^ |  |  |  |
| CE(22:5) | Yg | 6.62 ± 1.17^a^ | 4.75 ± 1.42^a^ | 33.71 ± 5.50^b^ | 10^2^ | <0.001 | 0.78 |
|  | Old | 9.43 ± 2.49^ab,d^ | 3.63 ± 1.30^a,de^ | 35.11 ± 7.72^b,e^ |  |  |  |
| DG(20:7) | Yg | 8.63 ± 1.04^*^ | 7.01 ± 2.24 | 11.20 ± 1.34 | 10^2^ | <0.01 | 0.01 |
|  | Old | 21.48 ± 5.36^*^ | 5.19 ± 0.89 | 10.50 ± 1.90 |  |  |  |
| DG(32:0) | Yg | 6.21 ± 1.13^d^ | 2.50 ± 0.85^de^ | 2.48 ± 0.44^e^ | 10^4^ | <0.01 | 0.80 |
|  | Old | 4.74 ± 0.99 | 1.32 ± 0.28 | 1.80 ± 0.61 |  |  |  |
| DG(32:1) | Yg | 9.63 ± 1.15^a^ | 8.10 ± 1.81^ab^ | 5.43 ± 0.77^b^ | 10^3^ | <0.01 | 0.11 |
|  | Old | 13.81 ± 2.25^d^ | 5.72 ± 0.79^e^ | 7.74 ± 2.29^de^ |  |  |  |
| DG(34:0) | Yg | 7.92 ± 1.13^a,d^ | 4.03 ± 1.01^ab,e^ | 3.51 ± 0.32^b,de,+^ | 10^3^ | <0.01 | 0.44 |
|  | Old | 8.54 ± 1.82 | 3.59 ± 0.74 | 6.01 ± 1.56^+^ |  |  |  |
| DG(34:1) | Yg | 1.45 ± 0.21 | 1.84 ± 0.34 | 2.21 ± 0.17 | 10^4^ | <0.01 | 0.45 |
|  | Old | 1.28 ± 0.13^d^ | 1.39 ± 0.13^de^ | 2.43 ± 0.49^e^ |  |  |  |
| DG(34:2) | Yg | 1.08 ± 0.10^a,d^ | 2.08 ± 0.34^b,de^ | 1.90 ± 0.28^ab,e^ | 10^4^ | <0.01 | 0.37 |
|  | Old | 1.08 ± 0.16 | 1.73 ± 0.21 | 2.37 ± 0.60 |  |  |  |
| DG(34:3) | Yg | 5.42 ± 0.47 | 6.25 ± 1.40 | 7.73 ± 1.40 | 10^3^ | 0.02 | 0.43 |
|  | Old | 5.67 ± 1.17 | 5.71 ± 0.87 | 10.25 ± 2.14 |  |  |  |
| DG(36:2) | Yg | 2.51 ± 0.29 | 3.65 ± 0.51 | 3.78 ± 0.48 | 10^4^ | 0.01 | 0.82 |
|  | Old | 1.81 ± 4.64 | 2.87 ± 0.30 | 3.57 ± 0.78 |  |  |  |
| DG(36:2) | Yg | 5.62 ± 0.86^a,d^ | 10.97 ± 2.29^b,de^ | 11.49 ± 1.67^ab,e^ | 10^3^ | <0.01 | 0.46 |
|  | Old | 6.50 ± 2.55^d^ | 8.70 ± 0.72^de^ | 12.95 ± 1.54^e^ |  |  |  |
|  |  |  |  |  |  |  |  |
|  |  |  |  |  |  |  |  |
| DG(36:3) | Yg | 1.72 ± 0.16^a^ | 2.00 ± 0.37^ab^ | 3.18 ± 0.46^b^ | 10^4^ | <0.001 | 0.42 |
|  | Old | 1.57 ± 0.43^d^ | 1.54 ± 0.28^de^ | 3.71 ± 0.72^e^ |  |  |  |
| DG(36:4) | Yg | 5.69 ± 0.57^a^ | 5.22 ± 0.93^a^ | 16.16 ± 2.50^b^ | 10^3^ | <0.001 | 0.68 |
|  | Old | 7.41 ± 2.81^ab,d^ | 4.45 ± 0.70^a,de^ | 18.84 ± 3.27^b,e^ |  |  |  |
| DG(38:4) | Yg | 1.54 ± 0.26 | 1.49 ± 0.23 | 3.10 ± 0.58 | 10^4^ | 0.03 | 0.86 |
|  | Old | 1.47 ± 0.89 | 1.57 ± 0.18 | 2.61 ± 0.56 |  |  |  |
| DG(42:10) | Yg | 1.61 ± 0.26^a^ | 2.00 ± 0.46^a^ | 5.84 ± 0.98^b^ | 10^3^ | <0.001 | 0.19 |
|  | Old | 2.36 ± 0.99^a^ | 1.42 ± 0.40^a^ | 8.28 ± 0.16^b^ |  |  |  |
| lysoPC(16:1) | Yg | 3.39 ± 0.95^a^ | 8.81 ± 3.25^ab^ | 15.79 ± 2.40^b^ | 10^3^ | 0.02 | 0.27 |
|  | Old | 9.87 ± 6.85 | 2.97 ± 1.49 | 15.91 ± 5.12 |  |  |  |
| lysoPC(16:1) | Yg | 4.30 ± 0.92^d^ | 1.95 ± 0.64^de^ | 1.33 ± 0.27^e^ | 10^4^ | <0.01 | 0.91 |
|  | Old | 3.90 ± 0.74^d^ | 1.44 ± 0.34^e^ | 1.16 ± 0.41^e^ |  |  |  |
| lysoPC(18:0) | Yg | 1.46 ± 0.32^a,d^ | 4.16 ± 0.90^a,e^ | 9.07 ± 2.01^b,de^ | 10^4^ | <0.001 | 0.15 |
|  | Old | 2.16 ± 1.07 | 3.67 ± 0.84 | 5.43 ± 1.46 |  |  |  |
| lysoPC(18:1) | Yg | 2.08 ± 0.67^a^ | 4.44 ± 1.70^a^ | 13.18 ± 1.55^b^ | 10^4^ | <0.001 | 0.19 |
|  | Old | 3.70 ± 2.62 | 2.36 ± 1.23 | 8.04 ± 3.00 |  |  |  |
| lysoPC(18:2) | Yg | 1.55 ± 0.56^a^ | 5.57 ± 2.16^ab^ | 8.10 ± 1.49^b^ | 10^4^ | 0.03 | 0.44 |
|  | Old | 2.34 ± 1.76 | 2.62 ± 1.27 | 5.26 ± 2.63 |  |  |  |
| lysoPC(18:3) | Yg | 1.58 ± 0.22^a^ | 2.54 ± 0.76^ab^ | 4.60 ± 0.37^b^ | 10^3^ | <0.001 | 0.21 |
|  | Old | 2.59 ± 0.99 | 1.12 ± 0.23 | 6.04 ± 1.81 |  |  |  |
| lysoPC(20:4) | Yg | 8.86 ± 1.70^a^ | 28.57 ± 10.00^a^ | 179.90 ± 31.61^b^ | 10^2^ | <0.001 | 0.29 |
|  | Old | 20.62 ± 13.63 | 10.97 ± 4.78 | 117.51 ± 46.02 |  |  |  |
| lysoPE(18:1) | Yg | 6.40 ± 1.02^d^ | 11.33 ± 4.20^de^ | 16.09 ± 2.89^e^ | 10^3^ | 0.01 | 0.44 |
|  | Old | 7.53 ± 2.74 | 5.65 ± 2.38 | 14.63 ± 3.02 |  |  |  |
| MG(18:3) | Yg | 24.07 ± 4.01^a^ | 20.93 ± 7.48^ab^ | 9.06 ± 1.80^b^ | 10^3^ | 0.01 | 0.11 |
|  | Old | 26.73 ± 3.61^a,d^ | 7.74 ± 2.24^b,de^ | 13.83 ± 4.48^ab,e^ |  |  |  |
| PC(O-15:0) | Yg | 0.78 ± 0.13^a^ | 1.22 ± 0.38^a^ | 2.65 ± 0.32^b^ | 10^3^ | <0.001 | 0.70 |
|  | Old | 0.74 ± 0.22 | 0.57 ± 0.30 | 0.26 ± 0.82 |  |  |  |
| PC(O-16:0) | Yg | 3.77 ± 0.83^a^ | 7.84 ± 2.73^a^ | 26.74 ± 3.16^b,*^ | 10^4^ | <0.001 | <0.01 |
|  | Old | 8.08 ± 4.29 | 4.76 ± 1.37 | 12.34 ± 4.71^*^ |  |  |  |
| PC(O-30:0) | Yg | 2.19 ± 0.29^a,de^ | 3.65 ± 0.20^b,d^ | 5.22 ± 0.67^b,e^ | 10^3^ | 0.01 | 0.83 |
|  | Old | 2.86 ± 1.51 | 3.80 ± 0.24 | 5.01 ± 1.03 |  |  |  |
| PC(30:1) | Yg | 5.49 ± 1.23^a^ | 17.13 ± 2.24^b^ | 18.30 ± 1.40^b^ | 10^3^ | <0.001 | 0.48 |
|  | Old | 7.08 ± 2.76^d^ | 14.06 ± 2.24^e^ | 16.23 ± 3.06^de^ |  |  |  |
| PC(32:0) | Yg | 6.48 ± 1.40^a^ | 25.55 ± 1.69^b^ | 41.01 ± 6.38^b^ | 10^4^ | <0.001 | 0.71 |
|  | Old | 14.67 ± 7.90 | 28.35 ± 3.15 | 40.68 ± 6.29 |  |  |  |
| PC(34:0) | Yg | 7.45 ± 1.30 | 10.35 ± 1.17 | 14.00 ± 2.32 | 10^4^ | 0.01 | 0.84 |
|  | Old | 5.29 ± 3.24 | 10.78 ± 1.56 | 13.93 ± 2.49 |  |  |  |
| PC(34:1) | Yg | 7.90 ± 2.06^a^ | 8.68 ± 1.04^a^ | 15.57 ± 0.57^b^ | 10^5^ | <0.001 | 0.44 |
|  | Old | 4.89 ± 2.71^a^ | 8.91 ± 0.69^a^ | 15.39 ± 0.57^b^ |  |  |  |
| PC(34:2) | Yg | 4.27 ± 0.98^a^ | 8.59 ± 0.74^b^ | 7.77 ± 1.04^ab^ | 10^5^ | <0.001 | 0.60 |
|  | Old | 2.87 ± 1.83 | 9.40 ± 1.16 | 8.72 ± 1.14 |  |  |  |
| PC(34:3) | Yg | 1.22 ± 0.20^a^ | 3.59 ± 0.65^b^ | 2.23 ± 0.72^ab^ | 10^4^ | 0.02 | 0.94 |
|  | Old | 1.92 ± 1.36 | 4.61 ± 0.89 | 2.61 ± 0.69 |  |  |  |
| PC(O-34:2) | Yg | 9.71 ± 1.32^a^ | 37.15 ± 5.39^b^ | 24.85 ± 4.18^c^ | 10^3^ | <0.001 | 0.33 |
|  | Old | 12.70 ± 5.71 | 30.02 ± 1.05 | 28.55 ± 6.62 |  |  |  |
| PC(35:3) | Yg | 4.70 ± 0.57^a,d^ | 8.56 ± 0.90^b,de^ | 9.67 ± 1.59^ab,e^ | 10^3^ | 0.04 | 0.72 |
|  | Old | 6.39 ± 2.73 | 8.88 ± 0.80 | 8.93 ± 1.49 |  |  |  |
| PC(35:4) | Yg | 8.69 ± 0.87 | 7.04 ± 0.61 | 15.58 ± 3.31 | 10^2^ | <0.01 | 0.80 |
|  | Old | 10.01 ± 1.86^ab^ | 6.58 ± 0.43^a^ | 16.93 ± 2.74^b^ |  |  |  |
| PC(36:1) | Yg | 11.87 ± 2.55 | 20.80 ± 2.49 | 20.64 ± 2.71 | 10^4^ | <0.001 | 0.29 |
|  | Old | 6.31 ± 2.47^a^ | 24.31 ± 2.78^b^ | 19.27 ± 3.06^ab^ |  |  |  |
| PC(36:2) | Yg | 3.14 ± 0.80^a^ | 10.50 ± 0.91^b^ | 6.62 ± 0.53^c^ | 10^5^ | <0.001 | 0.43 |
|  | Old | 1.45 ± 0.69^a^ | 10.98 ± 1.07^b^ | 6.31 ± 0.72^c^ |  |  |  |
| PC(36:2) | Yg | 0.26 ± 0.05 | 10.57 ± 3.95 | 3.64 ± 1.99 | 10^3^ | 0.01 | 0.48 |
|  | Old | 0.24 ± 0.08 | 18.20 ± 9.30 | 5.05 ± 2.17 |  |  |  |
| PC(36:3) | Yg | 1.29 ± 0.25^a^ | 4.51 ± 0.57^b^ | 3.54 ± 0.70^ab^ | 10^5^ | <0.001 | 0.99 |
|  | Old | 1.34 ± 0.91 | 4.47 ± 0.52 | 3.45 ± 0.77 |  |  |  |
| PC(O-36:3) | Yg | 6.37 ± 0.95^a^ | 25.97 ± 3.74^b^ | 21.56 ± 3.31^b^ | 10^3^ | <0.001 | 0.36 |
|  | Old | 6.01 ± 2.72^a,d^ | 21.09 ± 2.97^b,de^ | 24.95 ± 5.36^ab,e^ |  |  |  |
| PC(36:4) | Yg | 3.83 ± 0.85 | 4.49 ± 0.67 | 11.45 ± 2.72 | 10^4^ | <0.001 | 0.55 |
|  | Old | 2.97 ± 1.64^ab^ | 5.25 ± 0.91^a^ | 14.34 ± 3.01^b^ |  |  |  |
| PC(36:4) | Yg | 3.27 ± 0.66 | 5.65 ± 1.08 | 8.03 ± 1.62 | 10^4^ | 0.01 | 0.94 |
|  | Old | 3.23 ± 2.11 | 6.44 ± 1.15 | 9.08 ± 2.09 |  |  |  |
| PC(36:4) | Yg | 1.30 ± 0.30^a^ | 7.23 ± 1.49^b^ | 1.96 ± 0.35^a^ | 10^4^ | <0.001 | 0.87 |
|  | Old | 1.75 ± 1.29^ab^ | 6.87 ± 1.37^a^ | 2.65 ± 0.66^b^ |  |  |  |
| PC(36:5) | Yg | 2.63 ± 0.38^d^ | 6.12 ± 1.09^e^ | 3.85 ± 1.02^de^ | 10^4^ | 0.01 | 0.86 |
|  | Old | 2.42 ± 1.23 | 6.80 ± 1.33 | 4.78 ± 1.24 |  |  |  |
| PC(38:3) | Yg | 1.76 ± 0.25^a^ | 3.98 ± 0.37^b^ | 4.99 ± 0.59^b^ | 10^4^ | <0.001 | 0.77 |
|  | Old | 1.62 ± 0.61^d^ | 4.41 ± 0.67^de^ | 5.71 ± 0.92^e^ |  |  |  |
| PC(38:4) | Yg | 3.77 ± 0.89^d^ | 7.00 ± 1.08^de^ | 9.44 ± 1.49^e^ | 10^4^ | <0.001 | 0.56 |
|  | Old | 2.10 ± 0.85^d^ | 7.56 ± 1.26^de^ | 10.45 ± 2.14^e^ |  |  |  |
| PC(38:5) | Yg | 2.65 ± 0.47^d^ | 3.79 ± 0.53^de^ | 8.80 ± 2.05^e^ | 10^4^ | <0.01 | 0.74 |
|  | Old | 2.49 ± 1.11^ab^ | 4.12 ± 0.79^a^ | 10.32 ± 2.47^b^ |  |  |  |
| PC(38:6) | Yg | 10.10 ± 1.44^a^ | 39.24 ± 9.03^b^ | 23.02 ± 6.66^ab^ | 10^3^ | <0.01 | 0.90 |
|  | Old | 9.77 ± 6.55^de^ | 36.40 ± 9.08^d^ | 26.51 ± 8.03^e^ |  |  |  |
|  |  |  |  |  |  |  |  |
|  |  |  |  |  |  |  |  |
| PC(38:7) | Yg | 8.15 ± 1.73 | 6.40 ± 1.14 | 4.69 ± 0.64 | 10^3^ | 0.01 | 0.49 |
|  | Old | 9.22 ± 2.00^de^ | 7.59 ± 1.60^d^ | 5.66 ± 0.96^e^ |  |  |  |
| PC(P-39:1) | Yg | 6.09 ± 1.15^a^ | 11.63 ± 2.34^b^ | 16.98 ± 2.52^b^ | 10^4^ | <0.001 | 0.87 |
|  | Old | 5.50 ± 3.04 | 8.87 ± 0.58 | 14.67 ± 2.81 |  |  |  |
| PE(36:1) | Yg | 1.52 ± 0.24^a^ | 2.83 ± 0.22^b^ | 3.57 ± 0.65^ab^ | 10^4^ | 0.01 | 0.88 |
|  | Old | 1.23 ± 0.69 | 2.79 ± 0.33 | 3.65 ± 0.68 |  |  |  |
| PE(P-36:2) | Yg | 8.89 ± 1.78^a,d^ | 16.49 ± 3.58^ab,e^ | 22.14 ± 3.01^b,de^ | 10^3^ | <0.001 | 0.91 |
|  | Old | 6.84 ± 2.24 | 16.48 ± 2.60 | 22.43 ± 6.20 |  |  |  |
| PE(36:3) | Yg | 3.12 ± 0.53^a^ | 6.67 ± 0.91^b^ | 3.47 ± 0.42^a^ | 10^4^ | <0.001 | 0.35 |
|  | Old | 1.86 ± 0.62^a,de^ | 7.11 ± 0.91^b,d^ | 4.15 ± 0.88^ab,e^ |  |  |  |
| PE(38:1) | Yg | 11.31 ± 2.01^a^ | 16.65 ± 1.97^a^ | 27.67 ± 3.09^b^ | 10^3^ | <0.001 | 0.68 |
|  | Old | 8.60 ± 2.82^d^ | 17.68 ± 1.81^d^ | 26.36 ± 4.15^e^ |  |  |  |
| PE(38:2) | Yg | 10.00 ± 1.46^a,d^ | 26.58 ± 3.37^b,de^ | 22.47 ± 3.84^ab,e^ | 10^3^ | <0.001 | 0.94 |
|  | Old | 9.72 ± 4.94 | 28.82 ± 3.99 | 23.98 ± 4.71 |  |  |  |
| PE(P-38:4) | Yg | 2.62 ± 0.59 | 2.09 ± 0.48 | 3.97 ± 0.62 | 10^4^ | 0.01 | 0.57 |
|  | Old | 1.59 ± 0.51 | 2.31 ± 0.70 | 4.08 ± 1.11 |  |  |  |
| PE(42:2) | Yg | 6.36 ± 1.28^a^ | 12.64 ± 2.87^ab^ | 18.97 ± 2.18^b^ | 10^3^ | 0.02 | 0.46 |
|  | Old | 12.75 ± 9.92 | 8.62 ± 1.27 | 25.13 ± 6.23 |  |  |  |
| PS(30:1) | Yg | 25.63 ± 5.01 | 40.00 ± 8.67 | 12.83 ± 3.25 | 10^2^ | 0.03 | 0.72 |
|  | Old | 25.37 ± 7.70 | 29.75 ± 14.17 | 8.57 ± 1.47 |  |  |  |
| SM(d32:1) | Yg | 2.31 ± 0.38^a^ | 6.68 ± 0.53^b^ | 7.97 ± 0.70^b^ | 10^3^ | <0.001 | 0.32 |
|  | Old | 3.49 ± 1.18^a^ | 6.72 ± 1.03^b^ | 7.20 ± 0.76^ab^ |  |  |  |
| SM(d34:1) | Yg | 4.36 ± 0.81^a^ | 33.01 ± 6.31^b^ | 47.17 ± 9.16^b^ | 10^4^ | <0.001 | 0.91 |
|  | Old | 5.53 ± 2.96^a,d^ | 28.90 ± 10.29^ab,e^ | 46.16 ± 12.76^b,d^ |  |  |  |
| SM(d38:1) | Yg | 3.76 ± 0.87 | 8.91 ± 1.82 | 9.08 ± 1.86 | 10^4^ | 0.01 | 0.88 |
|  | Old | 2.54 ± 1.32^d^ | 6.34 ± 1.05^e^ | 7.95 ± 1.78^de^ |  |  |  |
| TG(48:1) | Yg | 3.31 ± 0.38^a,de,+^ | 2.40 ± 0.31^ab,d^ | 1.57 ± 0.19^b,E^ | 10^4^ | 0.02 | 0.11 |
|  | Old | 8.58 ± 2.85^+^ | 2.62 ± 0.56 | 2.34 ± 0.85 |  |  |  |
| TG(49:1) | Yg | 18.33 ± 3.30^a,+^ | 12.31 ± 1.98^a^ | 5.74 ± 0.67^b^ | 10^3^ | 0.02 | 0.12 |
|  | Old | 54.01 ± 18.96^+^ | 14.69 ± 4.60 | 11.21 ± 5.25 |  |  |  |
| TG(49:2) | Yg | 23.70 ± 4.13^a,de,*^ | 14.33 ± 2.49^ab,d^ | 6.66 ± 0.87^b,e^ | 10^3^ | <0.01 | 0.05 |
|  | Old | 71.58 ± 21.93^*^ | 14.12 ± 3.99 | 10.21 ± 4.68 |  |  |  |
| TG(51:2) | Yg | 22.64 ± 4.15^+^ | 13.19 ± 1.71 | 9.98 ± 1.13 | 10^3^ | 0.01 | 0.17 |
|  | Old | 51.84 ± 15.76^+^ | 13.23 ± 2.83 | 12.91 ± 4.45 |  |  |  |
| TG(52:5) | Yg | 9.59 ± 1.44 | 20.15 ± 3.99^*^ | 19.76 ± 6.90 | 10^3^ | 0.01 | 0.21 |
|  | Old | 12.68 ± 2.95 | 43.73 ± 10.73^*^ | 27.92 ± 7.21 |  |  |  |
| TG(54:4) | Yg | 5.42 ± 0.91 | 7.57 ± 0.82 | 8.04 ± 1.19 | 10^4^ | <0.001 | 0.16 |
|  | Old | 3.52 ± 0.86^a^ | 9.93 ± 1.39^b^ | 9.55 ± 1.74^b^ |  |  |  |
| TG(54:5) | Yg | 5.60 ± 1.36 | 10.24 ± 0.22 | 11.76 ± 3.72 | 10^4^ | <0.001 | 0.30 |
|  | Old | 3.74 ± 1.48^a,d^ | 17.31 ± 3.50^b,de^ | 15.98 ± 3.84^ab,e^ |  |  |  |
| TG(54:7) | Yg | 8.41 ± 1.36 | 11.41 ± 2.45 | 13.49 ± 3.73 | 10^3^ | 0.04 | 0.40 |
|  | Old | 9.20 ± 1.99 | 20.79 ± 5.72 | 18.73 ± 3.46 |  |  |  |
| TG(55:2) | Yg | 6.46 ± 1.20 | 13.20 ± 2.17 | 19.26 ± 4.83 | 10^4^ | <0.001 | 0.36 |
|  | Old | 4.55 ± 1.14^a,d^ | 20.02 ± 3.54^b,de^ | 27.67 ± 7.59^ab,e^ |  |  |  |
| TG(56:4) | Yg | 4.69 ± 0.73^d^ | 8.62 ± 1.46^de^ | 15.32 ± 3.36^e^ | 10^3^ | <0.01 | 0.57 |
|  | Old | 5.40 ± 1.18^d^ | 12.55 ± 2.23^de^ | 21.08 ± 5.09^e^ |  |  |  |
| TG(56:5) | Yg | 9.36 ± 2.03^a,d^ | 20.54 ± 3.01^a,e,*^ | 45.26 ± 7.28^b,de^ | 10^3^ | <0.001 | 0.38 |
|  | Old | 11.53 ± 5.10^a,de^ | 30.74 ± 3.54^ab,d,*^ | 61.67 ± 8.85^b,e^ |  |  |  |
| TG(56:7) | Yg | 8.39 ± 1.50^a,d^ | 18.24 ± 3.16^ab,e,+^ | 56.98 ± 12.94^b,de^ | 10^3^ | <0.001 | 0.35 |
|  | Old | 10.94 ± 5.35^a,de^ | 31.36 ± 6.34^ab,d,+^ | 84.98 ± 19.53^b,e^ |  |  |  |
| TG(56:8) | Yg | 4.41 ± 0.84 | 11.94 ± 3.52 | 21.15 ± 6.47 | 10^3^ | <0.001 | 0.63 |
|  | Old | 7.40 ± 3.47^d^ | 20.71 ± 3.49^de^ | 32.28 ± 6.50^e^ |  |  |  |
| TG(58:5) | Yg | 1.99 ± 0.24^d,*^ | 3.02 ± 0.27^de^ | 5.80 ± 1.25^e^ | 10^3^ | 0.01 | 0.93 |
|  | Old | 3.09 ± 0.43^*^ | 3.97 ± 0.65 | 7.12 ± 1.94 |  |  |  |
| TG(58:6) | Yg | 2.02 ± 0.36^a,de^ | 5.27 ± 1.07^ab,d,+^ | 14.48 ± 3.78^b,e^ | 10^3^ | <0.001 | 0.57 |
|  | Old | 4.06 ± 1.87^d^ | 8.17 ± 1.19^de,+^ | 21.15 ± 5.17^e^ |  |  |  |
| TG(58:8) | Yg | 1.82 ± 0.38 | 2.48 ± 0.34^*^ | 2.94 ± 0.52 | 10^4^ | <0.001 | 0.09 |
|  | Old | 1.55 ± 0.45^a,d^ | 4.18 ± 0.70^*ab,e^ | 4.22 ± 0.63^b,de^ |  |  |  |
| TG(58:9) | Yg | 2.00 ± 0.12^a^ | 6.20 ± 1.22^b^ | 24.16 ± 6.03^c^ | 10^3^ | <0.001 | 0.27 |
|  | Old | 3.62 ± 1.61^a^ | 7.98 ± 1.04^a^ | 36.26 ± 7.03^b^ |  |  |  |
| TG(62:14) | Yg | 1.15 ± 0.15^d,+^ | 1.53 ± 0.24^d^ | 4.08 ± 1.04^e^ | 10^3^ | <0.001 | 0.52 |
|  | Old | 2.37 ± 0.65^ab,+^ | 1.97 ± 0.38^a^ | 5.86 ± 1.20^b^ |  |  |  |
| Subclass: Ceramides and related molecules | | | |  |  |  |  |
| C18 Ceramide (d18:1/18:0) | Yg | 5.47 ± 0.90^d^ | 11.87 ± 1.83^e^ | 7.94 ± 1.80^de^ | 10^3^ | 0.01 | 0.46 |
|  | Old | 4.04 ± 0.51 | 9.73 ± 2.78 | 9.79 ± 2.31 |  |  |  |
| C20 Ceramide (d18:1/20:0) | Yg | 3.37 ± 0.62^a^ | 6.07 ± 1.07^b^ | 4.65 ± 1.09^ab^ | 10^3^ | 0.02 | 0.41 |
|  | Old | 3.00 ± 0.90 | 5.23 ± 1.36 | 6.10 ± 1.20 |  |  |  |
| C22 Ceramide (d18:1/22:0) | Yg | 5.33 ± 0.82 | 5.25 ± 1.10 | 7.87 ± 1.48 | 10^3^ | 0.01 | 0.62 |
|  | Old | 3.58 ± 0.62^d^ | 5.53 ± 1.41^de^ | 7.44 ± 1.16^e^ |  |  |  |
| C24:1 Ceramide (d18:1/24:1(15Z)) | Yg | 4.85 ± 0.80^a^ | 9.52 ± 2.64^ab^ | 12.22 ± 1.78^b^ | 10^3^ | <0.01 | 0.65 |
|  | Old | 5.17 ± 2.62 | 8.33 ± 1.90 | 15.06 ± 3.30 |  |  |  |
| C24:1 Ceramide (d18:1/24:1(15Z)) | Yg | 3.73 ± 0.88 | 2.34 ± 0.71 | 2.49 ± 0.19 | 10^2^ | 0.04 | 0.41 |
|  | Old | 3.61 ± 0.60^d^ | 1.31 ± 0.33^e^ | 3.31 ± 1.16^de^ |  |  |  |
| Cer(24:0-OH) | Yg | 4.55 ± 0.70^d^ | 2.40 ± 0.98^de^ | 1.73 ± 0.34^e^ | 10^5^ | <0.01 | 0.81 |
|  | Old | 3.78 ± 0.78 | 1.38 ± 0.29 | 1.58 ± 0.60 |  |  |  |
| Cer(d34:1) | Yg | 5.69 ±1.26^a^ | 36.07 ± 3.83^b^ | 16.48 ± 2.36^c^ | 10^3^ | <0.001 | 0.29 |
|  | Old | 4.51 ± 1.33^a^ | 30.68 ± 1.61^b^ | 21.43 ± 6.30^ab^ |  |  |  |
| Cer(d34:0) | Yg | 2.78 ± 0.32^a^ | 2.74 ± 0.38^a^ | 1.52 ± 0.11^b,+^ | 10^3^ | 0.01 | 0.12 |
|  | Old | 2.67 ± 0.40 | 1.92 ± 0.24 | 1.98 ± 0.26^+^ |  |  |  |
| Cer(d40:2) | Yg | 1.55 ± 0.23^a^ | 2.84 ± 0.34^b^ | 2.37 ± 0.20^b^ | 10^3^ | 0.01 | 0.08 |
|  | Old | 1.77 ± 0.31 | 2.09 ± 0.30 | 3.10 ± 0.66 |  |  |  |
| GlcCer(d34:1) | Yg | 8.15 ± 1.73 | 6.40 ± 1.14 | 4.69 ± 0.64 | 10^3^ | 0.02 | 1.00 |
|  | Old | 9.22 ± 2.00 | 7.59 ± 1.60 | 5.66 ± 0.96 |  |  |  |
| Amino Acids and Derivatives | | | |  |  |  |  |
| α‐ketobutyrate | Yg | 1.57 ± 0.18^a^ | 22.20 ± 3.37^b^ | 34.26 ± 6.20^b^ | 10^3^ | <0.001 | 0.24 |
|  | Old | 1.95 ± 0.23^a^ | 18.60 ± 3.39^b^ | 21.17 ± 3.10^b^ |  |  |  |
| Alanine (2TMS) | Yg | 12.11 ± 0.93^a^ | 8.09 ± 1.36^a^ | 31.54 ± 3.52^b,+^ | 10^6^ | <0.001 | 0.04 |
|  | Old | 11.59 ± 2.27^ab^ | 8.33 ± 1.50^a^ | 21.69 ± 2.27^b,+^ |  |  |  |
| Cysteine (3TMS) | Yg | 1.75 ± 0.27^ab,d^ | 1.43 ± 0.16^a,de^ | 5.04 ± 1.03^b,e^ | 10^5^ | 0.01 | 0.45 |
|  | Old | 1.29 ± 0.11 | 1.55 ± 0.38 | 7.36 ± 3.35 |  |  |  |
| Cysteinyl-Proline | Yg | 5.38 ± 2.38 | 2.65 ± 1.24 | 140.09 ± 55.80 | 10^2^ | 0.01 | 0.31 |
|  | Old | 6.78 ± 2.74^a^ | 3.42 ± 1.15^a^ | 70.54 ± 18.28^b^ |  |  |  |
| ɛ-polylysine | Yg | 54.52 ± 26.91^de^ | 103.39 ± 29.28^d^ | 9.41 ± 4.83^e^ | 10^3^ | <0.01 | 0.86 |
|  | Old | 38.11 ± 23.96 | 89.00 ± 21.74 | 15.32 ± 13.03 |  |  |  |
| Glutamic acid (3TMS) | Yg | 5.22 ± 0.44^a,*^ | 7.36 ± 0.93^a,*^ | 85.24 ± 9.21^b^ | 10^5^ | <0.001 | 0.76 |
|  | Old | 11.36 ± 3.08^a,de,*^ | 15.20 ± 3.27^b,d,*^ | 85.80 ± 21.33^b,e^ |  |  |  |
| Glycine (3TMS) | Yg | 2.84 ± 0.12^a^ | 2.35 ± 0.16^a,+^ | 7.71 ± 0.54^b^ | 10^7^ | <0.001 | 0.07 |
|  | Old | 2.81 ± 0.10^a^ | 3.12 ± 0.42^a,+^ | 6.39 ± 0.79^b^ |  |  |  |
| Glycine (2TMS) | Yg | 6.76 ± 0.54^a,*^ | 6.69 ± 0.32^a^ | 2.70 ± 0.42^b^ | 10^7^ | <0.001 | 0.20 |
|  | Old | 4.97 ± 0.62^ab,d,*^ | 5.25 ± 0.84^a,de^ | 2.61 ± 0.76^b,e^ |  |  |  |
| Glycyl-Tyrosine | Yg | 71.47 ± 26.08^ab^ | 98.31 ± 20.53^a^ | 4.44 ± 1.90^b^ | 10^4^ | <0.001 | 0.47 |
|  | Old | 45.70 ± 21.6^a^ | 116.89 ± 15.65^b^ | 25.70 ± 23.36^ab^ |  |  |  |
| Pyroglutamic acid (2TMS) | Yg | 5.32 ± 1.67^a^ | 5.49 ± 0.70^a^ | 91.45 ± 4.37^b^ | 10^6^ | <0.001 | 0.18 |
|  | Old | 5.49 ± 0.72^a^ | 18.53 ± 9.52^a^ | 81.82 ± 16.20^b^ |  |  |  |
| Serine (3TMS) | Yg | 2.92 ± 0.86^a^ | 1.09 ± 0.37^a^ | 7.90 ± 0.53^b^ | 10^6^ | 0.01 | 0.32 |
|  | Old | 3.02 ± 1.31 | 4.16 ± 3.30 | 6.93 ± 1.21 |  |  |  |
| Threonine (3TMS) | Yg | 4.82 ± 1.39^a^ | 3.05 ± 0.35^a^ | 50.94 ± 4.69^b^ | 10^5^ | <0.001 | 0.17 |
|  | Old | 3.60 ± 0.65^a^ | 7.80 ± 4.07^a^ | 40.51 ± 8.34^b^ |  |  |  |
| Miscellaneous |  |  |  |  |  |  |  |
| 2,2,4,4,6,6-xxx-trithiane^8^ | Yg | 22.15 ± 6.66 | 16.05 ± 3.46^+^ | 14.44 ± 4.48 | 10^3^ | 0.01 | 0.23 |
|  | Old | 30.28 ± 10.49 | 7.12 ± 2.75^+^ | 5.68 ± 1.80 |  |  |  |
| 2,2,4,4,6,6-xxx-trithiane^8^ | Yg | 24.88 ± 4.78^a^ | 14.26 ± 2.87^b^ | 14.25 ± 4.90^ab^ | 10^3^ | 0.01 | 0.66 |
|  | Old | 25.44 ± 7.90 | 8.24 ± 2.91 | 6.40 ± 0.93 |  |  |  |
| (2R)-2-xxx- heptadecanoate^9^ | Yg | 2.96 ± 0.42^a^ | 3.70 ± 0.69^a^ | 13.03 ± 2.02^b^ | 10^3^ | <0.001 | 0.41 |
|  | Old | 4.39 ± 1.14^d^ | 2.91 ± 0.77^d^ | 16.58 ± 4.09^e^ |  |  |  |
|  |  |  |  |  |  |  |  |
|  |  |  |  |  |  |  |  |
| 2-(4-methylthiazol-5-yl)ethyl acetate | Yg | 21.17 ± 2.77^d^ | 10.01 ± 2.33^e^ | 8.43 ± 1.80^e^ | 10^4^ | <0.01 | 0.83 |
|  | Old | 17.52 ± 3.16 | 7.33 ± 1.72 | 6.89 ± 2.51 |  |  |  |
| 3-xxx-furanone^10^ | Yg | 3.04 ± 0.60^d,+^ | 1.43 ± 0.17^de^ | 1.21 ± 0.17^e^ | 10^3^ | <0.001 | 0.1 |
|  | Old | 5.13 ± 0.90^a,d,+^ | 1.26 ± 0.15^b,de^ | 1.31 ± 0.41^ab,e^ |  |  |  |
| (3aS,5aS)-xxx- chrysene^11^ | Yg | 6.29 ± 1.04^a^ | 29.20 ± 4.21^b^ | 13.26 ± 3.79^a^ | 10^3^ | 0.01 | 0.67 |
|  | Old | 9.79 ± 3.57 | 26.54 ± 13.86 | 9.34 ± 3.00 |  |  |  |
| (3beta)-xxx-oic acid^12^ | Yg | 3.80 ± 0.70 | 7.80 ± 2.43 | 28.15 ± 10.75 | 10^2^ | 0.01 | 0.62 |
|  | Old | 3.83 ± 1.76 | 10.01 ± 2.50 | 38.07 ± 11.76 |  |  |  |
| 3-xxx (-9-hexadecenoate)^13^ | Yg | 10.68 ± 1.55^a,*^ | 6.26 ± 1.15^b^ | 3.56 ± 0.54^b^ | 10^3^ | <0.001 | 0.06 |
|  | Old | 21.24 ± 5.11^d,*^ | 6.85 ± 1.15^e^ | 4.12 ± 1.33^e^ |  |  |  |
| (5,5-xxx)methyl methanesulfonate^14^ | Yg | 30.28 ± 6.48^d^ | 20.39 ± 4.81^e,+^ | 18.77 ± 6.58^de^ | 10^3^ | 0.02 | 0.42 |
|  | Old | 34.55 ± 12.78 | 8.76 ± 3.09^+^ | 7.14 ± 1.75 |  |  |  |
| 9-(4-Hydroxybutyl)-N2-Phenylguanine | Yg | 5.14 ± 0.63^a^ | 2.12 ± 0.41^b,+^ | 1.47 ± 0.28^b^ | 10^4^ | <0.001 | 0.56 |
|  | Old | 3.99 ± 0.77^d^ | 1.19 ± 0.17^e,+^ | 1.07 ± 0.19^e^ |  |  |  |
| 9-(4-Hydroxybutyl)-N2-Phenylguanine | Yg | 4.91 ± 0.73^a,d^ | 2.39 ± 0.58^ab,e^ | 1.80 ± 0.40^b,de^ | 10^4^ | <0.001 | 0.72 |
|  | Old | 4.79 ± 0.91^d^ | 1.55 ± 0.34^e^ | 1.24 ± 0.35^e^ |  |  |  |
| 9-(4-Hydroxybutyl)-N2-Phenylguanine | Yg | 7.08 ± 1.22^d^ | 3.96 ± 1.05^de^ | 2.65 ± 0.56^e^ | 10^4^ | <0.01 | 0.54 |
|  | Old | 7.15 ± 1.85 | 1.99 ± 0.39 | 1.64 ± 0.39 |  |  |  |
| [(E,2S,3R)-xxx-ethyl phosphate^15^ | Yg | 9.94 ± 1.53^a^ | 83.72 ± 10.58^b^ | 95.69 ± 19.45^b^ | 10^2^ | <0.001 | 0.84 |
|  | Old | 13.03 ± 5.31^a^ | 75.29 ± 14.57^b^ | 86.27 ± 18.06^b^ |  |  |  |
| Adenosine (3TMS) (Derivate not found) | Yg | 8.95 ± 1.31^a^ | 31.35 ± 6.57^b^ | 20.29 ± 7.75^ab^ | 10^4^ | <0.001 | 0.78 |
|  | Old | 7.14 ± 0.98^a^ | 35.65 ± 7.60^b^ | 26.03 ± 8.90^a^ |  |  |  |
| Adenosine (3TMS) (Derivate not found) | Yg | 4.56 ± 0.64^a^ | 5.01 ± 0.86^a^ | 1.24 ± 0.18^b^ | 10^4^ | 0.03 | 0.65 |
|  | Old | 4.66 ± 1.13 | 7.05 ± 1.70 | 5.53 ± 2.35 |  |  |  |
| Adenosine | Yg | 1.68 ± 0.47^a,+^ | 1.91 ± 0.57^a^ | 18.87 ± 3.91^b^ | 10^3^ | <0.001 | 0.07 |
|  | Old | 4.01 ± 1.25^de,+^ | 2.63 ± 0.70^d^ | 10.78 ± 2.19^e^ |  |  |  |
| Phosphoric acid (3TMS) | Yg | 2.69 ± 0.33 | 5.11 ± 1.00 | 3.96 ± 0.64 | 10^7^ | <0.001 | 0.16 |
|  | Old | 3.12 ± 0.62^d^ | 7.74 ± 1.35^e^ | 3.65 ± 0.97^de^ |  |  |  |
| Phosphoric acid monomethyl ester^16^ | Yg | 1.07 ± 0.19^d^ | 3.30 ± 0.58^e^ | 2.75 ± 1.32^de^ | 10^6^ | 0.04 | 0.8 |
|  | Old | 1.28 ± 0.31^d^ | 3.42 ± 0.56^e^ | 3.56 ± 1.24^de^ |  |  |  |
| Putrescine (3TMS) | Yg | 23.29 ± 2.06^a^ | 12.91 ± 1.63^b,+^ | 7.09 ± 0.50^c^ | 10^5^ | 0.11 | 0.23 |
|  | Old | 105.65 ± 57.88 | 123.99 ± 70.58^+^ | 67.65 ± 42.34 |  |  |  |
| Pyridine, 2-hydroxy- (1TMS) | Yg | 4.25 ± 0.62^a^ | 4.37 ± 0.30^a^ | 2.21 ± 0.14^b^ | 10^8^ | <0.001 | 0.44 |
|  | Old | 3.62 ± 0.81^ab^ | 3.35 ± 0.70^a^ | 1.81 ± 0.57^b^ |  |  |  |
| Pyridoxamine (3TMS) | Yg | 10.30 ± 1.75^a^ | 7.38 ± 1.13^a^ | 2.17 ± 0.28^b^ | 10^5^ | <0.001 | 0.59 |
|  | Old | 8.20 ± 0.83^a^ | 6.10 ± 1.08^a^ | 2.18 ± 0.53^b^ |  |  |  |
| Sitosterol, beta- (1TMS) | Yg | 6.91 ± 1.66^ab^ | 10.09 ± 2.02^a^ | 3.98 ± 0.74^b^ | 10^3^ | <0.001 | 0.67 |
|  | Old | 6.68 ± 1.49^ab^ | 7.42 ± 1.60^a^ | 3.18 ± 0.79^b^ |  |  |  |
| Raoline | Yg | 7.36 ± 1.19 | 8.72 ± 1.25 | 10.97 ± 2.06 | 10^4^ | 0.05 | 0.45 |
|  | Old | 5.83 ± 0.43 | 12.39 ± 3.10 | 12.68 ± 2.51 |  |  |  |

^1^Inositol-2-phosphate, myo- (7TMS);^2^1-[5-(1-Hydroxytridecyl)tetrahydro-2-furanyl]-13-(5-methyl-2-oxo-2,5-dihydro-3-furanyl)tridecyl palmitate; ^3^(3beta)-Cholest-5-en-3-yl (5Z,8Z,11Z,14Z)-5,8,11,14-eicosatetraenoate; ^4^3-(Palmitoyloxy)-1,2-propanediyl(9E,9'E)bis(-9-octadecenoate); ^5^4-Hydroxy-3-[(1E)-5-oxo-1,7-diphenyl-1-hepten-3-yl]-6-[(E)-2-phenylvinyl]-2H-pyran-2-one; ^6^ethyl 2-[(3S,4R,6R)-6-butyl-4,6-diethyldioxan-3-yl]acetate; ^7^Octadecadienoic acid, 9,12-(Z,Z)- (1TMS);^8^2,2,4,4,6,6-Hexamethyl-1,3,5-trithiane; ^9^(2R)-2-(Palmitoyloxy)-3-(phosphonooxy)propyl heptadecanoate; ^10^3-(13,14-Dihydroxytriacontyl)-5-methyl-2(5H)-furanone; ^11^(3aS,5aS,5bR,7aS,11aS,11bR)-3a,5a,5b,8,8,11a-hexamethyl-1,2,3,4,5,6,7,7a,9,10,11,11b,12,13-tetradecahydrocyclopenta[a]chrysene; ^12^(3beta,5xi,16alpha)-16-Hydroxy-3-{[beta-D-xylopyranosyl-(1->2)-alpha-L-arabinopyranosyl-(1->6)-2-acetamido-2-deoxy-beta-D- glucopyranosyl]oxy}olean-12-en-28-oic acid; ^13^3-(Tetradecanoyloxy)-1,2-propanediyl (9Z,9'Z)bis(-9-hexadecenoate); ^14^(5,5-Dimethyl-2-oxotetrahydro-3-furanyl)methyl methanesulfonate; ^15^[(E,2S,3R)-3-hydroxy-2-(pentadecanoylamino)octadec-4-enyl] 2-(trimethylammonio)ethyl phosphate; ^16^ Phosphoric acid monomethyl ester (2TMS)
