# Supplementary material for: Maturation and culture affect the metabolomic profile of oocytes and follicular cells in young and old mares
Source: Front Cell Dev Biol. 2024 Jan 12;11:1280998. doi: 10.3389/fcell.2023.1280998 (PMC10811030; doi:10.3389/fcell.2023.1280998)
Supplement: Supplementary file 3 [file Table3.DOCX]

Supplementary Table 3: Relative abundance of annotated metabolites in granulosa cells. Granulosa cells were collected from young mares (Yg, n=8) and old mares (Old, n=5) at 0h (GV) or 24h (MI) after maturation induction, with some granulosa cells collected at 24h and cultured for an additional 18h (MIIC). Results are presented as mean ± SEM, correction factor of 10^x^ (CF), P-values for overall effects of maturation stage (MS) and the interaction (INT) of age with maturation stage are included in the table. Different superscripts within a row represent differences at ^a,b,c^P < 0.05 or ^d,e,f^P < 0.1. Superscripts within a column for the same metabolite represent differences between Yg and Old at ^*^P < 0.05 or ^+^P < 0.1. The main effect of age was significant (P<0.05) for compounds with Yg and Old highlighted in grey. Abbreviated compound names are in the table, numerical superscripts indicate full name is at the bottom of the table.

| Class and Metabolites | Age | 0h | 24h | 42h | CF | MS | INT |
| --- | --- | --- | --- | --- | --- | --- | --- |
| Carbohydrates and derivatives | | | | |  |  |  |
| Glucose (1MEOX) (5TMS) MP | Yg | 1.97 ± 0.57^a,de*^ | 5. 78 ± 2.55^ab,d^ | 22.96 ± 3.50^b,e^ | 10^6^ | <0.001 | 0.53 |
|  | Old | 0.76 ± 0.27^a,*^ | 5.51 ± 1.00^b^ | 26.78 ± 3.76^c^ |  |  |  |
| Glucose (1MEOX) (5TMS) MP | Yg | 4.80 ± 1.35^a,*^ | 14.56 ± 5.97^a^ | 53.62 ± 4.80^b^ | 10^7^ | <0.001 | 0.45 |
|  | Old | 1.79 ± 0.56^a,*^ | 17.02 ± 3.34^b^ | 44.91 ± 5.06^c^ |  |  |  |
| Glucose (1MEOX) (5TMS) BP | Yg | 4.37 ± 1.32^a,de*^ | 11.65 ± 4.74^be,d^ | 41.60 ± 5.35^b,e^ | 10^6^ | <0.001 | 0.97 |
|  | Old | 1.84 ± 0.41^a,*^ | 10.39 ± 2.01^b^ | 38.64 ± 2.94^c^ |  |  |  |
| Inositol, myo- (6TMS) | Yg | 3.97 ± 1.43^ab,d^ | 4.48 ± 1.65^a,de^ | 29.85 ± 7.21^b,e,+^ | 10^6^ | <0.001 | 0.21 |
|  | Old | 7.30 ± 1.16^a^ | 7.75 ± 1.5^a^ | 41.60 ± 1.83^b,+^ |  |  |  |
| Inositol-2-phosphate, myo-^1^ | Yg | 10.41 ± 1.28^a^ | 7.62 ± 1.38^a^ | 2.95 ± 0.47^b,*^ | 10^5^ | <0.01 | 0.50 |
|  | Old | 14.63 ± 1.73^a^ | 13.42 ± 2.62^a^ | 4.50 ± 0.36^b,*^ |  |  |  |
| Lactic acid (2TMS) | Yg | 1.88 ± 0.29 | 1.91 ± 0.25 | 3.52 ± 0.71 | 10^8^ | <0.001 | 0.31 |
|  | Old | 2.06 ± 0.19^a^ | 2.37 ± 0.39^a^ | 5.00 ± 0.52^b^ |  |  |  |
| Sorbose (1MEOX) (5TMS) BP | Yg | 0.75 ± 0.10^a^ | 1.33 ± 0.14^a^ | 49.81 ± 5.37^b^ | 10^6^ | <0.001 | 0.93 |
|  | Old | 0.73 ± 0.09^a^ | 1.03 ± 0.19^a^ | 50.39 ± 5.97^b^ |  |  |  |
| Xylose (1MEOX) (4TMS) MP | Yg | 21.27 ± 17.82 | 22.00 ± 15.34 | 22.72 ± 13.13 | 10^5^ | 0.32 | 0.37 |
|  | Old | 4.87 ± 0.59^a^ | 37.81 ± 11.12^b^ | 30.37 ± 11.81^ab^ |  |  |  |
| Lipids and Fatty Acids | | | |  |  |  |  |
| 4-Hydroxy-xxx-pyran-2-one^2^ | Yg | 48.72 ± 9.55^a^ | 73.11 ± 6.22^b^ | 0.41 ± 0.12^c^ | 10^3^ | <0.001 | 0.51 |
|  | Old | 28.81 ± 11.52^ab^ | 73.05 ± 17.8^a^ | 0.71 ± 0.16^b^ |  |  |  |
| Cholestenone | Yg | 1.70 ± 0.18 | 2.03 ± 0.20^*^ | 3.43 ± 0.86 | 10^4^ | 0.02 | 0.62 |
|  | Old | 1.49 ± 0.18 | 1.44 ± 0.12^*^ | 2.57 ± 0.67 |  |  |  |
| Cholesterol (1TMS) | Yg | 12.94 ± 2.65^a^ | 8.86 ± 2.48^ab^ | 5.39 ± 1.68^b^ | 10^6^ | 0.04 | 0.39 |
|  | Old | 14.15 ± 1.67^a^ | 15.82 ± 3.47^ab^ | 6.44 ± 0.82^b^ |  |  |  |
| Cholesterol | Yg | 6.37 ± 0.70^a^ | 7.12 ± 0.95^ab^ | 10.34 ± 1.16^b^ | 10^3^ | <0.001 | 0.05 |
|  | Old | 6.32 ± 0.40^a^ | 5.51 ± 0.48^a^ | 13.63 ± 1.35^b^ |  |  |  |
|  |  |  |  |  |  |  |  |
|  |  |  |  |  |  |  |  |
| Linoleic acid | Yg | 7.92 ± 2.14 | 6.25 ± 1.52 | 2.74 ± 0.72 | 10^2^ | 0.02 | 0.66 |
|  | Old | 7.78 ± 1.37 | 8.59 ± 1.75 | 4.90 ± 1.31 |  |  |  |
| Glycerophosphocholine | Yg | 4.49 ± 1.03^a^ | 15.60 ± 4.79^ab,+^ | 37.33 ± 7.06^b^ | 10^3^ | <0.001 | 0.30 |
|  | Old | 7.56 ± 1.45^a^ | 8.31 ± 0.61^a,+^ | 30.80 ± 4.08^b^ |  |  |  |
| Hexadecanoic acid (1TMS) | Yg | 19.06 ± 2.48^a^ | 17.19 ± 2.26^a^ | 4.72 ± 1.00^b^ | 10^6^ | <0.001 | 0.35 |
|  | Old | 20.08 ± 1.89^a^ | 13.96 ± 1.16^b^ | 3.90 ± 0.58^c^ |  |  |  |
| Octadecanoic acid (1TMS) | Yg | 18.92 ± 3.01^a,+^ | 15.08 ± 2.65^a^ | 2.98 ± 0.64^b^ | 10^6^ | <0.001 | 0.19 |
|  | Old | 13.85 ± 1.09^a,+^ | 13.17 ± 1.30^a^ | 2.36 ± 0.44^b^ |  |  |  |
| Octadecanoic acid, 9-(E)- (1TMS) | Yg | 50.60 ± 7.21^a,de^ | 40.11 ± 9.11^ab,d^ | 8.68 ± 1.14^b,e^ | 10^5^ | <0.01 | 0.51 |
|  | Old | 60.53 ± 8.50^a^ | 63.47 ± 14.23^a^ | 10.25 ± 9.95^b^ |  |  |  |
| Octadecanoic acid, 9,12-(Z,Z)^3^ | Yg | 35.68 ± 6.11^a^ | 39.88 ± 7.88^a^ | 5.72 ± 0.95^b^ | 10^4^ | <0.01 | 0.83 |
|  | Old | 42.96 ± 8.24^a^ | 48.92 ± 12.80^a^ | 7.07 ± 0.82^b^ |  |  |  |
| Sitosterol, beta- (1TMS) | Yg | 16.38 ± 3.93^a^ | 15.87 ± 6.18^ab^ | 2.53 ± 0.97^b^ | 10^3^ | <0.001 | 0.76 |
|  | Old | 19.75 ± 4.56^a^ | 14.79 ± 2.29^a^ | 4.73 ± 0.79^b^ |  |  |  |
| CE(18:2) | Yg | 0.45 ± 0.21^a^ | 7.45 ± 3.34^ab^ | 4.96 ± 0.93^b,*^ | 10^4^ | 0.01 | 0.20 |
|  | Old | 0.34 ± 0.05^a^ | 3.35 ± 0.57^b^ | 1.82 ± 0.39^b,*^ |  |  |  |
| CE(22:5) | Yg | 2.50 ± 0.64^a,d^ | 22.93 ± 5.20^a,e^ | 96.95 ± 21.18^b,de^ | 10^2^ | <0.001 | 0.28 |
|  | Old | 6.07 ± 1.64^a,de^ | 22.21 ± 5.86^ab,d^ | 68.83 ± 14.23^b,e^ |  |  |  |
| DG(20:7) | Yg | 20.89 ± 5.27^*^ | 5.97 ± 1.47 | 19.77 ± 5.03 | 10^2^ | <0.01 | 0.17 |
|  | Old | 45.58 ± 3.56^a,d,*^ | 16.75 ± 8.24^ab,e^ | 21.96 ± 2.82^b,de^ |  |  |  |
| DG(32:0) | Yg | 15.65 ± 2.97^d^ | 4.38 ± 0.39^d^ | 7.58 ± 2.51^de^ | 10^3^ | <0.001 | 0.63 |
|  | Old | 12.43 ± 2.47^a,d^ | 4.34 ± 0.62^b,de^ | 4.89 ± 0.32^ab,e^ |  |  |  |
| DG(32:1) | Yg | 4.77 ± 0.73 | 2.63 ± 0.63 | 4.17 ± 0.43 | 10^3^ | 0.001 | 0.67 |
|  | Old | 6.23 ± 0.62^a^ | 3.87 ± 0.43^b^ | 4.73 ± 0.36^ab^ |  |  |  |
| DG(34:0) | Yg | 1.40 ± 0.14^a^ | 1.35 ± 0.23^a^ | 3.46 ± 0.14^b^ | 10^3^ | <0.001 | 0.81 |
|  | Old | 1.65 ± 0.37^a^ | 1.25 ± 0.19^a^ | 3.37 ± 0.45^b^ |  |  |  |
| DG(34:1) | Yg | 2.20 ± 0.18 | 1.37 ± 0.27 | 2.11 ± 0.15 | 10^4^ | 0.001 | 0.20 |
|  | Old | 2.15 ± 0.14^a^ | 1.84 ± 0.15^b^ | 2.15 ± 0.23^ab^ |  |  |  |
| DG(34:2) | Yg | 2.33 ± 0.31 | 1.73 ± 0.45 | 1.51 ± 0.14 | 10^4^ | <0.01 | 0.78 |
|  | Old | 2.26 ± 0.16^a^ | 1.93 ± 0.16^ab^ | 1.56 ± 0.15^b^ |  |  |  |
| DG(34:3) | Yg | 6.03 ± 1.30 | 3.10 ± 0.41 | 4.97 ± 0.54 | 10^3^ | <0.01 | 0.96 |
|  | Old | 6.37 ± 0.68^a,d^ | 3.38 ± 0.49^b,de^ | 4.91 ± 0.76^ab,e^ |  |  |  |
| DG(36:2) | Yg | 14.41 ± 2.67^a^ | 7.75 ± 2.03^b^ | 16.64 ± 2.6^a^ | 10^3^ | <0.001 | 0.81 |
|  | Old | 14.82 ± 1.52^ab,d^ | 9.70 ± 1.05^a,e^ | 19.05 ± 2.16^b,de^ |  |  |  |
| DG(36:3) | Yg | 2.42 ± 0.51^ab^ | 1.01 ± 0.18^a^ | 2.56 ± 0.29^b^ | 10^4^ | <0.001 | 0.24 |
|  | Old | 2.84 ± 0.24^a,d^ | 1.18 ± 0.18^b,de^ | 2.24 ± 0.19^a,e^ |  |  |  |
| DG(36:4) | Yg | 20.48 ± 4.74^ab,*^ | 5.37 ± 0.53^a^ | 21.83 ± 3.35^b^ | 10^3^ | <0.01 | 0.05 |
|  | Old | 43.36 ± 3.02^a,*^ | 15.23 ± 5.32^b^ | 20.55 ± 1.31^b^ |  |  |  |
| DG(36:4) | Yg | 9.79 ± 2.12 | 7.36 ± 0.84 | 11.70 ± 1.75 | 10^3^ | 0.02 | 0.90 |
|  | Old | 10.27 ± 0.83^d^ | 6.61 ± 0.82^e^ | 11.18 ± 1.53^de^ |  |  |  |
| DG(38:4) | Yg | 6.72 ± 1.60^*^ | 1.87 ± 0.40 | 5.44 ± 1.66 | 10^4^ | <0.001 | 0.13 |
|  | Old | 12.02 ± 0.77^a,*^ | 4.30 ± 1.60^b^ | 5.03 ± 0.79^b^ |  |  |  |
| DG(40:8) | Yg | 3.50 ± 0.83^a^ | 1.70 ± 0.25^a^ | 8.20 ± 1.01^b^ | 10^3^ | <0.001 | 0.15 |
|  | Old | 4.96 ± 0.61^ab,d^ | 2.38 ± 0.35^a,e^ | 6.65 ± 1.04^b,de^ |  |  |  |
| DG(42:10) | Yg | 1.20 ± 0.38^a^ | 1.66 ± 0.23^a^ | 8.47 ± 1.53^b^ | 10^3^ | <0.001 | 0.40 |
|  | Old | 1.80 ± 0.32^a^ | 1.91 ± 0.28^a^ | 6.87 ± 1.50^b^ |  |  |  |
| lysoPC(16:1) | Yg | 16.37 ± 4.82 | 4.35 ± 2.26 | 24.54 ± 10.30 | 10^3^ | 0.01 | 0.65 |
|  | Old | 29.71 ± 6.11^ab,d^ | 7.24 ± 2.58^a,e^ | 27.91 ± 10.30^b,de^ |  |  |  |
| lysoPC(18:1) | Yg | 9.23 ± 2.48 | 3.74 ± 2.04 | 10.27 ± 5.39 | 10^4^ | 0.01 | 0.49 |
|  | Old | 10.12 ± 2.08^ab^ | 3.51 ± 1.17^a^ | 17.09 ± 3.84^b^ |  |  |  |
| lysoPC(18:2) | Yg | 24.85 ± 11.35 | 36.32 ± 32.02 | 58.74 ± 36.82 | 10^3^ | 0.07 | 0.43 |
|  | Old | 35.47 ± 12.26^de^ | 8.95 ± 2.56^d^ | 95.29 ± 29.52^e^ |  |  |  |
| lysoPC(20:4) | Yg | 1.92 ± 0.71 | 1.75 ± 1.57 | 29.11 ± 17.16 | 10^3^ | 0.01 | 0.55 |
|  | Old | 6.12 ± 3.22^ab,e^ | 1.40 ± 0.35^a,de^ | 43.24 ± 10.80^b,e^ |  |  |  |
| lysoPE(18:0) | Yg | 9.42 ± 2.39^d^ | 3.59 ± 1.00^e^ | 6.74 ± 1.57^d^ | 10^3^ | 0.01 | 0.84 |
|  | Old | 12.54 ± 2.08 | 6.64 ± 1.55 | 8.00 ± 0.91 |  |  |  |
| lysoPE(18:1) | Yg | 17.62 ± 3.77 | 5.85 ± 2.35 | 13.54 ± 4.98 | 10^3^ | 0.001 | 0.81 |
|  | Old | 22.52 ± 3.15^a^ | 7.78 ± 2.59^b^ | 14.70 ± 2.58^ab^ |  |  |  |
| MG(18:3) | Yg | 3.95 ± 0.99 | 3.09 ± 1.18 | 4.00 ± 1.61 | 10^3^ | 0.04 | 0.19 |
|  | Old | 6.65 ± 1.25^a^ | 2.55 ± 0.63^b^ | 4.38 ± 0.59^ab^ |  |  |  |
| MG(20:4) | Yg | 3.95 ± 0.60 | 2.47 ± 0.45 | 7.37 ± 2.94 | 10^3^ | <0.001 | 0.82 |
|  | Old | 5.32 ± 0.98^ab^ | 2.42 ± 0.29^a^ | 8.67 ± 1.46^b^ |  |  |  |
| PC(O-15:0) | Yg | 22.37 ± 6.19 | 7.32 ± 4.21 | 30.02 ± 13.25 | 10^2^ | <0.01 | 0.56 |
|  | Old | 24.69 ± 4.66^ab,d^ | 14.86 ± 5.71^a,de^ | 47.89 ± 7.70^b,e^ |  |  |  |
| PC(28:0) | Yg | 2.73 ± 0.50^d^ | 1.46 ± 0.45^de^ | 1.02 ± 0.23^e,*^ | 10^3^ | 0.03 | 0.19 |
|  | Old | 9.17 ± 2.52^d^ | 3.11 ± 0.80^de^ | 3.04 ± 0.51^e,*^ |  |  |  |
| PC(30:0) | Yg | 5.88 ± 0.68^d,+^ | 2.89 ± 0.75^de^ | 3.09 ± 0.21^e,*^ | 10^4^ | <0.001 | 0.24 |
|  | Old | 9.65 ± 1.30^a,+^ | 3.88 ± 0.47^b^ | 5.76 ± 0.69^b,*^ |  |  |  |
| PC(30:1) | Yg | 1.55 ± 0.14 | 2.49 ± 0.36 | 2.81 ± 0.54^*^ | 10^4^ | <0.001 | 0.26 |
|  | Old | 1.99 ± 0.23^a^ | 3.14 ± 0.50^ab^ | 4.58 ± 0.46^b,*^ |  |  |  |
|  |  |  |  |  |  |  |  |
|  |  |  |  |  |  |  |  |
| PC(32:0) | Yg | 2.57 ± 0.27 | 1.20 ± 0.35 | 2.40 ± 0.54 | 10^5^ | <0.001 | 0.73 |
|  | Old | 3.00 ± 0.25^a^ | 1.19 ± 0.19^b^ | 2.41 ± 0.22^a^ |  |  |  |
| PC(32:1) | Yg | 3.56 ± 0.18^d^ | 2.59 ± 0.29^de^ | 2.99 ± 0.09^e^ | 10^5^ | <0.001 | 0.85 |
|  | Old | 3.95 ± 0.14^a^ | 2.94 ± 0.15^b^ | 3.58 ± 0.29^ab^ |  |  |  |
| PC(32:2) | Yg | 4.90 ± 0.59^a,*^ | 3.05 ± 0.73^ab^ | 1.82 ± 0.29^b,+^ | 10^4^ | <0.001 | 0.16 |
|  | Old | 7.76 ± 0.88^a,*^ | 3.85 ± 0.50^b^ | 3.09 ± 0.42^b,+^ |  |  |  |
| PC(34:0) | Yg | 2.96 ± 0.11 | 2.72 ± 0.12 | 2.52 ± 0.21 | 10^5^ | 0.01 | 0.63 |
|  | Old | 2.91 ± 0.07^a^ | 2.38 ± 0.17^ab^ | 2.19 ± 0.20^b^ |  |  |  |
| PC(34:1) | Yg | 1.62 ± 0.15 | 1.46 ± 0.75^*^ | 1.65 ± 0.18 | 10^5^ | <0.01 | 0.02 |
|  | Old | 1.63 ± 0.15 | 1.61 ± 0.11^*^ | 1.64 ± 0.24 |  |  |  |
| PC(O-34:2) | Yg | 2.73 ± 0.09^d^ | 2.82 ± 0.54^de^ | 1.60 ± 0.37^e^ | 10^4^ | 0.01 | 0.42 |
|  | Old | 2.41 ± 3.57 | 2.39 ± 2.54 | 1.85 ± 1.80 |  |  |  |
| PC(34:3) | Yg | 11.63 ± 1.55^a,d^ | 7.84 ± 1.47^b,d^ | 5.54 ± 1.39^ab,e^ | 10^4^ | <0.001 | 0.62 |
|  | Old | 12.52 ± 0.86^a^ | 8.22 ± 0.94^b^ | 7.81 ± 0.96^b^ |  |  |  |
| PC(35:3) | Yg | 2.54 ± 0.18^d^ | 1.67 ± 0.29^e^ | 1.53 ± 0.20^de^ | 10^4^ | <0.001 | 0.39 |
|  | Old | 2.52 ± 0.20^a^ | 1.62 ± 0.14^b^ | 1.97 ± 0.20^ab^ |  |  |  |
| PC(35:4) | Yg | 4.20 ± 0.39^a,de^ | 2.18 ± 0.49^b,d,*^ | 6.19 ± 1.38^ab,e^ | 10^3^ | <0.001 | 0.50 |
|  | Old | 4.68 ± 0.73^ab,d^ | 4.57 ± 0.72^a,de,*^ | 8.48 ± 0.91^b,e^ |  |  |  |
| PC(36:2) | Yg | 8.38 ± 0.29 | 9.82 ± 1.14 | 7.85 ± 0.22 | 10^5^ | 0.01 | 0.25 |
|  | Old | 7.83 ± 0.32^ab^ | 8.45 ± 0.19^a^ | 7.89 ± 0.20^b^ |  |  |  |
| PC(36:3) | Yg | 5.86 ± 0.21 | 5.30 ± 0.52 | 5.28 ± 025 | 10^5^ | 0.03 | 0.83 |
|  | Old | 5.98 ± 0.19^a^ | 5.07 ± 0.20^b^ | 5.18 ± 0.27^ab^ |  |  |  |
| PC(36:4) | Yg | 8.71 ± 1.11^a^ | 6.96 ± 1.01^ab^ | 3.63 ± 0.39^b^ | 10^4^ | <0.001 | 0.22 |
|  | Old | 7.99 ± 0.47^a^ | 5.39 ± 0.35^b^ | 4.68 ± 0.76^b^ |  |  |  |
| PC(36:4) | Yg | 1.91 ± 0.17^a,+^ | 1.71 ± 0.27^a^ | 3.89 ± 0.32^b^ | 10^5^ | <0.001 | 0.51 |
|  | Old | 2.51 ± 0.20^a,+^ | 2.65 ± 0.42^a^ | 4.17 ± 0.25^b^ |  |  |  |
| PC(36:5) | Yg | 10.94 ± 1.61^de^ | 11.55 ± 2.58^d^ | 7.02 ± 1.38^e^ | 10^4^ | 0.04 | 0.50 |
|  | Old | 10.05 ± 1.43^a^ | 7.20 ± 1.58^ab^ | 4.73 ± 1.07^b^ |  |  |  |
| PC(36:5) | Yg | 6.99 ± 0.74 | 4.98 ± 0.89 | 8.18 ± 2.33 | 10^4^ | 0.03 | 0.89 |
|  | Old | 7.44 ± 0.67 | 6.44 ± 0.72 | 9.41 ± 1.18 |  |  |  |
| PC(37:5) | Yg | 3.88 ± 0.56 | 3.01 ± 0.54^*^ | 6.37 ± 1.77 | 10^3^ | <0.01 | 0.83 |
|  | Old | 5.43 ± 0.78^ab^ | 4.47 ± 0.38^a,*^ | 8.55 ± 0.92^b^ |  |  |  |
| PC(38:3) | Yg | 6.51 ± 0.98 | 5.05 ± 0.92 | 7.45 ± 0.51 | 10^4^ | <0.01 | 0.30 |
|  | Old | 6.44 ± 0.21^a,de^ | 6.47 ± 0.34^ab,d^ | 9.74 ± 1.02^b,e^ |  |  |  |
| PC(38:4) | Yg | 3.51 ± 0.61^de^ | 2.29 ± 0.30^d^ | 4.01 ± 0.46^e^ | 10^4^ | 0.01 | 0.84 |
|  | Old | 4.17 ± 0.52^ab,d^ | 2.74 ± 0.27^a,de^ | 4.90 ± 0.64^b,e^ |  |  |  |
| PC(38:5) | Yg | 12.57 ± 1.27^ab,*^ | 9.24 ± 1.95^a,+^ | 23.95 ± 3.36^b^ | 10^4^ | <0.001 | 0.16 |
|  | Old | 17.11 ± 1.16^*^ | 14.86 ± 2.11^+^ | 22.15 ± 1.68 |  |  |  |
| PC(38:6) | Yg | 7.50 ± 0.33^a^ | 6.25 ± 1.01^a^ | 3.73 ± 0.47^b^ | 10^4^ | <0.001 | 0.90 |
|  | Old | 7.05 ± 0.35^a^ | 5.58 ± 0.75^ab^ | 3.36 ± 0.62^b^ |  |  |  |
| PC(39:1) | Yg | 5.89 ± 0.94^a^ | 3.46 ± 1.03^b^ | 3.47 ± 0.76^ab^ | 10^3^ | 0.03 | 0.51 |
|  | Old | 9.48 ± 2.53 | 6.18 ± 1.18 | 3.84 ± 0.74 |  |  |  |
| PC(P-39:1) | Yg | 15.61 ± 0.27 | 14.01 ± 1.95 | 20.06 ± 2.26 | 10^4^ | <0.001 | 0.17 |
|  | Old | 15.15 ± 1.24^a^ | 15.00 ± 1.02^a^ | 23.74 ± 1.23^b^ |  |  |  |
| PE(36:1) | Yg | 12.02 ± 0.46^a^ | 6.93 ± 0.76^b^ | 8.69 ± 1.24^ab^ | 10^4^ | <0.001 | 0.05 |
|  | Old | 10.11 ± 0.84^d^ | 8.26 ± 0.51^e^ | 9.72 ± 0.81^de^ |  |  |  |
| PE(36:3) | Yg | 6.46 ± 0.22^a,*^ | 7.45 ± 1.41^ab,+^ | 4.66 ± 0.34^b,*^ | 10^4^ | 0.01 | 0.32 |
|  | Old | 5.62 ± 0.21^a,*^ | 4.81 ± 0.63^ab,+^ | 3.40 ± 0.36^b,*^ |  |  |  |
| PE(38:4) | Yg | 11.31 ± 0.47 | 8.16 ± 1.15 | 9.69 ± 1.55 | 10^3^ | 0.03 | 0.53 |
|  | Old | 10.65 ± 0.96^e^ | 7.85 ± 1.33^de^ | 7.04 ± 0.66^e^ |  |  |  |
| PE(42:0) | Yg | 16.17 ± 3.35^d^ | 7.47 ± 1.52^e,*^ | 9.14 ± 1.02^de^ | 10^3^ | <0.01 | 0.16 |
|  | Old | 15.88 ± 2.27 | 12.54 ± 0.88^*^ | 14.22 ± 2.48 |  |  |  |
| PE(42:2) | Yg | 1.28 ± 0.09 | 1.57 ± 0.20 | 2.26 ± 0.44 | 10^4^ | <0.001 | 0.47 |
|  | Old | 1.95 ± 0.29^a^ | 1.80 ± 0.15^a^ | 3.05 ± 0.27^b^ |  |  |  |
| SM(24:0) | Yg | 2.49 ± 0.36 | 2.09 ± 0.38 | 3.29 ± 0.80^+^ | 10^4^ | <0.01 | 0.08 |
|  | Old | 2.51 ± 0.24^a^ | 2.83 ± 0.30^a^ | 5.89 ± 0.89^b,+^ |  |  |  |
| SM(d32:1) | Yg | 3.64 ± 0.50 | 4.68 ± 0.83 | 5.15 ± 0.64^*^ | 10^3^ | <0.001 | 0.05 |
|  | Old | 4.53 ± 0.40^a^ | 4.51 ± 0.61^a^ | 8.24 ± 0.64^b,*^ |  |  |  |
| SM(d34:1) | Yg | 1.16 ± 0.11^a^ | 1.63 ± 0.32^ab^ | 2.02 ± 0.20^b^ | 10^5^ | <0.001 | 0.28 |
|  | Old | 1.06 ± 0.03^a^ | 1.40 ± 0.10^b^ | 2.33 ± 0.21^c^ |  |  |  |
| TG(52:5) | Yg | 2.14 ± 0.31 | 2.77 ± 0.53 | 1.71 ± 0.28 | 10^4^ | 0.01 | 0.84 |
|  | Old | 2.54 ± 0.26^d^ | 3.47 ± 0.52^e^ | 1.97 ± 0.23^de^ |  |  |  |
| TG(54:5) | Yg | 6.89 ± 0.72 | 10.24 ± 1.28 | 6.72 ± 0.62 | 10^4^ | 0.01 | 0.81 |
|  | Old | 6.64 ± 1.59^a^ | 9.63 ± 1.03^b^ | 5.41 ± 1.05^ab^ |  |  |  |
| TG(54:7) | Yg | 1.09 ± 0.25 | 1.28 ± 0.16^+^ | 1.58 ± 0.21 | 10^4^ | 0.17 | 0.42 |
|  | Old | 1.39 ± 0.15^a^ | 1.99  ± 0.25^b,+^ | 1.68 ± 0.21^ab^ |  |  |  |
| TG(55:2) | Yg | 1.00 ± 0.15^a^ | 1.69 ± 0.12^b^ | 1.55 ± 0.31^ab^ | 10^5^ | 0.04 | 0.71 |
|  | Old | 0.85 ± 0.12^a^ | 1.68 ± 0.24^b^ | 1.24 ± 0.21^ab^ |  |  |  |
| TG(56:4) | Yg | 8.35 ± 0.82^a^ | 22.17 ± 1.45^b^ | 26.22 ± 3.71^b^ | 10^3^ | 0.01 | 0.70 |
|  | Old | 7.06 ± 0.94^a,d^ | 26.36 ± 4.67^b,de^ | 26.07 ± 5.83^ab,e^ |  |  |  |
| TG(56:5) | Yg | 1.89 ± 0.13^a^ | 4.31 ± 0.32^b,*^ | 6.55 ± 0.90^b^ | 10^4^ | <0.01 | 0.48 |
|  | Old | 2.18 ± 0.21^a,d^ | 6.05 ± 0.56^b,de,*^ | 6.41 ± 1.28^ab,e^ |  |  |  |
|  |  |  |  |  |  |  |  |
|  |  |  |  |  |  |  |  |
| TG(56:7) | Yg | 1.65 ± 0.34^a^ | 4.51 ± 0.50^b^ | 8.89 ± 1.56^b^ | 10^4^ | <0.001 | 0.16 |
|  | Old | 2.14 ± 0.24^a^ | 5.41 ± 0.34^b^ | 6.54 ± 0.96^b^ |  |  |  |
| TG(56:8) | Yg | 0.80 ± 0.22^a,d^ | 2.17 ± 0.39^ab,e^ | 3.53 ± 0.48^b,de^ | 10^4^ | <0.001 | 0.53 |
|  | Old | 1.14 ± 0.15^a^ | 2.50 ± 0.22^b^ | 3.18 ± 0.50^b^ |  |  |  |
| TG(58:5) | Yg | 2.39 ± 0.20^a^ | 7.74 ± 1.10^b^ | 12.47 ± 2.44^b^ | 10^3^ | 0.01 | 0.59 |
|  | Old | 2.65 ± 0.36^a,d^ | 11.00 ± 2.08^b,de^ | 11.93 ± 2.84^ab,e^ |  |  |  |
| TG(58:6) | Yg | 0.64 ± 0.09^a^ | 1.98 ± 0.22^b^ | 3.30 ± 0.47^b^ | 10^4^ | <0.01 | 0.65 |
|  | Old | 0.75 ± 0.11^a,d^ | 2.40 ± 0.26^b,de^ | 3.03 ± 0.71^ab,e^ |  |  |  |
| TG(58:8) | Yg | 2.07 ± 0.29^a^ | 3.49 ± 0.17^b^ | 3.27 ± 0.59^ab^ | 10^4^ | 0.03 | 0.53 |
|  | Old | 2.23 ± 0.15^a^ | 3.36 ± 0.23^b^ | 2.69 ± 0.39^ab^ |  |  |  |
| TG(58:9) | Yg | 3.06 ± 0.81^a^ | 10.91 ± 0.47^b^ | 36.41 ± 5.62^c^ | 10^3^ | <0.001 | 0.30 |
|  | Old | 4.15 ± 0.54^a^ | 12.23 ± 1.52^b^ | 29.41 ± 5.20^c^ |  |  |  |
| TG(62:14) | Yg | 6.09 ± 1.31^a,de^ | 23.41 ± 4.04^b,d^ | 60.97 ± 8.59^b,e^ | 10^2^ | <0.001 | 0.15 |
|  | Old | 9.88 ± 2.26^a,de^ | 21.72 ± 2.58^b,d^ | 41.91 ± 8.31^b,e^ |  |  |  |
| Subclass: Ceramides and related molecules | | | | |  |  |  |
| C18 Ceramide (d18:1/18:0) | Yg | 10.81 ± 1.63 | 9.68 ± 1.59 | 6.47 ± 0.56 | 10^3^ | 0.04 | 0.32 |
|  | Old | 8.23 ± 1.10 | 10.61 ± 1.26 | 7.21 ± 1.31 |  |  |  |
| Cer(d34:0) | Yg | 1.34 ± 0.27 | 2.96 ± 0.48 | 2.61 ± 0.59 | 10^3^ | <0.001 | 0.40 |
|  | Old | 1.19 ± 0.11^a^ | 3.99 ± 0.70^b^ | 2.99 ± 0.50^c^ |  |  |  |
| Cer(d41:1) | Yg | 2.58 ± 0.48^ab^ | 1.54 ± 0.25^a,+^ | 2.11 ± 0.35^b^ | 10^3^ | 0.29 | 0.90 |
|  | Old | 2.02 ± 0.23 | 2.33 ± 0.29^+^ | 2.78 ± 0.40 |  |  |  |
| GalCer(d38:1) | Yg | 15.44 ± 2.16^a,d^ | 8.80 ± 1.75^ab,e^ | 8.16 ± 1.09^b,de^ | 10^3^ | <0.001 | 0.73 |
|  | Old | 14.05 ± 1.68^a^ | 9.83 ± 1.57^ab^ | 7.67 ± 0.85^b^ |  |  |  |
| GalCer(d42:2) | Yg | 11.24 ± 1.95^a^ | 5.06 ± 1.1^b,*^ | 5.59 ± 0.82^ab^ | 10^3^ | 0.03 | 0.43 |
|  | Old | 13.89 ± 3.05 | 10.51 ± 1.54^*^ | 12.85 ± 2.67 |  |  |  |
| GlcCer(d34:1) | Yg | 9.78 ± 2.12^a^ | 6.04 ± 1.83^b^ | 4.76 ± 0.60^ab^ | 10^3^ | 0.001 | 0.66 |
|  | Old | 9.88 ± 1.70^d^ | 8.01 ± 0.99^de^ | 6.21 ± 1.22^e^ |  |  |  |
| Amino Acids and Derivatives | | | |  |  |  |  |
| α‐ketobutyrate | Yg | 1.55 ± 0.45^a^ | 13.46 ± 5.83^ab^ | 13.51 ± 2.67^b^ | 10^3^ | 0.001 | 0.27 |
|  | Old | 2.17 ± 0.58^a,d^ | 6.50 ± 1.31^b,de^ | 8.92 ± 2.29^ab,e^ |  |  |  |
| Alanine (2TMS) | Yg | 5.77 ± 0.94^a^ | 13.78 ± 3.71^a,*^ | 39.61 ± 7.14^b^ | 10^6^ | <0.001 | 0.22 |
|  | Old | 4.76 ± 0.62^a^ | 5.04 ± 0.48^a,*^ | 41.94 ± 3.80^b^ |  |  |  |
| Cysteine (3TMS) | Yg | 3.20 ± 2.13 | 2.49 ± 1.32^+^ | 45.18 ± 17.81 | 10^5^ | <0.001 | 0.27 |
|  | Old | 1.90 ± 0.38^a^ | 8.59 ± 2.28^b,+^ | 74.95 ± 13.23^c^ |  |  |  |
| Cysteinyl-Proline | Yg | 2.04 ± 1.47 | 4.01 ± 1.48 | 52.69 ± 27.04 | 10^2^ | <0.01 | 0.29 |
|  | Old | 0.33 ± 0.09^a,d^ | 2.98 ± 0.80^a,e^ | 28.21 ± 2.83^b,de^ |  |  |  |
| ɛ-polylysine | Yg | 31.74 ± 6.12^a^ | 49.89 ± 8.37^a^ | 3.43 ± 0.59^b^ | 10^3^ | <0.001 | 0.87 |
|  | Old | 26.21 ± 9.35^ab^ | 53.72 ± 12.02^a^ | 4.84 ± 0.83^b^ |  |  |  |
| Glutamic acid (3TMS) | Yg | 0.59 ± 0.17^d,*^ | 0.80 ± 0.41^d,+^ | 14.79 ± 2.03^e^ | 10^6^ | <0.001 | 0.21 |
|  | Old | 1.50 ± 0.21^a,*^ | 1.56 ± 0.23^a,+^ | 23.04 ± 3.52^b^ |  |  |  |
| Glycine (3TMS) | Yg | 2.00 ± 0.14^a,*^ | 2.76 ± 0.45^a,+^ | 10.87 ± 1.60^b^ | 10^7^ | <0.001 | 0.19 |
|  | Old | 2.84 ± 0.10^a,d,*^ | 3.97 ± 0.44^a,e,+^ | 15.89 ± 1.97^b,de^ |  |  |  |
| Glycine (2TMS) | Yg | 5.96 ± 0.77^a^ | 3.60 ± 1.14^ab^ | 1.87 ± 0.26^b^ | 10^7^ | <0.001 | 0.98 |
|  | Old | 5.65 ± 0.28^a,de^ | 3.22 ± 0.53^b,d^ | 1.58 ± 0.23^b,e^ |  |  |  |
| Glycyl-Tyrosine | Yg | 42.50 ± 5.53^a^ | 52.41 ± 5.37^b^ | 2.76 ± 0.45^c^ | 10^4^ | <0.001 | 0.51 |
|  | Old | 30.35 ± 7.77^a^ | 41.99 ± 5.86^a^ | 3.03 ± 4.63^b^ |  |  |  |
| Pyroglutamic acid (2TMS) | Yg | 0.56 ± 0.15^a,d^ | 1. 67 ± 0.38^a,e^ | 10.72 ± 0.53^b,de^ | 10^7^ | <0.001 | 0.10 |
|  | Old | 0.95 ± 0.14^a^ | 1.20 ± 0.11^a^ | 12.57 ± 0.77^b^ |  |  |  |
| Serine (3TMS) | Yg | 0.74 ± 0.21^a^ | 0.73 ± 0.24^a^ | 9.93 ± 0.77^b^ | 10^6^ | <0.001 | 0.22 |
|  | Old | 0.63 ± 0.10^a^ | 0.43 ± 0.07^a^ | 11.34 ± 0.79^b^ |  |  |  |
| Threonine (3TMS) | Yg | 3.44 ± 1.17^a^ | 3.92 ± 1.75^a^ | 68.39 ± 6.89^b^ | 10^5^ | <0.001 | 0.52 |
|  | Old | 1.97 ± 0.17^a^ | 2.87 ± 0.56^a^ | 74.87 ± 7.20^b^ |  |  |  |
| Miscellaneous |  |  |  |  |  |  |  |
| 2-acetoxy-xxx-benzoic acid^4^ | Yg | 6.49 ± 2.20^a^ | 10.59 ± 4.71^ab^ | 21.60 ± 1.41^b^ | 10^3^ | 0.03 | 0.38 |
|  | Old | 13.44 ± 7.05^ab^ | 5.82 ± 1.99^a^ | 20.85 ± 1.91^b^ |  |  |  |
| (2R)-2xxx- heptadecanoate^5^ | Yg | 4.11 ± 0.88^a,d,+^ | 1.73 ± 0.33^a,e^ | 12.98 ± 1.51^b,de^ | 10^3^ | <0.001 | 0.09 |
|  | Old | 6.62 ± 0.83^ab,d,+^ | 3.41 ± 0.79^a,e^ | 10.69 ± 1.55^b,de^ |  |  |  |
| (3aS,5aS,5bR-xxx-chrysene^6^ | Yg | 1.59 ± 0.47 | 20.37 ± 11.75 | 3.56 ± 1.40 | 10^3^ | 0.05 | 0.14 |
|  | Old | 1.98 ± 0.57 | 5.29 ± 2.07 | 2.65 ± 0.77 |  |  |  |
| (3beta)-xxx-icosatetraenoate^7^ | Yg | 1.09 ± 0.17^a,*^ | 12.15 ± 6.50^a^ | 103.31 ± 23.98^b,+^ | 10^2^ | <0.001 | 0.05 |
|  | Old | 2.85 ± 0.60^a,*^ | 4.69 ± 1.14^a^ | 51.77 ± 11.71^b,+^ |  |  |  |
| 9-(2aS,4R,7R,8aS)-xxx-carboxylate^8^ | Yg | 25.07 ± 6.81 | 7.47 ± 3.35 | 10.25 ± 2.53 | 10^3^ | <0.001 | 0.92 |
|  | Old | 24.33 ± 2.48^a^ | 7.68 ± 1.06^b^ | 9.60 ± 0.89^b^ |  |  |  |
| [(E,2S,3R)-xxx- phosphate^9^ | Yg | 4.34 ± 0.35 | 6.90 ± 1.41 | 7.15 ± 1.11 | 10^3^ | <0.001 | 0.14 |
|  | Old | 3.52 ± 0.34^a^ | 5.89 ± 0.47^b^ | 9.42 ± 1.28^b^ |  |  |  |
| 9-xxx-N2-Phenylguanine^10^ | Yg | 4.76 ± 1.41 | 6.43 ± 1.87^*^ | 4.21 ± 1.30 | 10^3^ | 0.08 | 0.03 |
|  | Old | 4.44 ± 0.43^a,d^ | 2.68 ± 0.37^ab,e,*^ | 2.52 ± 0.49^b,de^ |  |  |  |
| 9-xxx-N2-Phenylguanine^10^ | Yg | 4.10 ± 0.50 | 6.47 ± 1.87^+^ | 4.05 ± 1.43 | 10^3^ | 0.1 | 0.05 |
|  | Old | 4.16 ± 0.65 | 3.19 ± 0.52^+^ | 2.77 ± 0.55 |  |  |  |
| Adenosine (3TMS)^11^ | Yg | 8.07 ± 2.69^*^ | 8.51 ± 2.34 | 13.25 ± 4.39^+^ | 10^5^ | 0.02 | 0.19 |
|  | Old | 14.55 ± 1.65^d,*^ | 13.21 ± 3.63^de^ | 34.97 ± 7.63^e,+^ |  |  |  |
| Adenosine (3TMS)^11^ | Yg | 17.72 ± 9.61 | 17.15 ± 6.95 | 13.08 ± 8.68 | 10^4^ | 0.05 | 0.06 |
|  | Old | 7.63 ± 0.62^a^ | 47.56 ± 12.80^b^ | 14.56 ± 7.50^a^ |  |  |  |
| Adenosine | Yg | 2.33 ± 0.83^a,de^ | 2.94 ± 0.51^ab,d^ | 12.35 ± 2.44^b,e^ | 10^3^ | <0.001 | 0.23 |
|  | Old | 2.94 ± 0.31^a^ | 1.99 ± 0.62^a^ | 15.89 ± 1.74^b^ |  |  |  |
| dihydroxy-tetranorvitamin D3 | Yg | 7.90 ± 1.98 | 2.53 ± 0.63 | 6.50 ± 0.73 | 10^3^ | 0.02 | 0.55 |
|  | Old | 7.55 ± 0.97^ab^ | 5.15 ± 1.11^a^ | 8.00 ± 1.78^b^ |  |  |  |
| Phosphoric acid (3TMS) | Yg | 13.97 ± 2.76^a,*^ | 17.21 ± 5.44^ab^ | 5.50 ± 1.46^b^ | 10^7^ | 0.01 | 0.32 |
|  | Old | 25.42 ± 1.97^a,*^ | 30.72 ± 6.54^a^ | 7.57 ± 0.51^b^ |  |  |  |
| Phosphoric acid monomethyl ester^12^ | Yg | 1.51 ± 0.42 | 2.37 ± 0.94^*^ | 3.05 ± 0.67^+^ | 10^7^ | 0.01 | 0.08 |
|  | Old | 2.40 ± 0.31^a^ | 6.54 ± 0.96^b,*^ | 3.37 ± 0.31^a,+^ |  |  |  |
| Putrescine (3TMS) | Yg | 38.68 ± 18.99 | 1.05 ± 0.57 | 13.60 ± 9.52 | 10^6^ | 0.02 | 0.37 |
|  | Old | 77.57 ± 25.38^d^ | 20.41 ± 8.82^de^ | 9.26 ± 1.80^e^ |  |  |  |
| Pyridine, 2-hydroxy- (1TMS) | Yg | 43.91 ± 7.50^d^ | 46.51 ± 7.63^d^ | 9.40 ± 2.34^e^ | 10^7^ | <0.001 | 0.22 |
|  | Old | 51.48 ± 2.22^a^ | 34.62 ± 5.63^a^ | 8.13 ± 2.17^b^ |  |  |  |
| Pyridoxamine (3TMS) | Yg | 3.07 ± 0.46^ab*^ | 2.55 ± 0.56^a^ | 1.11 ± 0.37^b^ | 10^5^ | <0.001 | 0.07 |
|  | Old | 4.80 ± 0.50^a,de,*^ | 2.33 ± 0.43^b,d^ | 0.89 ± 0.22^b,e^ |  |  |  |
| Raoline | Yg | 7.89 ± 1.00^a^ | 11.87 ± 0.27^b^ | 10.86 ± 1.98^ab^ | 10^4^ | 0.08 | 0.60 |
|  | Old | 5.71 ± 0.73^a^ | 13.11 ± 2.70^b^ | 9.44 ± 1.94^ab^ |  |  |  |

^1^Inositol-2-phosphate, myo- (7TMS), ^2^4-Hydroxy-3-[(1E)-5-oxo-1,7-diphenyl-1-hepten-3-yl]-6-[(E)-2-phenylvinyl]-2H-pyran-2-one; ^3^Octadecadienoic acid, 9,12-(Z,Z)- (1TMS); ^4^2-acetoxy-6-pentadecyl-benzoic acid; ^5^(2R)-2-(Palmitoyloxy)-3-(phosphonooxy)propyl heptadecanoate; ^6^(3aS,5aS,5bR,7aS,11aS,11bR)-3a,5a,5b,8,8,11a-hexamethyl-1,2,3,4,5,6,7,7a,9,10,11,11b,12,13-tetradecahydrocyclopenta[a]chrysene; ^7^(3beta)-Cholest-5-en-3-yl (5Z,8Z,11Z,14Z)-5,8,11,14-icosatetraenoate; ^8^9-[(2aS,4R,7R,8aS)-6-Hydroxy-7-methyl-2,2a,3,4,6,7,8,8a-octahydro-1H-5,6,8b-triazaacenaphthylen-4-yl]-2-nonanyl (2aS,3S,4R,8aS)-4-methyl-7-nonyl-2,2a,3,4,6,7,8,8a-octahydro-1H-5,6,8b-triazaacenaphthylene-3-carboxylate;^9^[(E,2S,3R)-3-hydroxy-2-(pentadecanoylamino)octadec-4-enyl] 2-(trimethylammonio)ethyl phosphate; ^10^9-(4-Hydroxybutyl)-N2-Phenylguanine; ^11^Adenosine (3TMS) (Derivate not found); ^12^Phosphoric acid monomethyl ester (2TMS)
